# Structural analysis of stereoselective galactose pyruvylation toward the synthesis of bacterial capsular polysaccharides

**DOI:** 10.3762/bjoc.21.131

**Published:** 2025-08-21

**Authors:** Tsun-Yi Chiang, Mei-Huei Lin, Chun-Wei Chang, Jinq-Chyi Lee, Cheng-Chung Wang

**Affiliations:** 1 Institute of Chemistry, Academia Sinica, Taipei 115, Taiwan; 2 Institute of Biotechnology and Pharmaceutical Research, National Health Research Institutes, Miaoli Country 35053, Taiwan; 3 Chemical Biology and Molecular Biophysics Program, Taiwan International Graduate Program (TIGP), Academia Sinica, Taipei 115, Taiwan

**Keywords:** carbohydrates, capsular polysaccharides, diastereochemistry, glycosylation, pyruvate ketals

## Abstract

Pyruvate ketal is a biologically essential moiety due to its key role as an intermediate in metabolic pathways, serving as a key precursor for the synthesis of various essential biomolecules in organisms. However, the *R*/*S* stereochemistry of pyruvate ketal is difficult to control through chemical methods. In this study, the acid-labile pyruvate ketal linked to the 4- and 6-positions of galactose was cautiously constructed, and the X-ray analysis of the *R*-configured product was successfully obtained. Subsequently, the compound was used for the synthesis of zwitterionic polysaccharide A1 (PS A1) precursor, and a clear structural elucidation was applied by using nuclear magnetic resonance and X-ray.

## Introduction

**Figure 1:**
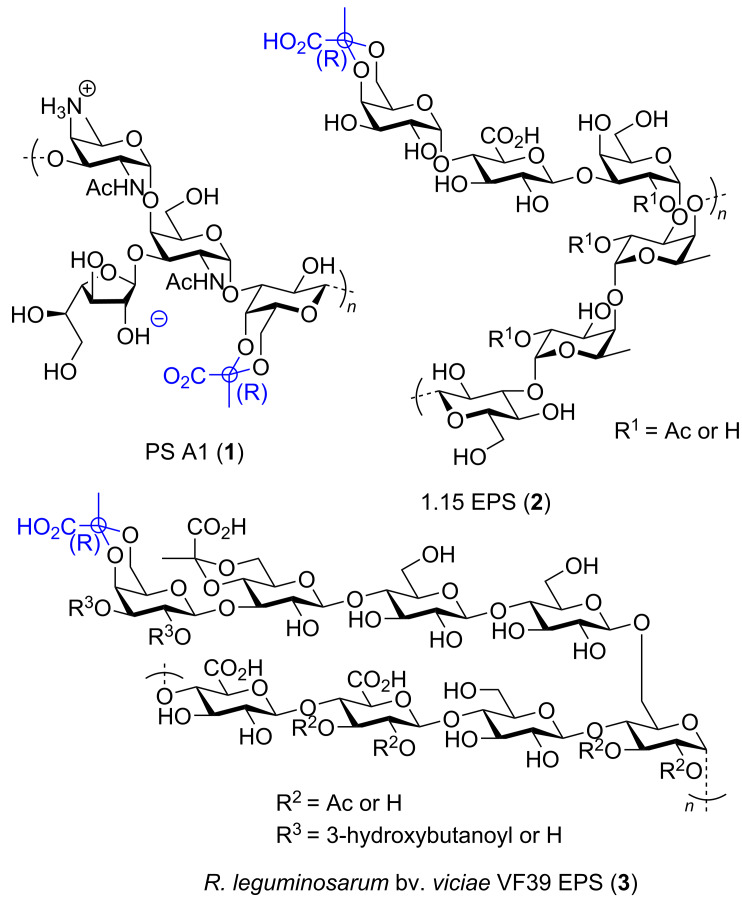
Pyruvylated galactose on bacterial polysaccharides PS A1 (**1**), 1.15 EPS (**2**) and *Rhizobium leguminosarum* bv. *viciae* VF39 EPS (**3**).

Pyruvate ketals are essential non-carbohydrate modifications in polysaccharides from bacteria and serve as a key immunodominant group with many biological functions [1]. For example, the pyruvylated galactose moiety is a key component of *Bacteroides fragilis* capsular polysaccharide (PS A1) (**1**), which has been shown by Andreana et al. to significantly activate the immune system and therefore recognized as a promising candidate for cancer immunotherapy [2–5]. This distinct structure is crucial for regulating the transportation of substances across cell membranes and facilitating cell–cell interactions [1]. Additionally, various glycans containing pyruvate ketals have been identified, including those found in *Pseudomonas strain* 1.15 (1.15 EPS) (**2**) and *Rhizobium leguminosarum* (*R. leguminosarum* bv. *viciae* VF39 EPS) (**3**) (Figure 1). These exopolysaccharides have been well-studied and are considered good candidates for drug development [1,6].

The synthesis of bacterial capsular polysaccharides, particularly in the installation of the pyruvate ketal on galactose, presents significant challenges. Owing to pyruvate ketals are considered immunogenic, non carbohydrate modifications of bacterial polysaccharides, and the interaction of 4,6-*O*-pyruvylated pyranoses with lectins depends on the stereochemistry of the pyruvate ketal. Moreover, this chemical modification is crucial, as the distinct *R* or *S* diastereochemistry at the ketal center directly influences its biological function. Careful manipulation of reaction conditions such as solvent and temperature is necessary to achieve sufficient diastereomeric purity in pyruvate modification. This research contributes to the development in the field of polysaccharide synthesis and draws upon established methodologies, reflecting the foundational work of Kulkarni, Codée, Boons, and other outstanding groups in their studies on zwitterionic polysaccharides [7–12].

Pyruvylation of carbohydrate diols is commonly achieved through acetal substitution in the presence of acid during chemical synthesis. In this process, pyruvate ketals are attached to the O4 and O6 positions of galactose, incorporating a negatively charged carboxyl group. The diastereoselectivity and negative charge of pyruvate ketals have a key role in their biological interaction, such as influencing immunological specificity and patterns of immunological cross-reactivity. Moreover, pyruvate ketals are recognized as immunodominant structural features of polysaccharides, which are important in cell–cell recognition processes [13–18]. The pyruvate ketal is an important portion of immunological determinant in bacterial polysaccharides, and the immunological properties may base on *R*/*S*-configuration. However, precisely controlling the formation of *R*- and *S*-isomers and identifying the correct conformer can be challenging due to the structural similarities between these two isomers. Researchers have recently developed strategies to define the stereochemistry of pyruvate ketals as either *R*- or *S*-configurations. Typically, *S*-configuration exhibits ^13^C NMR shifts between δ 17–24 ppm and ^1^H NMR shifts δ 1.60–2.10 ppm, whereas the *R*-configuration shows ^13^C shifts downfield at δ 24-27 ppm and ^1^H shifts at δ 1.40–2.00 ppm [19–27]. However, these chemical shifts can vary depending on the molecular structure, making it difficult to characterize the isomers across different systems. To address this, the current study aimed to develop a method for installing pyruvate ketals with precise diastereochemistry and confirming their structure by using X-ray crystallography as direct evidence. Subsequently, the pyruvylated galactose was further applied in the synthesis of zwitterionic polysaccharide A1 (PS A1) precursor.

## Results and Discussion

In the initial experiment, *p*-tolyl 2,3-di-*O*-acetyl-1-thio-β-galactopyranoside (**4**) was treated with methyl pyruvate in acetonitrile (Table 1, entry 1) to produce pyruvylated galactose **5**. The use of polar solvents such as acetonitrile promotes the formation of the more thermodynamically favorable diastereomer [21,28–29]. For the property of the *R*-isomer of **5**, the ^13^C NMR signal shifted to 25.7 ppm, and the ^1^H signal shifted to 1.57 ppm. However, under these conditions, the pyruvylation proceeded sluggishly, with incomplete conversion even after 18.5 h, resulting in low yields of 17–22% (Table 1, entries 1 and 2). Additionally, extending the reaction time led to an undesired anomeric epimerization, leading to the production of two stereoisomers in a ratio of α/β = 1:1.6 [28–29]. To increase the yield and minimize anomeric epimerization, we employed the more reactive reagent, methyl 2,2-dimethoxypropanoate, which contains a hemiketal moiety. As expected, the reaction was significantly faster, and the starting material was completely consumed within 2 h, followed by deacetylation to yield the desired product **6** in 51% (Table 1, entry 3). The use of this more reactive reagent maintained high *R*-diastereoselectivity. It is hypothesized that this reaction will yield a greater number of compounds compared to the use of methyl pyruvate. According to the second law of thermodynamics, the enhanced pyruvylation efficacy can be attributed to an entropy-driven effect, wherein a diol reacted with 1.0 equivalent of 2,2-dimethoxypropanoate to produce 2.0 equivalents of methanol byproducts. This increased the entropy and drove the reaction forward, facilitating the formation of the product.

**Table 1:** Optimization of reaction conditions towards the synthesis of (*R*)-pyruvylated galactose **5** and **6**.

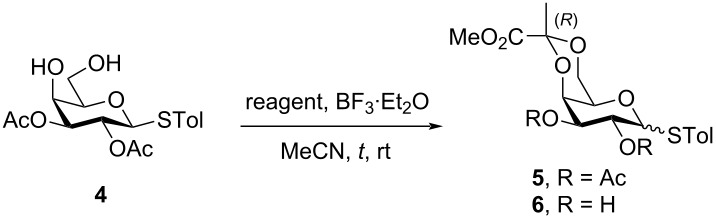					

Entry	S.M.	Reagent	*t* (h)	Product	Yield^a^

1	**4**	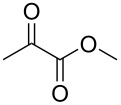	2	**5**	17%
2	**4**		18.5	**5**	22% (α/β = 1:1.6)

3	**4**	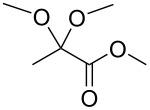	2	**6**	51%

^a^Isolated yield.

**Figure 2:**
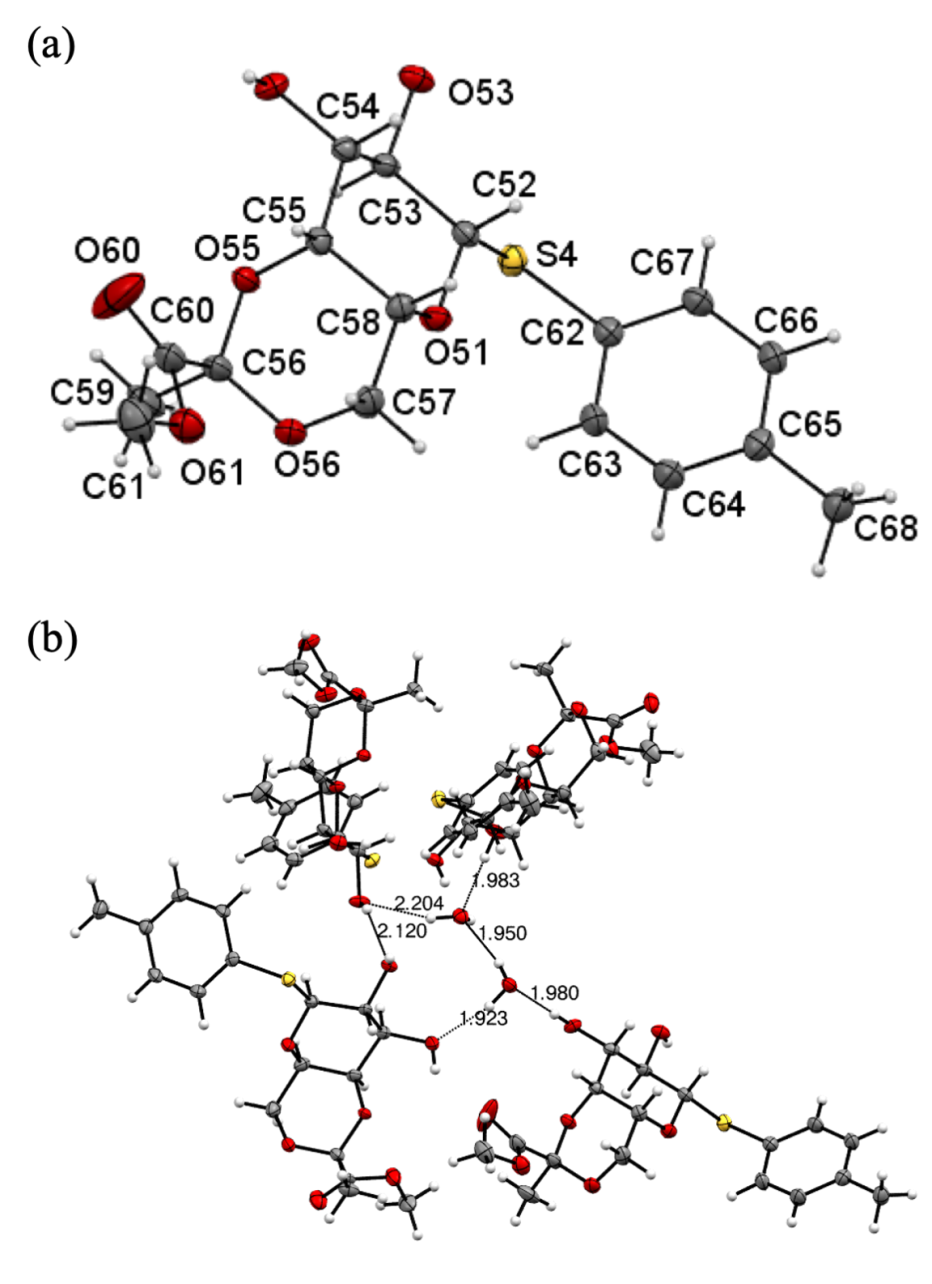
(a) Oak Ridge Thermal Ellipsoid Plot view of the X-ray crystal structure of pyruvylated galactose **6**. (b) Hydrogen-bonding in the crystal structure of pyruvylated galactose **6**.

To further confirm the diastereochemistry of the pyruvate ketals, we present the first X-ray structural evidence of **6**. The liquid compound **5** was treated with sodium methoxide in methanol to produce solid compound **6**. A single crystal of compound **6** was obtained through recrystallization using ethyl acetate and *n*-hexane. The X-ray structural data provided direct evidence supporting the *R*-configuration (Figure 2a). The material crystallized in the monoclinic space group *P*2_1_, with cell dimensions *a* = 16.1017(4) Å, *b* = 7.7608(2) Å, *c* = 28.8344(8) Å, and β = 93.3680(10)°. Additionally, the presence of lattice water greatly influenced the molecular packing. The hydrogen bond (H–O–H) lengths in this lattice water ranged from 1.923 to 2.204 Å (Figure 2b).

**Scheme 1:**
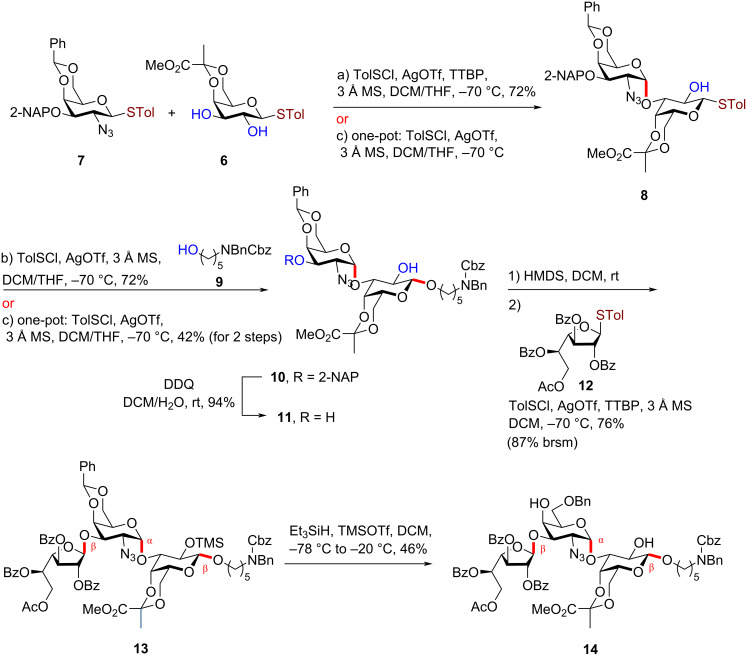
Synthesis of trisaccharide precursor **14**.

The pyruvylated galactose **6** was utilized to synthesize a pyruvate ketal-containing PS A1 precursor associated with the commensal anaerobic bacteria capsule (Scheme 1) [7–11]. The target structure was β-ᴅ-Galf-(1→3)-α-ᴅ-Gal-(1→3)-β-ᴅ-Gal. This molecule acts as a critical intermediate for synthesizing various O4-linked PS A1 derivatives, enabling the exploration of the practical applications of PSA1 while simultaneously enhancing our understanding of synthetic chemistry.

To simplify the synthetic process, glycosylation was facilitated by employing preactivation and the differences in acceptor reactivity. Using the *p*-toluenesulfinyl chloride/silver triflate (TolSCl/AgOTf) promoter system [30–39] in the mixture of dichloromethane/tetrahydrofuran (DCM/THF = 1:1) as the solvent, compound **7** [40] was preactivated and subsequently reacted with compound **6,** yielding the desired disaccharide **8** with a 72% yield and excellent regio- and stereoselectivity. Regarding the acceptor, compound **6** harbored two free alcohol groups at the C3 and C2 positions. Since the C3 alcohol group is less sterically hindered and exhibits better nucleophilicity, the glycosidic bond formed regioselectively at the C3 position. Additionally, THF was used as a solvent to enhance the α-selectivity of the reaction [41–43].

Next, while both compounds **8** and **9** can act as acceptors, compound **9,** a primary alcohol, exhibits greater nucleophilicity. Using the TolSCl/AgOTf promoter system at −70 °C, the β-linked disaccharide **10** was formed, giving 71% yield (α/β = 1:6). According to the RRV/Aka platform [44], compound **9** is a robust nucleophile, with an Aka value of 12 [45]. This high nucleophilicity favors a S_N_2-like mechanism, promoting β-selectivity [46]. Using the difference in acceptor reactivity and activation method, a one-pot synthesis approach was applied to successfully and quickly construct disaccharide **10**. In this protocol, donor **7** was first activated by the TolSCl/AgOTf promoter and reacted with **6** to form compound **8**. The reaction progress was monitored by thin-layer chromatography. Subsequently*,* in the same reaction mixture, in situ activation of disaccharide donor **8** and acceptor **9** using TolSCl/AgOTf reagent combination was introduced. This two-step, one-pot glycosylation gave a 42% yield of disaccharide **10**, maintaining good β-stereoselectivity at the galactosyl linkage (α/β = 1:5.8).

The 2-naphthylmethyl ether (Nap) protecting group was removed from disaccharide **10** using 2,3-dichloro-5,6-dicyano-1,4-benzoquinone (DDQ) in a DCM/H_2_O (9:1) mixture, giving a 94% yield of disaccharide acceptor **11**. To enhance solubility, the trimethylsilyl (TMS) group was selectively added at the less hindered 2-O position of the galactoside using 1.3 equivalents of bis(trimethylsilyl)amine (HMDS), concurrently leaving the 3-OH group at the non-reducing end available for glycosylation with the ᴅ-Gal*f* donor **12** [47]. After the in situ activation with TolSCl/AgOTf, product **13** was obtained with a 76% yield and exclusive β-selectivity due to the neighboring group effect [41,48–50]. Subsequently, treatment with triethylsilane (Et₃SiH) and trimethylsilyl trifluoromethanesulfonate (TMSOTf) led to the opening of the benzylidene ring and removal of the TMS group, yielding compound **14**, which can serve as an acceptor for O4-linked synthesis in preparing various PS A1 derivatives.

In our research, we carried out the synthesis of the PSA1 molecule as part of a broader effort to validate chemical synthesis methodologies. This work involved verifying our experimental techniques and understanding the underlying reaction mechanisms. Through this project, we aimed to explore the practical applications of PS A1 while simultaneously enhancing our understanding of synthetic chemistry. This endeavor reflects our commitment to advancing chemical knowledge and contributing to ongoing research in the field.

Next, in-depth ^1^H-^13^C HSQC NMR analysis was performed to further characterize the structure of **14** (see Supporting Information File 1). ^1^H NMR coupling constants revealed the presence of one α-glycosidic bond at C_1_′ (δ 5.49, *J* = 4.3 Hz) and two β-glycosidic bonds at C_1_ (δ 4.22, bs) and C_1_′′ (δ 5.56, *J* = 3.8 Hz). The linker at the reducing end adopts a rotamer configuration, leading to the broad singlet at δ 4.22 ppm. The ^13^C chemical shift of the methyl groups in galactose for all (*R*)-4,6-*O*-pyruvylated residues was determined to be between δ 25.5–25.7 ppm, with corresponding δ 1.50–1.65 ppm in ^1^H NMR. This NMR pattern aligns with existing literature and is corroborated by our X-ray analysis. High-resolution electrospray ionization mass spectrometry confirmed the molecular mass, showing spectral peaks aligning with the calculated mass ([M + Na]^+^ calcd for C_72_H_78_N_4_O_23_Na, 1389.4949; found, 1389.4943).

## Conclusion

In conclusion, we could successfully synthesize the pure *R*-isomer of pyruvated galactose, simultaneously avoiding anomeric epimerization. The crystal structure of pyruvylated galactose, potentially stabilized by hydrogen bonding, provided direct evidence confirming the diastereochemistry of the pyruvate ketals. The modified galactose moiety was then used for the synthesis of the trisaccharide PS A1 precursor via a one-pot glycosylation strategy, where the glycosylation product was efficiently formed based on differences in acceptor reactivity. The synthetic protocol was concise, yielding promising regio- and stereoselectivity. Detailed characterization using NMR and X-ray methods clarified the diastereoselective structure of the pyruvate ketals, enabling the successful synthesis of oligosaccharides containing pyruvate ketal groups.

## Supporting Information

File 1 Experimental procedures and NMR spectra.

## Data Availability

Data generated and analyzed during this study is available from the corresponding author upon reasonable request.
