# Supplementary material for: Structural analysis of stereoselective galactose pyruvylation toward the synthesis of bacterial capsular polysaccharides
Source: Beilstein J Org Chem. 2025 Aug 21;21:1671–7. doi: 10.3762/bjoc.21.131 (PMC12415908; doi:10.3762/bjoc.21.131)
Supplement: File 1 — Experimental procedures and NMR spectra. [file Beilstein_J_Org_Chem-21-1671-s001.pdf]

BEILSTEIN JOURNAL OF ORGANIC CHEMISTRY

Supporting Information

for

Structural analysis of stereoselective galactose pyruvylation toward the synthesis of bacterial capsular polysaccharides

Tsun-Yi Chiang, Mei-Huei Lin, Chun-Wei Chang, Jinq-Chyi Lee and Cheng-Chung Wang

Beilstein J. Org. Chem. **2025,** *21,* 1671–1677. doi:10.3762/bjoc.21.131

Experimental procedures and NMR spectra

License and Terms: This is a supporting information file under the terms of the Creative Commons Attribution License (https://creativecommons.org/licenses/by/4.0). Please note that the reuse, redistribution and reproduction in particular requires that the author(s) and source are credited and that individual graphics may be subject to special legal provisions.
The license is subject to the *Beilstein Journal of Organic Chemistry* terms and conditions: (https://www.beilstein-journals.org/bjoc/terms)

This PDF file includes:

1. **General procedure S2**

2. **Experimental procedure and characterization data S3**

3. **Crystal data and structure refinement for compound 7 .... S22**

4. **References S23**

5. **NMR spectra S24**

1. General procedure

All reactions were conducted in flame-dried glassware, under nitrogen atmosphere. All solvents were purified and dried from a safe purification system containing activated Al_2O_3. All reagents obtained from commercial sources were used without purification, unless otherwise mentioned. Flash column chromatography was carried out by Silica Gel Geduran® Si 60 (0.040–0.063 mm, E. Merck). TLC was performed on pre-coated aluminium sheets of Silica Gel 60 F254 (0.25 mm, E. Merck); detection was executed by spraying with a solution of $Ce(SO_4)_2$, $(NH_4)_2MoO_4$, and H_2SO_4 in water and subsequently heating on a hot plate. UV light for TLC analysis was UVGL-25 compact UV lamp (4 watt/ 254 nm), UVP. Optical rotations were measured with a JASCO P-2000 polarimeter at 25–35 °C, and using a 50 mm cell at 589 nm (Na). High performance liquid chromatography (HPLC) reactions were carried out by Agilent Technologies 1200 Series. ^{1}H, ^{13}C, DEPT, 1D-TOCSY and ^{1}H-^{13}C HSQC spectra were recorded by Bruker AV400, AVIII500 and N600 MHz instruments. Chemical shifts are in ppm from Me_4Si, generated from the $CDCl_3$ lock signal at δ 7.26. Multiplicities are reports by using the following abbreviations: s = singlet, d = doublet, t = triplet, m = multiplet, br = broad, ABq = AB quartets, dd = doublet of doublets, td = triplet of doublets; J = coupling constant values in Hertz. IR spectra were taken by a Bruker alpha FT-IR spectrometer using ZnSe plates. Mass spectra were analyzed by a Waters Premier XE instrument with ESI source.

2. Experimental procedure and characterization data

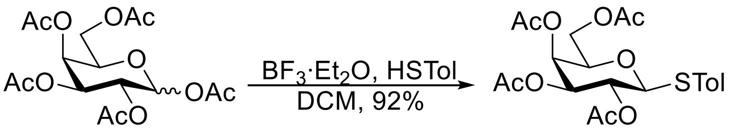

15 **16**

***p*-Tolyl 2,3,4,6-tetra-*O*-acetyl-1-thio-β-D-galactopyranoside** (**16**).[1] ^{1}H NMR (500 MHz, $CDCl_3$) δ 7.41 (d, *J* = 8.1 Hz, 2H, Ar-H), 7.12 (d, *J* = 8.1 Hz, 2H, Ar-H), 5.40 (dd, *J* = 3.3, 0.9 Hz, 1H, H-4), 5.22 (t, *J* = 10.1 Hz, 1H, H-2), 5.03 (dd, *J* = 10.1, 3.3 Hz, 1H, H-3), 4.64 (d, *J* = 10.1 Hz, 1H, H-1), 4.19, 4.11 (ABq, *J* = 11.3 Hz, 1H, H-6a), 4.18, 4.10 (ABq, *J* = 11.3 Hz, 1H, H-6b), 3.91 (td, *J* = 7.0, 0.9 Hz, 1H, H-5), 2.34 (s, 3H, CH_3), 2.11 (s, 3H, OAc), 2.10 (s, 3H, OAc), 2.04 (s, 3H, OAc), 1.97 (s, 3H, OAc); ^{13}C NMR (125 MHz, $CDCl_3$) δ 170.4 (C), 170.2 (C), 170.0 (C), 138.5 (C), 133.1 (CH, Ar-C), 129.6 (CH, Ar-C), 128.6 (C), 87.0 (CH, C-1), 74.3 (CH, C-5), 72.0 (CH), 67.3 (CH, C-2), 67.2 (CH, C-4, C-3), 61.6 (CH_2, C-6), 21.1 (CH_3, STol), 20.9 (CH_3, OAc), 20.7 (CH_3, OAc), 20.62 (CH_3, OAc), 20.57 (CH_3, OAc).

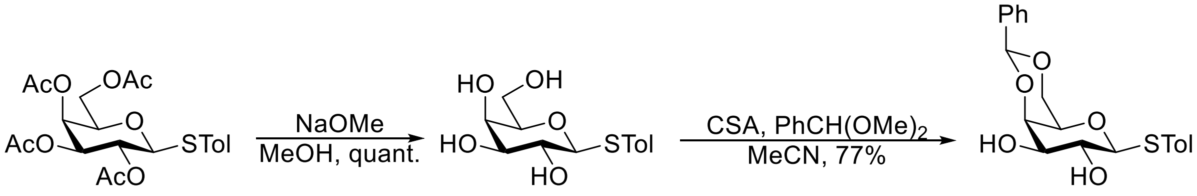

16 **17** **18**

***p*-Tolyl 4,6-*O*-benzylidene-1-thio-β-D-galactopyranoside** (**18**). To a solution of compound **16**[1] (19.0 g, 41.83 mmol) and NaOMe (1.8 g, 33.46 mmol) in MeOH (200 mL) at room temperature. The solution was stirred for 1 h and was neutralized with Amberlite 120 (H^+). The resin was filtered off and washed with MeOH (200 mL). The filtrate was concentrated in vacuo to afford the compound **17**. The deacetylated compound **17** was dissolved in MeCN (150 mL) under nitrogen atmosphere. $PhCH(OMe)_2$ (16.6 mL, 110.88 mmol) and CSA (4.2 g,

17.76 mmol) were added and the mixture was stirred at room temperature for 12 h. The reaction mixture was quenched by Et_3N and then purified by flash column chromatography (*n*-hexane/EtOAc 1:3) to give product **18** (12.8 g, 77%). $[\alpha]^{33}_{D}$ -36.7 (*c* 1.3, CH_2Cl_2); IR (CH_2Cl_2) ν 3404, 2919, 2850, 1098, 1072, 1037, 994, 811 cm^{-1}; ^1H NMR (500 MHz, $CDCl_3$) δ 7.58 (d, *J* = 8.1 Hz, 2H, Ar-H), 7.40-7.34 (m, 5H, Ph-H), 7.12 (d, *J* = 8.1 Hz, 2H, Ar-H), 5.51 (s, 1H, CHPh), .4.46 (d, *J* = 9.3 Hz, 1H, H-1), 4.38 (dd, *J* = 12.5, 1.1 Hz, 1H, H-6eq), 4.22 (d, *J* = 3.5 Hz, 1H, H-4), 4.04 (dd, *J* = 12.5, 1.1 Hz, 1H, H-6ax), 3.70 (td, *J* = 9.3, 3.5 Hz, 1H, H-3), 3.64 (td, *J* = 9.3, 1.6 Hz, 1H, H-2), 3.56 (d, *J* = 1.1 Hz, 1H, H-5), 2.50 (d, *J* = 1.65 Hz, 1H, OH), 2.36 (s, 3H, CH_3); ^{13}C NMR (125 MHz, $CDCl_3$) δ 138.6 (C), 137.6 (C), 134.3 (CH, Ar-C), 129.7 (CH, Ar-C), 129.4 (CH, Ph-C), 128.2 (CH, Ph-C), 126.6 (CH, Ph-C), 101.4 (CH, CHPh), 87.0 (CH, C-1), 75.3 (CH, C-4), 73.7 (CH, C-3), 70.0 (CH, C-5), 69.3 (CH_2, C-6), 68.7 (CH, C-2), 21.2 (CH_3, STol); HRMS (ESI) calcd for $C_{20}H_{22}O_5SNa$ $[M + Na]^+$ 397.1080 found 397.1080.

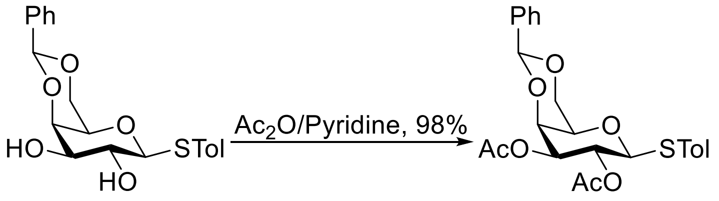

18 **19**

***p*-Tolyl 2,3-di-*O*-acetyl-4,6-*O*-benzylidene-1-thio-β-D-galactopyranoside** (**19**). The compound **18** (11.8 g, 31.46 mmol) was reacted in a mixture of pyridine/Ac_2O (210 mL/70 mL). The reaction stirred at room temperature for overnight. The mixture was extracted with DCM (200 mL × 3) and $CuSO_4$ (200 mL). The organic layers were combined, dried over anhydrous $MgSO_4$, filtered and evaporated under reduced pressure. The crude product was purified by flash column chromatography EtOAc/Hexane 1/2 on silica gel to give product **19** (14.2 g, 98%). $[\alpha]^{35}_{D}$ -4.6 (*c* 1.7, CH_2Cl_2); IR (CH_2Cl_2) ν 2918, 2849, 1752, 1371, 1239, 1172, 1096, 1057, 1022, 1001, 922, 810, 699 cm^{-1}; ^1H NMR (500 MHz, $CDCl_3$) δ 7.50 (d, *J* = 8.1 Hz,

2H, Ar-H), 7.36 (m, 5H, Ph-H), 7.07 (d, J = 8.1 Hz, 2H, Ar-H), 5.45 (s, 1H, CHPh), 5.29 (t, J = 9.8 Hz, 1H, H-2), 4.98 (dd, J = 9.8, 3.5 Hz, 1H, H-3), 4.65 (d, J = 9.8 Hz, 1H, H-1), 4.36 (dd, J = 12.4, 1.6 Hz, 1H, H-6eq), 4.35 (d, J = 1.1 Hz, 1H, H-4), 4.01 (dd, J = 12.4, 1.6 Hz, 1H, H-6ax), 3.57 (d, J = 1.1 Hz, 1H, H-5), 2.34 (s, 3H, CH_3), 2.09 (s, 3H, OAc), 2.02 (s, 3H, OAc); ^{13}C NMR (125 MHz, $CDCl_3$) δ 170.7 (C), 169.1 (C), 138.3 (C), 137.5 (C), 134.3 (CH, Ar-C), 129.5 (CH, Ar-C), 129.1 (CH, Ph-C), 128.1 (CH, Ph-C), 127.2 (C), 126.6 (CH, Ph-C), 101.1 (CH, CHPh), 85.2 (CH, C-1), 73.5 (CH, C-4), 73.2 (CH, C-3), 69.7 (CH, C-5), 69.1 (CH_2, C-6), 66.8 (CH, C-2), 21.2 (CH_3, STol), 20.9 (CH_3, OAc), 20.9 (CH_3, OAc); HRMS (ESI) calcd for $C_{24}H_{26}O_7SNa$ $[M + Na]^+$ 481.1291, found 481.1285.

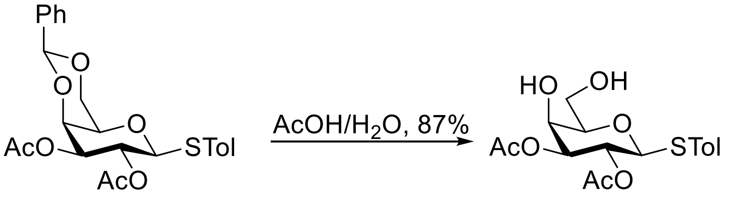

19 4

***p*-Tolyl 2,3-di-*O*-acetyl-1-thio-β-D-galactopyranoside** (**4**). The compound **19** (13.9 g, 30.38 mmol) was reacted in a mixture of AcOH/H_2O (250 mL/125 mL). And the reaction stirred at 60 °C for overnight, The mixture was extracted with DCM (200 mL × 3) and $NaHCO_3$ (200 mL). The organic layers were combined, dried over anhydrous $MgSO_4$, filtered and evaporated under reduced pressure. The crude product was purified by flash column chromatography EtOAc/hexane 1:1 on silica gel to give product **4** (9.8 g, 87%). $[\alpha]^{34}_D$ +5.1 (*c* 1.0, CH_2Cl_2); IR (CH_2Cl_2) ν 3462, 2920, 2850, 1746, 1372, 1229, 1055, 809 cm^{-1}; ^1H NMR (500 MHz, $CDCl_3$) δ 7.39 (d, J = 8.2 Hz, 2H, Ar-H), 7.12 (d, J = 8.2 Hz, 2H, Ar-H), 5.28 (t, J = 10.0 Hz, 1H, H-2), 4.97 (dd, J = 10.0, 3.2 Hz, 1H, H-3), 4.66 (d, J = 10.0 Hz, 1H, H-1), 4.17 (d, J = 1.3 Hz, 1H, H-4), 3.94 (dd, J = 12.0, 5.8 Hz, 1H, H-6a), 3.86 (dd, J = 12.0, 3.5 Hz, 1H, H-6b), 3.62 (td, J = 5.8, 1.3 Hz, 1H, H-5), 2.33 (s, 3H, CH_3), 2.09 (s, 3H, OAc), 2.08 (s, 3H, OAc); ^{13}C NMR (125 MHz, $CDCl_3$) δ 170.2 (C), 169.6 (C), 138.5 (C), 133.1 (CH, Ar-C), 129.8

(CH, Ar-C), 128.3 (C), 86.6 (CH, C-1), 77.8 (CH, C-5), 74.4 (CH, C-3), 68.5 (CH, C-4), 67.6 (CH, C-2), 62.8 (CH_2, C-6), 21.1 (CH_3, STol), 21.0 (CH_3, OAc, OAc); HRMS (ESI) calcd for $C_{17}H_{22}O_7SNa$ $[M + Na]^+$ 393.0978, found 393.0970.

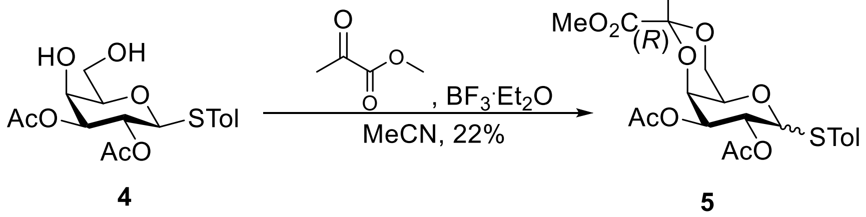

***p*-Tolyl 2,3-di-*O*-acetyl-4,6-*O*-[1-(*R*)-(methyloxycarbonyl)-ethylidene]-1-thio-D-galactopyranoside** (**5**). The compound **4** (144 mg, 0.39 mmol) was dissolved in MeCN (1.4 mL). And then injected methyl pyruvate (72 μL, 0.78 mmol) and $BF_3{\cdot}Et_2O$ (100 μL, 0.78 mmol) into the solution. The reaction stirred at room temperature for 18.5 h. The resulting solution was concentrated under reduced pressure. The mixture was extracted with DCM (5 mL × 3) and $NaHCO_3$ (5 mL). The organic layers were combined, dried over anhydrous $MgSO_4$, filtered and evaporated under reduced pressure. The crude product was purified by flash column chromatography EtOAc/hexane 1:2 on silica gel to give product **5** (41 mg, 22%, α/β = 1/1.6). β-anomer: $[\alpha]^{32}_D$ -59.1 (*c* 0.8, CH_2Cl_2); IR (CH_2Cl_2) ν 2962, 2920, 1742, 1492, 1123, 1066, 1021, 988, 962, 938, 868, 836, 809, 768, 645 cm^{-1}; ^1H NMR (500 MHz, $CDCl_3$) δ 7.47 (d, *J* = 8.1 Hz, 2H, Ar-H), 7.12 (d, *J* = 8.1 Hz, 2H, Ar-H), 5.27 (t, *J* = 9.9 Hz, 1H, H-2), 4.83 (dd, *J* = 9.9, 3.4 Hz, 1H, H-3), 4.59 (d, *J* = 9.9 Hz, 1H, H-1), 4.31 (d, *J* = 3.4 Hz, 1H, H-4), 4.11 (dd, *J* = 12.8, 1.0 Hz, 1H, H-6eq), 3.92 (dd, *J* = 12.8, 1.0 Hz, 1H, H-6ax), 3.75 (s, 3H, OCH_3), 3.44 (d, *J* = 1.0 Hz, 1H, H-5), 2.33 (s, 3H, CH_3), 2.10 (s, 3H, OAc), 2.07 (s, 3H, OAc), 1.50 (s, 3H, CH_3); ^{13}C NMR (125 MHz, $CDCl_3$) δ 171.2 (C), 170.6 (C), 170.2 (C), 169.2 (C), 138.4 (C), 133.9 (CH, Ar-C), 129.5 (CH, Ar-C), 127.7 (C), 85.6 (CH, C-1), 73.1 (CH, C-3), 68.8 (CH, C-4), 68.7 (CH, C-5), 66.4 (CH, C-2), 65.3 (CH_2, C-6), 52.5 (CH_3, OCH_3), 25.5 (CH_3, CCH_3), 21.2 (CH_3, STol), 20.9 (CH_3, OAc), 20.7 (CH_3, OAc); HRMS (ESI) calcd for $C_{21}H_{26}O_9SNa$ [M

+ Na]+ 477.1201, found 477.1191. α-anomer: $[\alpha]^{29}_D$ +177.6 (*c* 0.3, CH_2Cl_2); IR (CH_2Cl_2) ν 2945, 2921, 1744, 1493, 1221, 1153, 1124, 1099, 1086, 1062, 977, 919, 808, 753 cm^{-1}; ^1H NMR (500 MHz, $CDCl_3$) δ 7.47 (d, *J* = 8.0 Hz, 2H, Ar-H), 7.08 (d, *J* = 8.0 Hz, 2H, Ar-H), 5.99 (d, *J* = 5.5 Hz, 1H, H-1), 5.50 (dd, *J* = 10.9, 5.5 Hz, 1H, H-2), 5.10 (dd, *J* = 10.9, 3.5 Hz, 1H, H-3), 4.44 (d, *J* = 3.4 Hz, 1H, H-5), 4.13 (s, 1H, H-4), 3.98 (dd, *J* = 13.1, 1.5 Hz, 1H, H-6eq), 3.91 (dd, *J* = 13.1, 1.5 Hz, 1H, H-6ax), 3.77 (s, 3H, OCH_3), 2.30 (s, 3H, CH_3), 2.13 (s, 3H, OAc), 2.09 (s, 3H, OAc), 1.57 (s, 3H, CH_3); ^{13}C NMR (125 MHz, $CDCl_3$) δ 170.3 (C), 170.1 (C), 170.0 (C), 137.5 (C), 131.4 (CH, Ar-C), 129.8 (CH, Ar-C), 129.4 (CH, Ar-C), 85.9 (CH, C-1), 69.1 (CH, C-5), 69.0 (CH, C-3), 67.5 (CH, C-2), 65.2 (CH_2, C-6), 62.1 (CH, C-4), 52.5 (CH_3, OCH_3), 25.7 (CH_3, CCH_3), 21.0 (CH_3, STol), 20.80 (CH_3, OAc), 20.75 (CH_3, OAc); HRMS (ESI) calcd for $C_{21}H_{26}O_9SNa$ $[M + Na]^+$ 477.1190, found 477.1198.

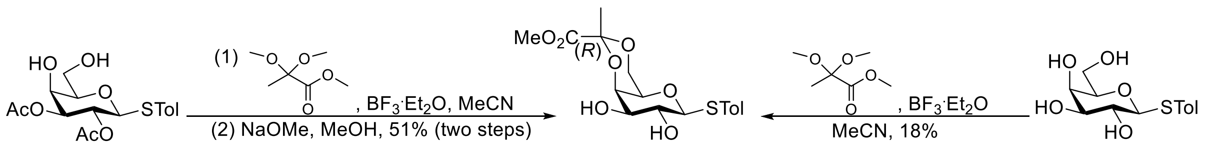

4 6 17

***p*-Tolyl 4,6-*O*-[1-(*R*)-(methyloxycarbonyl)-ethylidene]-1-thio-β-D-galactopyranoside** (**6**). Method from compound **4**: compound **4** (3.2 g, 8.52 mmol) was dissolved in MeCN (30 mL). And then injected methyl 2,2-dimethoxypropionate (2.4 mL, 17.05 mmol) and $BF_3·Et_2O$ (2.1 mL, 17.05 mmol) into the solution. The reaction stirred at room temperature for 2 h. After that, the solution was quenched by triethylamine (3 mL) in ice pot. Then, the resulting solution was concentrated under reduced pressure. The mixture was extracted with DCM (30 mL × 3) and $NaHCO_3$ (30 mL). The organic layers were combined, dried over anhydrous $MgSO_4$, filtered and evaporated under reduced pressure. And then went to do deacetylation directly. To a solution of mixture (2.9 g) and NaOMe (278 mg, 5.14 mmol) in MeOH (30 mL) at room temperature. The solution was stirred for 1 h and was neutralized with Amberlite 120 (H^+). The resin was filtered off and washed with MeOH (30 mL). The crude product was purified by flash

column chromatography EtOAc/hexane 2.1 on silica gel to give product **6** (1.62 g, overall yield: 51%). Method from compound **17**: compound **17** (2.5 g, 8.73 mmol) was dissolved in MeCN (20 mL). And then injected methyl 2,2-dimethoxypropionate (2.4 mL, 17.46 mmol) and $BF_3 \cdot Et_2O$ (2.2 mL, 17.46 mmol) into the solution. The reaction stirred at room temperature for 18 h. After that, the solution was quenched by triethylamine (3 mL) in ice pot. Then, the resulting solution was concentrated under reduced pressure. The mixture was extracted with DCM (30 mL × 3) and $NaHCO_3$ (30 mL). The organic layers were combined, dried over anhydrous $MgSO_4$, filtered and evaporated under reduced pressure. The crude product was purified by flash column chromatography EtOAc/hexane 2:1 on silica gel to give product **6** (584 mg, 18%). $[\alpha]^{34}_{D}$ -40.1 (*c* 1.7, CH_2Cl_2); IR (CH_2Cl_2) ν 3447, 2922, 2852, 1743, 1276, 1202, 1173, 1127, 1103, 1086, 1063, 979, 877, 827, 812 cm^{-1}; ^1H NMR (400 MHz, $CDCl_3$) δ 7.54 (d, *J* = 8.1 Hz, 2H, Ar-H), 7.13 (d, *J* = 8.1 Hz, 2H, Ar-H), 4.42 (d, *J* = 9.2 Hz, 1H, H-1), 4.14 (s, 1H, H-4), 4.11 (d, *J* = 1.0 Hz, 1H, H-6eq), 3.96 (dd, *J* = 12.8, 1.0 Hz, 1H, H-6ax), 3.81 (s, 3H, OCH_3), 3.64-3.62 (m, 2H, H-2, H-3), 3.43 (d, *J* = 1.0 Hz, 1H, H-5), 2.51 (s, 1H, OH), 2.34 (s, 3H, CH_3), 1.53 (s, 3H, CH_3); ^{13}C NMR (100 MHz, $CDCl_3$) δ 170.2 (C), 138.5 (C), 133.8 (CH, Ar-C), 129.7 (CH, Ar-C), 127.3 (C), 98.5 (C), 87.5 (CH, C-1), 73.6 (CH, C-3), 71.0 (CH, C-4), 69.1 (CH, C-5), 68.7 (CH, C-2), 65.4 (CH_2, C-6), 52.7 (CH_3, OCH_3), 25.7 (CH_3, CCH_3), 21.2 (CH_3, STol); HRMS (ESI) calcd for $C_{17}H_{22}O_7SNa$ $[M + Na]^+$ 393.0978, found 393.0971.

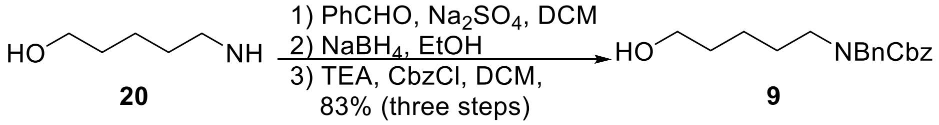

20 **9**

***N*-(Benzyl)-benzyloxycarbonyl-5-aminopentanol** (**9**). The compound **20** (1.0 g, 9.69 mmol) and Na_2SO_4 (13.7 g, 96.03 mmol) was dissolved in dry DCM (10 mL). And then injected PhCHO (1.1 mL, 10.67 mmol) into the solution. The reaction stirred at room temperature for 19.5 h. The resulting solution was concentrated under reduced pressure. The mixture was added

$NaBH_4$ (550 mg, 14.55 mmol) in EtOH (5 mL). The reaction stirred in ice bath for 2 h. The mixture was quenched by H_2O and the resulting solution was concentrated under reduced pressure. And then extracted with EtOAc (10 mL × 3) and $NaHCO_3$ (10 mL). The organic layers were combined, dried over anhydrous $MgSO_4$, filtered and evaporated under reduced pressure. And then the mixture dissolved in dry DCM (10 mL). To a solution of mixture injected TEA (2.0 mL, 14.55 mmol) in ice bath for 5 min. And then injected CbzCl (2.2 mL, 14.55 mmol) in the solution. The solution was stirred for 1 h and was extracted with DCM (10 mL × 3) and $NaHCO_3$ (10 mL). The organic layers were combined, dried over anhydrous $MgSO_4$, filtered and evaporated under reduced pressure. The crude product was purified by flash column chromatography EtOAc/hexane 1:2 on silica gel to give product **9** (2.64 g, overall yield: 83%). $[\alpha]^{34}_{D}$ -0.8 (*c* 2.9, CH_2Cl_2); IR (CH_2Cl_2) ν 3443, 2931, 1682, 1473, 1454, 1422, 1365, 1304, 1229, 1159, 1124, 1071, 768, 732, 697 cm^{-1}; ^1H NMR (400 MHz, $CDCl_3$) δ 7.37-7.18 (m, 10H, Ph-H), 5.18 (d, J = 9.9 Hz, 2H, CH_2Ph), 4.50 (s, 2H, CH_2Ph), 3.60-3.53 (m, 2H, CH_2), 3.28-3.21 (m, 2H, CH_2), 1.56-1.51 (m, 4H, CH_2, CH_2), 1.34-1.24 (m, 2H, CH_2); ^{13}C NMR (100 MHz, $CDCl_3$) δ 156.7 (C), 156.3 (C), 137.9 (C), 136.7 (C), 128.5 (CH), 128.4 (CH), 127.9 (CH), 127.8 (CH), 127.3 (CH), 67.2 (CH_2), 62.6 (CH_2), 50.5 (CH_2), 50.2 (CH_2), 47.0 (CH_2), 46.1 (CH_2), 32.2 (CH_2), 27.8 (CH_2), 27.4 (CH_2), 22.9 (CH_2); HRMS (ESI) calcd for $C_{20}H_{25}NO_3Na$ $[M + Na]^+$ 350.1727, found 350.1720.

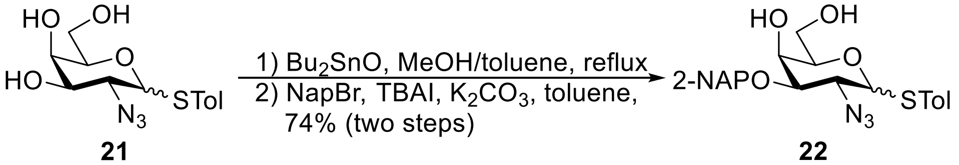

***p*-Tolyl 2-azido-2-deoxy-3-*O*-naphthyl-1-thio-D-galactopyranoside** (**22**). To a solution of compound **21**[2,3] (3.16 g, 10.15 mmol) in dry toluene/MeOH (140 mL/140 mL) was added Bu_2SnO (3.87 g, 15.23 mmol). The reaction mixture was stirred under reflux condition at 80 °C for 5 h. The resulting solution was concentrated under reduced pressure then injected dry

toluene into mixture. And then TBAI (750 mg, 2.03 mmol), K_2CO_3 (2.10 g, 15.23 mmol) and 2-(Bromomethyl) naphthalene (3.54 g, 15.23 mmol) was added. The reaction stirred under reflux condition at 80 ºC for overnight. The mixture was extracted with EtOAc (140 mL × 3) and water (140 mL). The organic layers were combined, dried over anhydrous $MgSO_4$, filtered and evaporated under reduced pressure. The crude product was purified by flash column chromatography EtOAc/hexane 1:2 on silica gel to give the product **22** (3.39 g, 74%, α/β = 1/1). α-anomer: $[\alpha]^{32}_D$ +154.3 (*c* 1.5, CH_2Cl_2); IR (CH_2Cl_2) ν 3443, 3053, 2923, 2108, 1509, 1300, 1268, 1210, 1122, 1069, 981, 951, 858, 812, 753 cm^{-1}; ^1H NMR (600 MHz, $CDCl_3$) δ 7.89-7.85 (m, 4H, Ar-H), 7.54-7.50 (m, 3H, Ar-H), 7.38 (d, *J* = 8.1 Hz, 2H, Ar-H), 7.12 (d, *J* = 8.1 Hz, 2H, Ar-H), 5.58 (d, *J* = 5.5 Hz, 1H, H-1), 4.93, 4.87 (ABq, *J* = 11.6 Hz, 2H, CH_2NAP), 4.34 (t, *J* = 4.9 Hz, 1H, H-5), 4.30 (dd, *J* = 10.4, 5.5 Hz, 1H, H-2), 4.16 (d, *J* = 1.6 Hz, 1H, H-4), 3.90 (dd, *J* = 11.6, 4.9 Hz, 1H, H-6a), 3.82-3.75 (m, 2H, H-3, H-6b), 2.78 (s, 1H, OH), 2.33 (s, 3H, CH_3); ^{13}C NMR (150 MHz, $CDCl_3$) δ 138.2 (C), 134.3 (C), 133.2 (C), 133.1 (CH, Ar-C), 129.9 (CH, Ar-C), 129.0 (C), 128.7 (CH, Ar-C), 128.0 (CH, Ar-C), 127.8 (CH, Ar-C), 127.1 (CH, Ar-C), 126.4 (CH, Ar-C), 126.3 (CH, Ar-C), 125.7 (CH, Ar-C), 87.5 (CH, C-1), 77.6 (CH, C-3), 72.4 (CH_2, CH_2NAP), 70.4 (CH, C-5), 67.7 (CH, C-4), 62.9 (CH_2, C-6), 59.7 (CH, C-2), 21.1 (CH_3, STol); HRMS (ESI) calcd for $C_{24}H_{25}N_3O_4SNa$ $[M + Na]^+$ 474.1458, found 474.1449. β-anomer: $[\alpha]^{31}_D$ -49.8 (*c* 0.8, CH_2Cl_2); IR (CH_2Cl_2) ν 3423, 2922, 2112, 1492, 1366, 1274, 1143, 1124, 1084, 1041, 860, 811, 753 cm^{-1}; ^1H NMR (500 MHz, $CDCl_3$) δ 7.87-7.79 (m, 4H, Ar-H), 7.50-7.47 (m, 5H, Ar-H), 7.13 (d, *J* = 7.9 Hz, 2H, Ar-H), 4.87, 4.84 (ABq, *J* = 11.8 Hz, 2H, CH_2NAP), 4.33 (d, *J* = 10.1 Hz, 1H, H-1), 4.04 (d, *J* = 2.4 Hz, 1H, H-4), 3.95 (dd, *J* = 11.7, 6.5 Hz,1H, H-6a), 3.81-3.79 (br, 1H, H-6b), 3.62 (t, *J* = 10.1 Hz, 1H, H-2), 3.46-3.43 (m, 2H, H-3, H-5), 2.53 (s, 1H, OH), 2.33 (s, 3H, CH_3); ^{13}C NMR (125 MHz, $CDCl_3$) δ 138.7 (C), 134.2 (C), 133.9 (CH, Ar-C), 133.2 (C), 133.1 (C), 129.8 (CH, Ar-C), 128.6 (CH, Ar-C), 127.9 (CH, Ar-C), 127.7 (CH, Ar-C), 127.3 (C), 127.1 (CH, Ar-C), 126.4 (CH, Ar-C), 126.3 (CH, Ar-C), 125.7 (CH, Ar-C), 86.3 (CH, C-1), 80.8 (CH, C-3), 78.0 (CH, C-5), 72.2 (CH_2,

CH_2NAP), 66.2 (CH, C-4), 62.8 (CH_2, C-6), 61.0 (CH, C-2), 21.2 (CH_3, STol); HRMS (ESI) calcd for $C_{24}H_{25}N_3O_4SNa$ $[M + Na]^+$ 474.1458, found 474.1450.

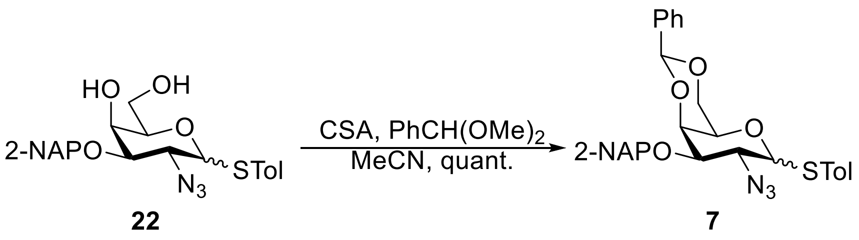

p-Tolyl 2-azido-4,6-*O*-benzylidene-2-deoxy-3-*O*-naphthyl-1-thio-D-galactopyranoside (**7**). The compound **22** was dissolved in MeCN (5 mL) under nitrogen atmosphere. $PhCH(OMe)_2$ (417 µL, 2.78 mmol) and CSA (156 mg, 0.44 mmol) were added and the mixture was stirred at room temperature for overnight. The reaction mixture was quenched by Et_3N and then purified by flash column chromatography (*n*-hexane/EtOAc 1:3) to give product **7** (986 mg, quant., α/β = 1/2). α-anomer: $[\alpha]^{32}_D$ +183.1 (*c* 0.5, CH_2Cl_2); IR (CH_2Cl_2) ν 3054, 2910, 2860, 2110, 1492, 1301, 1252, 1161, 1124, 1101, 1077, 1027, 744, 715 cm^{-1}; ^1H NMR (500 MHz, $CDCl_3$) δ 7.88-7.80 (m, 4H, Ar-H), 7.58-7.47 (m, 5H, Ar-H), 7.40-7.34 (m, 5H, Ar-H, Ph-H), 7.10 (d, *J* = 8.0 Hz, 2H, Ar-H), 5.70 (d, *J* = 5.3 Hz, 1H, H-1), 5.48 (s, 1H, CHPh), 4.97, 4.91 (ABq, *J* = 12.3 Hz, 2H, CH_2NAP), 4.51 (dd, *J* = 10.6, 5.3 Hz, 1H, H-2), 4.25 (dd, *J* = 3.4, 1.0 Hz, 1H, H-4), 4.18 (dd, *J* = 12.5, 1.5 Hz, 1H, H-6eq), 4.12 (d, *J* = 1.0 Hz, 1H, H-5), 4.03 (dd, *J* = 12.5, 1.5 Hz, 1H, H-6ax), 3.89 (dd, *J* = 10.6, 3.4 Hz, 1H, H-3), 2.32 (s, 3H, CH_3); ^{13}C NMR (125 MHz, $CDCl_3$) δ 137.6 (C), 137.5 (C), 135.2 (C), 133.2 (C), 133.1 (C), 131.6 (CH, Ph-C), 129.9 (CH, Ph-C), 129.1 (CH, Ph-C),128.3 (CH, Ar-C), 128.2 (CH, Ar-C), 127.9 (CH, Ar-C), 127.7 (CH, Ar-C), 126.5 (CH, Ar-C), 126.2 (CH, Ar-C), 126.1 (CH, Ar-C), 125.6 (CH, Ar-C), 101.0 (CH, CHPh), 87.9 (CH, C-1), 76.5 (CH, C-3), 73.0 (CH, C-4), 71.6 (CH_2, CH_2NAP), 69.3 (CH_2, C-6), 63.7 (CH, C-5), 59.4 (CH, C-2), 21.1 (CH_3, STol); HRMS (ESI) calcd for $C_{31}H_{29}N_3O_4SNa$ $[M + Na]^+$ 562.1771, found 562.1762. β-anomer: $[\alpha]^{31}_D$ -37.5 (*c* 2.1, CH_2Cl_2); IR (CH_2Cl_2) ν 3055, 2864, 2110, 1492, 1363, 1341, 1282, 1245, 1168,

1099, 1078, 1050, 1019, 985, 810, 755, 733, 698 cm^{-1}; ^{1}H NMR (500 MHz, $CDCl_3$) δ 7.85-7.76 (m, 4H, Ar-H), 7.62 (d, *J* = 8.0 Hz, 2H, Ar-H), 7.51-7.37 (m, 8H, Ar-H), 7.06 (d, *J* = 8.0 Hz, 2H, Ar-H), 5.42 (s, 1H, CHPh), 4.88, 4.85 (ABq, *J* = 12.6 Hz, 2H, CH_2NAP), 4.35-4.31 (m, 2H, H-6eq, H-1), 4.07 (d, *J* = 3.3 Hz, 1H, H-4), 3.93 (dd, *J* = 12.3, 1.5 Hz, 1H, H-6ax), 3.77 (t, *J* = 9.9 Hz, 1H, H-2), 3.48 (dd, *J* = 9.9, 3.3 Hz, 1H, H-3), 3.34 (d, *J* = 1.5 Hz, 1H, H-5), 2.34 (s, 3H, CH_3); ^{13}C NMR (125 MHz, $CDCl_3$) δ 138.7 (C), 137.7 (C), 135.1 (C), 134.9 (CH, Ar-C), 133.2 (C), 133.1 (C), 129.8 (CH, Ar-C), 129.2 (CH, Ar-C), 128.4 (CH, Ar-C), 128.2 (CH, Ar-C), 127.9 (CH, Ar-C), 127.8 (CH, Ar-C), 126.74 (CH, Ar-C), 126.65 (CH, Ar-C), 126.3 (CH, Ar-C), 126.14 (CH, Ar-C), 126.10 (C), 125.7 (CH, Ar-C), 101.3 (CH, CHPh), 85.2 (CH, C-1), 79.6 (CH, C-3), 72.3 (CH, C-4), 71.9 (CH_2, CH_2NAP), 69.8 (CH, C-5), 69.4 (CH_2, C-6), 59.8 (CH, C-2), 21.3 (CH_3, STol); HRMS (ESI) calcd for $C_{31}H_{29}N_3O_4SNa$ [M + Na]$^+$ 562.1771, found 562.1766.

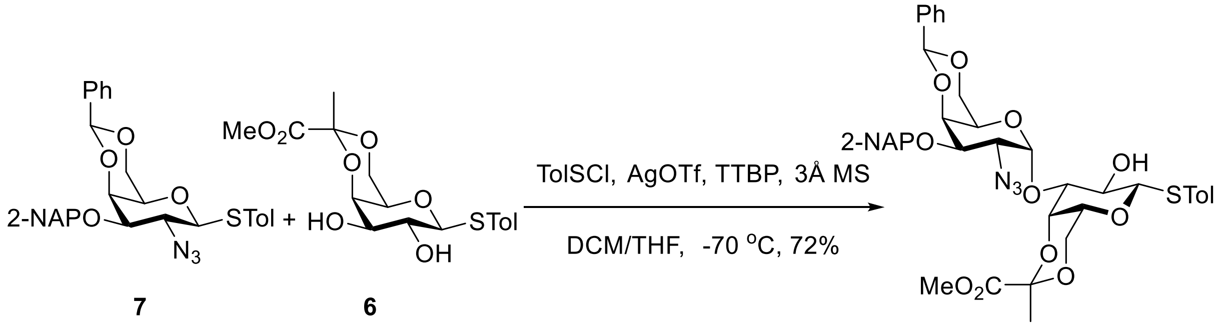

7 **6** **8**

***p*-Tolyl 4,6-*O*-[1-(*R*)-(methyloxycarbonyl)-ethylidene]-3-*O*-(2-azido-4,6-*O*-benzylidene-2-deoxy-3-*O*-naphthyl-α-D-galactopyranosyl)-1-thio-β-D-galactopyranoside (8)**. To a suspension of the thioglycoside donor **7** (76 mg, 0.14 mmol), molecular sieves (3 Å, 50 mg) and AgOTf (36 mg, 0.14 mmol) in dry DCM (500 μL) was stirred at −70 °C individually under nitrogen atmosphere for 1 h. TolSCl (22 μL, 0.14 mmol) was added into the reaction mixture at −70 °C and stirred for 30 mins at same temperature. The solution of Acceptor **6** (33 mg, 0.09 mmol) and TTBP (36 mg, 0.14 mmol) in dry THF (500 μL) was added into the

reaction mixture, and stirred at temperature −70 °C for 1.5 h judged by TLC. The solution was filtered through celite and washed with EtOAc. The filtrate was evaporated in vacuo to furnish the crude product, which was purified by flash chromatography to give the product **8** (best yield: 51 mg, 72%, α-only). $[\alpha]^{33}_{D}$ +76.5 (*c* 1.7, CH_2Cl_2); IR (CH_2Cl_2) ν 3479, 2922, 2855, 2111, 1743, 1493, 1453, 1401, 1366, 1337, 1271, 1202, 1176, 1125, 1098, 1069, 1046, 997, 982, 957, 876, 812, 753, 735, 700 cm^{-1}; ^1H NMR (500 MHz, $CDCl_3$) δ 7.86-7.76 (m, 4H, Ar-H), 7.55-7.45 (m, 7H, Ar-H), 7.39-7.33 (m, 3H, Ph-H), 7.13 (d, *J* = 7.9 Hz, 2H, Ar-H), 5.45 (s, 1H, CHPh), 5.29 (d, *J* = 3.5 Hz, 1H, H-1'), 4.94, 4.87 (ABq, *J* = 12.2 Hz, 2H, CH_2NAP), 4.42 (d, *J* = 9.6 Hz, 1H, H-1), 4.27-4.26 (m, 2H, H-4, H-4'), 4.21-4.18 (br, 1H, H-6eq), 4.17 (dd, *J* = 10.4, 3.7 Hz, 1H, H-3'), 4.15-4.13 (br, 1H, H-6eq'), 4.00 (s, 1H, H-5'), 3.99-3.96 (m, 2H, H-6ax, H-6ax'), 3.93 (dd, *J* = 10.4, 3.5 Hz, 1H, H-2'), 3.87 (t, *J* = 9.6 Hz, 1H, H-2), 3.81 (s, 3H, OCH_3), 3.69 (dd, *J* = 9.6, 3.5 Hz, 1H, H-3), 3.38 (d, *J* = 1.1 Hz, 1H, H-5), 2.34 (s, 3H, CH_3), 1.56 (s, 3H, CH_3); ^{13}C NMR (125 MHz, $CDCl_3$) δ 170.3 (C), 165.9 (C), 138.5 (C), 137.6 (C), 135.6 (C), 134.2 (C), 133.4 (CH, Ar-C), 133.2 (C), 133.0 (C), 129.7 (CH, Ar-C), 129.5 (CH, Ar-C), 128.9 (CH, Ar-C), 128.2 (CH, Ar-C), 128.1 (CH, Ar-C), 127.9 (CH, Ar-C), 127.7 (CH, Ar-C), 127.3 (C), 126.3 (CH, Ar-C), 126.2 (CH, Ar-C), 126.1 (CH, Ar-C), 125.9 (CH, Ar-C), 125.6 (CH, Ar-C), 100.8 (CH, CHPh), 95.1 (CH, C-1'), 88.5 (CH, C-1), 76.3 (CH, C-3), 74.2 (CH, C-3'), 73.3 (CH, C-4'), 71.5(CH_2, CH_2NAP), 69.3 (CH_2, C-6), 69.2 (CH, C-5), 67.2 (CH, C-4), 66.4 (CH, C-2), 65.5 (CH_2, C-6'), 62.9 (CH, C-5'), 58.4 (CH, C-2'), 52.7 (CH_3, OCH_3), 25.6 (CH_3, CCH_3), 21.2 (CH_3, STol); HRMS (ESI) calcd for $C_{41}H_{43}N_3O_{11}SNa$ $[M + Na]^+$ 808.2511, found 808.2516.

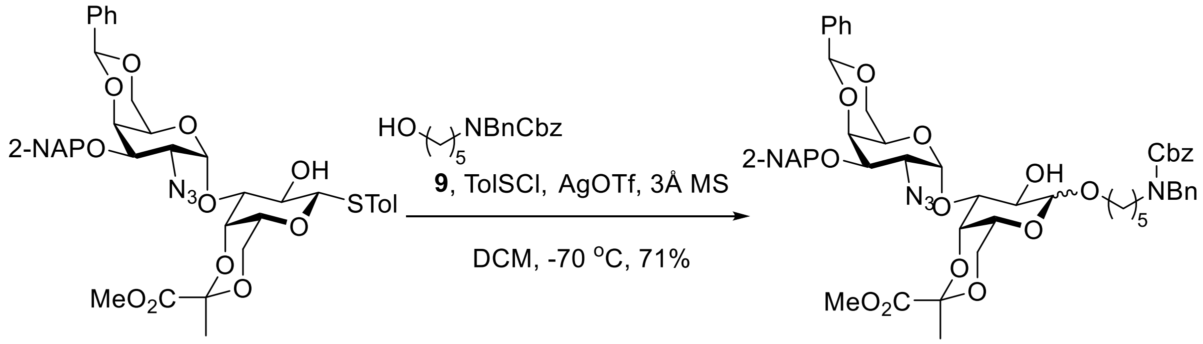

8

10

N-(Benzyl)-benzyloxycarbonyl-5-aminopentyl 4,6-*O*-[1-(*R*)-(methyloxycarbonyl)-ethylidene]-3-*O*-(2-azido-4,6-*O*-benzylidene-2-deoxy-3-*O*-naphthyl-α-D-galactopyranosyl)-β-D-galactopyranoside (10). To a suspension of the thioglycoside donor **8** (40 mg, 0.05 mmol), molecular sieves (3 Å, 30 mg) and acceptor **9** (26 mg, 0.08 mmol) in dry DCM (500 μL) was stirred at −70 °C individually under nitrogen atmosphere for 1 h. AgOTf (13 mg, 0.05 mmol) was added into the reaction mixture at −70 °C and stirred for 30 mins at same temperature. TolSCl (8 μL, 0.05 mmol) was added into the reaction mixture, and stirred at temperature −70 °C for 1.5 h judged by TLC. The solution was filtered through celite and washed with EtOAc. The filtrate was evaporated in vacuo to furnish the crude product, which was purified by flash chromatography to give the product **10** (best yield: 35 mg, 71%, α/β = 1:6); One-pot synthesis procedure: A solution of donor **7** (124 mg, 0.23 mmol) and freshly activated 3 Å molecular sieves (80 mg) in dry DCM (1.2 mL) was stirred at room temperature for 5 min, and cooled to −70 °C for 1 h, which was followed by addition of AgOTf (59 mg, 0.23 mmol) without touching the wall of the flask. After 5 min, orange-colored TolSCl (35 μL, 0.23 mmol) was added to the solution through a microsyringe. After the donor was completely consumed, according to TLC analysis (about 25 min at −70 °C), a solution of acceptor **6** (55 mg, 0.15 mmol) in dry THF (400 μL) was slowly added dropwise by using a syringe. The reaction mixture was worked at −70 °C under stirring for 1.5 h. Followed by sequential additions of the second acceptor **9** (75 mg, 0.23 mmol) in dry DCM (800 μL), AgOTf (39 mg,

0.15 mmol). The mixture was stirred for 5 min at −70 °C and then TolSCl (23 μL, 0.15 mmol) was added to the solution. The reaction mixture was warmed to −55 °C under stirring in 1 h. Then the mixture was diluted with DCM (20 mL) and filtered over Celite. The Celite was further washed with DCM until no organic compounds were observed in the filtrate by TLC analysis. All solutions in DCM were combined and washed twice with a saturated aqueous solution of $NaHCO_3$ (20 mL). The organic layer was collected and dried over $MgSO_4$. After removal of the solvent, the crude was purified by flash chromatography to give the product **10** (overall yield: 52 mg, 42%, α/β = 1:5.8). β-anomer: $[\alpha]^{33}_{D}$ +81.7 (*c* 2.3, CH_2Cl_2); IR (CH_2Cl_2) ν 3486, 2924, 2861, 2110, 1743, 1693, 1454, 1423, 1368, 1270, 1204, 1178, 1124, 1096, 1077, 1045, 984, 956, 876, 734, 699 cm^{-1}; ^1H NMR (500 MHz, $CDCl_3$) δ 7.85-7.77 (m, 4H, Ar-H), 7.54-7.45 (m, 5H, Ar-H), 7.38-7.17 (m, 13H, Ar-H), 5.45 (s, 1H, CHPh), 5.32 (d, *J* = 3.3 Hz, 1H, H-1'), 5.19-5.15 (m, 2H, CH_2Ph), 4.98-4.84 (m, 2H, CH_2NAP), 4.51-4.49 (m, 2H, CH_2Ph), 4.28 (s, 1H, H-4'), 4.23 (d, *J* = 2.0 Hz, 1H, H-4), 4.22 (dd, *J* = 10.9, 3.9 Hz, 1H, H-3'), 4.20-4.15 (m, 2H, H-1, H-6eq'), 4.09 (d, *J* = 13.2 Hz, 1H, H-6eq), 4.06 (s, 1H, H-5'), 4.00 (d, *J* = 13.2 Hz, 1H, H-6ax), 3.96-3.87 (m, 3H, H-6ax', H-2', H-2), 3.82 (s, 3H, OCH_3), 3.70-3.68 (br, 1H, H-3), 3.44-3.39 (br, 1H, GalOC*H*H), 3.30-3.22 (m, 3H, H-5, GalOCH*H*, CH_2), 1.62-1.51 (m, 7H, CH_2, CH_2, CH_3), 1.35-1.25 (m, 2H, CH_2); ^{13}C NMR (125 MHz, $CDCl_3$) δ 170.4 (C), 156.8 (C), 156.3 (C), 137.8 (C), 137.7 (CH), 136.8 (C), 136.6 (C), 135.7 (C), 133.2 (C), 133.0 (C), 128.9 (CH, Ar-C), 128.5 (CH, Ar-C), 128.4 (CH, Ar-C), 128.13 (CH, Ar-C), 128.09 (CH, Ar-C), 127.94 (CH, Ar-C), 127.88 (CH, Ar-C), 127.85 (CH, Ar-C), 127.6 (CH, Ar-C), 127.3 (CH, Ar-C), 127.2 (CH, Ar-C), 126.23 (CH, Ar-C), 126.16 (CH, Ar-C), 126.1 (CH, Ar-C), 125.9 (CH, Ar-C), 125.6 (CH, Ar-C), 103.3 (CH, C-1), 103.1 (CH, C-1), 100.8 (CH, CHPh), 95.1 (CH, C-1'), 74.9 (CH, C-3), 74.1 (CH, C-3'), 73.4 (CH, C-4'), 71.5 (CH_2,CH_2NAP), 70.0 (CH_2, linker-CH_2), 69.7 (CH_2, linker-CH_2), 69.3 (CH_2, C-6'), 69.1 (CH, C-2), 67.2 (CH_2, CH_2Ph), 66.9 (CH, C-4), 65.8 (CH, C-5), 65.3 (CH_2, C-6), 62.9 (CH, C-5'), 58.3 (CH, C-2'), 52.6 (CH_3, OCH_3), 50.4 (CH_2, CH_2Ph), 50.2 (CH_2), 46.9 (CH_2, linker-CH_2), 45.9 (CH_2, linker-

CH_2), 29.0 (CH_2, linker-CH_2), 28.9 (CH_2, linker-CH_2), 27.7 (CH_2, linker-CH_2), 27.2 (CH_2, linker-CH_2), 25.6 (CH_3, CCH_3), 23.2 (CH_2, linker-CH_2), 23.1 (CH_2, linker-CH_2); HRMS (ESI) calcd for $C_{54}H_{60}N_4O_{14}Na$ $[M + Na]^+$ 1011.3998, found 1011.3992. α-anomer: $[\alpha]^{33}_D$ +25.1 (*c* 1.0, CH_2Cl_2); IR (CH_2Cl_2) ν 3479, 2922, 2852, 2111, 1744, 1695, 1454, 1423, 1367, 1338, 1272, 1250, 1205, 1176, 1127, 1099, 1081, 1045, 995, 982, 955, 819, 751, 699 cm^{-1}; ^1H NMR (500 MHz, $CDCl_3$) δ 7.87-7.76 (m, 4H, Ar-H), 7.57-7.45 (m, 5H, Ar-H), 7.39-7.16 (m, 13H, Ar-H), 5.44 (s, 1H, CHPh), 5.26 (d, *J* = 2.9 Hz, 1H, H-1'), 5.18-5.15 (m, 2H, CH_2Ph), 4.98-4.88 (m, 3H, H-1, CH_2NAP), 4.49-4.48 (m, 2H, CH_2Ph), 4.27-4.19 (m, 4H, H-4', H-4, H-3', H-6eq'), 4.07-3.95 (m, 5H, H-2, H-5', H-6eq, H-6ax, H-6ax'), 3.88 (dd, *J* = 10.7, 2.9 Hz, 1H, H-2'), 3.84-3.83 (m, 4H, H-3, OCH_3), 3.68-3.60 (br, 1H, GalOC*H*H), 3.53 (s, 1H, H-5), 3.47-3.39 (br, 1H, GalOCH*H*), 3.24-3.19 (m, 2H, CH_2), 1.68-1.54 (m, 7H, CH_2, CH_2, CH_3), 1.35-1.22 (m, 2H, CH_2); ^{13}C NMR (125 MHz, $CDCl_3$) δ 170.3 (C), 156.2 (C), 137.8 (C), 137.7 (C), 136.7 (C), 135.6 (C), 133.2 (C), 133.0 (C), 128.9 (CH, Ar-C), 128.54 (CH, Ar-C), 128.51 (CH, Ar-C), 128.4 (CH, Ar-C), 128.2 (CH, Ar-C), 128.1 (CH, Ar-C), 127.9 (CH, Ar-C), 127.8 (CH, Ar-C), 127.7 (CH, Ar-C), 127.3 (CH, Ar-C), 127.2 (CH, Ar-C), 126.3 (CH, Ar-C), 126.2 (CH, Ar-C), 126.1 (CH, Ar-C), 125.9 (CH, Ar-C), 125.7 (CH, Ar-C), 100.9 (CH,CHPh), 98.8 (CH, C-1), 95.8 (CH, C-1'), 73.9 (CH, C-3'), 73.3 (CH, C-4'), 71.5 (CH_2, CH_2NAP), 69.4 (CH_2, C-6'), 68.2 (CH_2, linker-CH_2), 67.9 (CH, C-2), 67.2 (CH_2, CH_2Ph), 67.0 (CH, C-4), 65.5 (CH_2, C-6), 63.0 (CH, C-5'), 61.8 (CH), 58.3 (CH, C-2'), 52.7 (CH_3, OCH_3), 50.5 (CH_2, CH_2Ph), 50.3 (CH_2, CH_2Ph), 47.0 (CH_2, linker-CH_2), 29.0 (CH_2, linker-CH_2), 27.2 (CH_2, linker-CH_2), 25.6 (CH_3, CCH_3), 23.3 (CH_2, linker-CH_2); HRMS (ESI) calcd for $C_{54}H_{60}N_4O_{14}Na$ $[M + Na]^+$ 1011.3998, found 1011.4008.

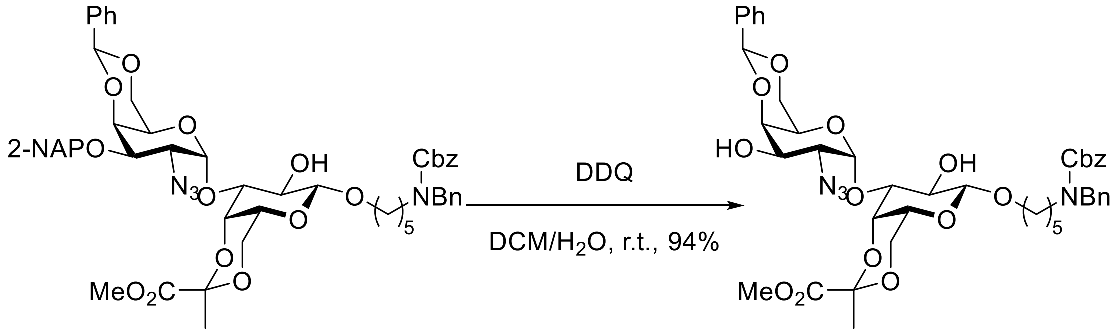

10 **11**

***N*-(Benzyl)-benzyloxycarbonyl-5-aminopentyl 4,6-*O*-[1-(*R*)-(methyloxycarbonyl)-ethylidene]-3-*O*-(2-azido-4,6-*O*-benzylidene-2-deoxy-α-D-galactopyranosyl)-β-D-galactopyranoside (11)**. The compound **10** (273 mg, 0.28 mmol) was dissolved in a mixture of DCM/H_2O (9/1 = v/v, 3.33 mL). Then DDQ (254 mg, 1.12 mmol) was added at room temperature. After 3 h, the reaction mixture was diluted by water and extracted with DCM for 3 times. The combined organic phase was then washed with saturated $NaHCO_3$(aq) and dried over $MgSO_4$. After removal of the solvent, the crude product was purified by column chromatography to give product **11** (221 mg, 94%). $[\alpha]^{28}_{D}$ +54.3 (*c* 0.8, CH_2Cl_2); IR (CH_2Cl_2) ν 3510, 2934, 2861, 2111, 1742, 1688, 1474, 1454, 1425, 1370, 1274, 1229, 1205, 1182, 1127, 1091, 1079, 1042, 984, 748, 735, 699 cm^{-1}; ^1H NMR (500 MHz, $CDCl_3$) δ 7.49-7.48 (m, 2H, Ar-H), 7.37-7.18 (m, 13H, Ar-H), 5.55 (s, 1H, CHPh), 5.33 (s, 1H, H-1'), 5.19-5.16 (m, 2H, CH_2Ph), 4.50-4.45 (m, 2H, CH_2Ph), 4.30 (t, *J* = 8.5 Hz, 1H, H-3'), 4.25 (s, 1H, H-4), 4.23-3.87 (m, 9H, H-5', H-6eq', H-1, H-4', H-6eq, H-6ax', H-6ax, H-2, GalOC*H*H), 3.82 (s, 3H, OCH_3), 3.70 (d, *J* = 8.9 Hz, 1H, H-3), 3.54 (d, *J* = 10.0 Hz, 1H, H-2'), 3.45-3.40 (br, 1H, GalOCH*H*), 3.30-3.22 (m, 3H, H-5, CH_2), 1.61-1.56 (m, 7H, CH_2, CH_2, CH_3), 1.35-1.26 (m, 2H, CH_2); ^{13}C NMR (125 MHz, $CDCl_3$) δ 170.4 (C), 156.8 (C), 156.3 (C), 137.7 (C), 137.4 (C), 136.6 (C), 129.2 (CH, Ar-C), 128.5 (CH, Ar-C), 128.4 (CH, Ar-C), 128.2 (CH, Ar-C), 127.9 (CH, Ar-C), 127.8 (CH, Ar-C), 127.3 (CH, Ar-C), 127.1 (CH, Ar-C), 126.2 (CH, Ar-C), 103.2 (CH, C-1), 103.0 (CH), 101.1 (CH, CHPh), 98.8 (CH), 94.9 (CH, C-1'), 75.6 (CH, C-4'), 74.8 (CH, C-3),

70.0 (CH_2, linker-CH_2), 69.7 (CH_2, linker-CH_2), 69.2 (CH_2, C-6'), 69.1 (CH, C-2), 67.2 (CH_2, CH_2Ph), 66.7 (CH, C-4), 66.5 (CH, C-3'), 65.7 (CH, C-5), 65.4 (CH2, C-6), 62.8 (CH, C-5'), 60.0 (CH, C-2'), 52.6 (CH_3, OCH_3), 50.3 (CH_2, CH_2Ph), 50.2 (CH_2, CH_2Ph), 47.0 (CH_2, linker-CH_2), 29.0 (CH_2, linker-CH_2), 28.8 (CH_2, linker-CH_2), 27.7 (CH_2, linker-CH_2), 27.2 (CH_2, linker-CH_2), 25.5 (CH_3, CCH_3), 23.2 (CH_2, linker-CH_2), 23.0 (CH_2, linker-CH_2); HRMS (ESI) calcd for $C_{43}H_{52}N_4O_{14}Na$ $[M + Na]^+$ 871.3372, found 871.3363.

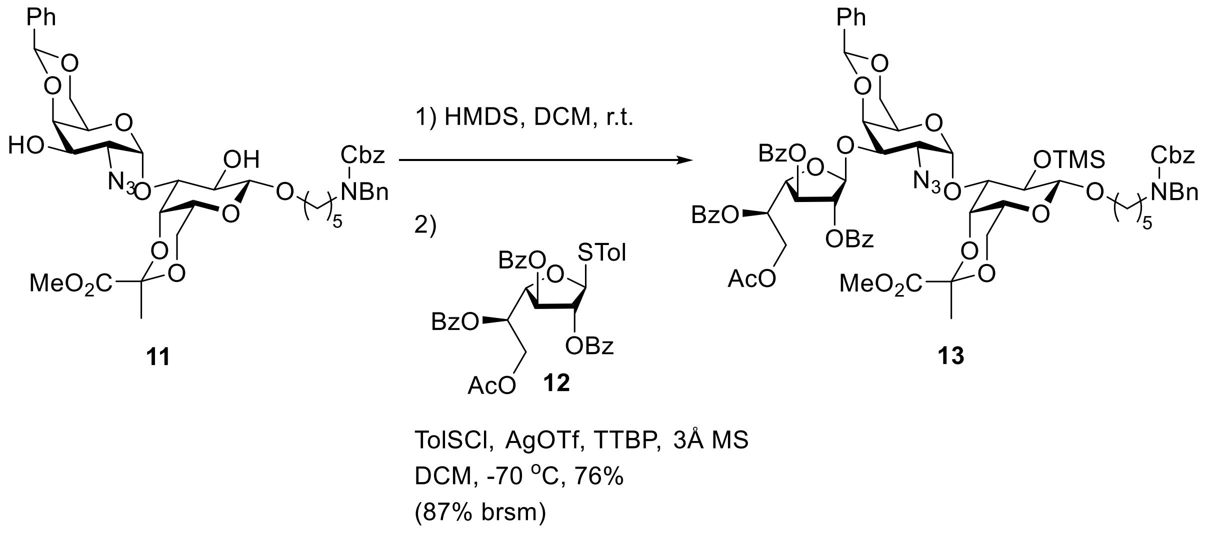

_N_-(Benzyl)-benzyloxycarbonyl-5-aminopentyl 4,6-_O_-[1-(_R_)-(methyloxycarbonyl)-ethylidene-2-_O_-trimethylsilyl]-3-_O_-[(2-azido-4,6-_O_-benzylidene-2-deoxy)-3-_O_-(6-_O_-acetyl-2,3,5-tri-_O_-benzoyl-β-D-galactofuranosyl)-α-D-galactopyranosyl]-β-D-galactopyranoside (13). HMDS (43 μL, 0.20 mmol) was added at room temperature under N_2 to a suspension of acceptor **11** (128 mg, 0.15 mmol) in DCM (1.5 mL) for 17 h. The solution was evaporated in vacuo to furnish the silylated acceptor. To a suspension of the silylated acceptor, molecular sieves (3 Å, 165 mg) and donor **12**[4] (192 mg, 0.30 mmol) in dry DCM (5 mL) was stirred at −70 °C individually under nitrogen atmosphere for 1 h. AgOTf (79 mg, 0.30 mmol) and TTBP (51mg, 0.20 mmol) was added into the reaction mixture at−70 °C and stirred for 30 mins at same temperature. TolSCl (46 μL, 0.30 mmol) was added into the reaction mixture, and stirred at temperature −70 °C for 3.5 h judged by TLC. The solution was filtered

through celite and washed with EtOAc. The filtrate was evaporated *in vacuo* to furnish the crude product, which was purified by flash chromatography to give the product **13** (best yield: 164 mg, 76% (87% brsm)). $[\alpha]^{28}_{D}$ +25.0 (*c* 1.0, CH_2Cl_2); IR (CH_2Cl_2) ν 2925, 2109, 1719, 1601, 1452, 1422, 1367, 1315, 1265, 1227, 1177, 1159, 1096, 1068, 1041, 1026, 984, 923, 875, 839, 796, 734, 712, 700 cm^{-1}; ^1H NMR (500 MHz, $CDCl_3$) δ 8.16 (d, *J* = 7.4 Hz, 2H, Ar-H), 8.01-7.97 (m, 2H, Ar-H), 7.80 (d, *J* = 7.4 Hz, 2H, Ar-H), 7.61-7.15 (m, 24H, Ar-H), 6.03 (td, *J* = 5.4, 2.9 Hz, 1H, H-5''), 5.59 (s, 1H, CHPh), 5.50-5.48 (m, 3H, H-1'', H-1', H-3''), 5.36 (s, 1H, H-4'), 5.18-5.16 (m, 2H, CH_2Ph), 5.08-5.03 (br, 1H, H-5'), 4.57 (d, *J* = 10.9 Hz, 1H, H-6a''), 4.52-4.48 (br, 1H, H-6b''), 4.45-4.40 (m, 2H, CH_2Ph), 4.34-4.32 (m, 4H, H-4, H-6eq', H-6ax', H-1), 4.22 (t, *J* = 8.7 Hz, 1H, H-2), 4.19 (d, *J* = 3.2 Hz, 1H, H-4''), 4.11 (d, *J* = 13.2 Hz, 1H, H-6eq), 4.05-3.95 (m, 4H, H-3'', H-2', H-3, H-6ax), 3.88-3.78 (m, 5H, GalOC*H*H, OCH_3, H-2''), 3.32-3.08 (m, 4H, H-5, GalOCH*H*, CH_2), 2.00 (s, 3H, OAc), 1.65-1.61 (m, 7H, CH_2, CH_2, CH_3), 1.32-1.26 (m, 2H, CH_2), -0.12 (s, 9H, CH_3, CH_3, CH_3); ^{13}C NMR (125 MHz, $CDCl_3$) δ 170.6 (C), 170.4 (C), 165.9 (C), 165.8 (C), 165.7 (C), 156.6 (C), 156.1 (C), 150.3 (C), 137.9 (CH, Ar-C), 136.9 (CH, Ar-C), 136.7 (CH, Ar-C), 133.7 (CH, Ar-C), 133.6 (CH, Ar-C), 133.2 (CH, Ar-C), 130.1 (CH, Ar-C), 129.9 (CH, Ar-C), 129.7 (CH, Ar-C), 129.0 (CH, Ar-C), 128.9 (CH, Ar-C), 128.5 (CH, Ar-C), 128.1 (CH, Ar-C), 127.9 (CH, Ar-C), 127.8 (CH, Ar-C), 127.22 (CH, Ar-C), 127.15 (CH, Ar-C), 126.2 (CH, Ar-C), 104.8 (CH, C-1'), 101.6 (CH, C-1), 101.1 (CH, CHPh), 98.9 (CH), 93.4 (CH, C-1''), 82.8 (CH, C-4'), 81.3 (CH, C-5'), 78.3 (CH, C-3''), 76.5 (CH, C-4''), 75.2 (CH, C-3'), 70.6 (CH, C-2), 70.2 (CH, C-5''), 70.1 (CH_2, linker-CH_2), 70.0 (CH_2, linker-CH_2), 69.4 (CH_2, C-6'), 68.5 (CH, C-2'), 67.1 (CH_2, CH_2Ph), 65.9 (CH, C-4), 65.5 (CH, C-5), 65.4 (CH_2, C-6), 63.7 (CH_2, C-6''), 63.3 (CH, C-3), 59.5 (CH, C-2''), 52.7 (CH_3, OCH_3), 50.2 (CH_2, CH_2Ph), 49.9 (CH_2, CH_2Ph), 46.9 (CH_2, linker-CH_2), 45.9 (CH_2, linker-CH_2), 29.7 (CH_2, linker-CH_2), 29.2 (CH_2, linker-CH_2), 27.7 (CH_2, linker-CH_2), 27.2 (CH_2, linker-CH_2), 25.6 (CH_3, CCH_3), 20.7 (CH_3, OAc), 0.2 (CH_3, TMS); HRMS (ESI) calcd for $C_{75}H_{84}N_4O_{23}SiNa$ $[M + Na]^+$ 1459.5188, found 1459.5186.

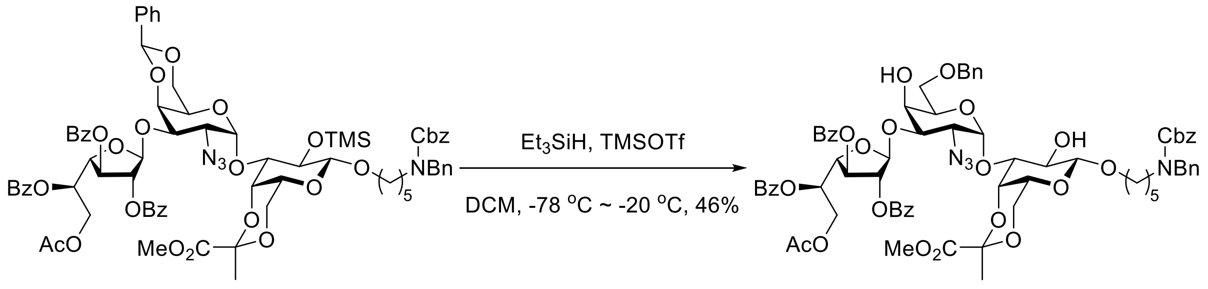

13 **14**

***N*-(Benzyl)-benzyloxycarbonyl-5-aminopentyl 4,6-*O*-[1-(*R*)-(methyloxycarbonyl)-ethylidene-2-*O*-trimethylsilyl]-3-*O*-[(2-azido-6-*O*-benzyl-2-deoxy)-3-*O*-(6-*O*-acetyl-2,3,5-tri-*O*-benzoyl-β-D-galactofuranosyl)-α-D-galactopyranosyl]-β-D-galactopyranoside (14)**. To a solution of compound **13** (45 mg, 0.03 mmol) in dry DCM (0.7 mL) was added Et_3SiH (29 μL, 0.18 mmol). The reaction pre-stirred at -78 °C for 1 h. And then injected TMSOTf (14 μL, 0.07 mmol) at −78 °C to −20 °C for 49 h judged by TLC. After the reaction was finished later, quenching by Et_3N (1 mL). The mixture was extracted with EtOAc (20 mL × 3) and water (20 mL). The organic layers were combined, dried over anhydrous $MgSO_4$, filtered, and evaporated under reduced pressure. The crude product was purified by flash column chromatography to give colorless oil product **14** (19 mg, 46%). $[\alpha]^{27}_{D}$ +7.4 (*c* 1.0, CH_2Cl_2); IR (CH_2Cl_2) ν 3436, 2923, 2853, 2108, 1720, 1601, 1495, 1452, 1423, 1368, 1314, 1262, 1228, 1178, 1095, 1069, 1042, 1026, 982, 874, 802, 735, 712 cm^{-1}; ^1H NMR (500 MHz, $CDCl_3$) δ 8.15 (dd, *J* = 8.2, 1.1 Hz, 2H, Ar-H), 7.97 (s, 2H, Ar-H), 7.76 (dd, *J* = 8.2, 1.1 Hz, 2H, Ar-H), 7.59-7.55 (br, 1H, Ar-H), 7.51-7.47 (m, 2H, Ar-H), 7.41-7.15 (m, 21H, Ar-H), 6.01 (td, *J* = 5.6, 2.9 Hz, 1H, H-5''), 5.56 (d, *J* = 3.8 Hz, 1H, H-1'), 5.49-5.48 (m, 2H, H-1'', H-4''), 5.32 (d, *J* = 0.4 Hz, 1H, H-4'), 5.17-5.16 (m, 2H, CH_2Ph), 5.01-4.97 (br, 1H, H-5'), 4.67 (ABq, *J* = 12.0 Hz, 1H, CH_2Ph), 4.59 (ABq, *J* = 12.0 Hz, 1H, CH_2Ph), 4.57-4.55 (br, 1H, H-6a''), 4.48-4.42 (m, 3H, H-6b'', CH_2Ph), 4.38 (d, *J* = 3.7 Hz, 1H, H-4), 4.322-4.317 (m, 2H, H-2'', H-3''), 4.23-4.22 (br, 1H, H-1), 4.16 (dd, *J* = 9.5, 7.9 Hz, 1H, H-2), 4.08 (d, *J* = 12.9 Hz, 1H, H-6eq), 3.97 (dd, *J* = 11.0, 5.3 Hz, 1H, H-6a'), 3.91-3.84 (m, 5H, H-6b', H-6ax, H-3', H-3, GalOC*H*H), 3.80 (s,

3H, OCH_3), 3.68 (dd, *J* = 10.5, 3.8 Hz, 1H, H-2'), 3.37-3.04 (m, 4H, GalOCH*H*, H-5, CH_2), 2.35-2.32 (br, 1H, OH), 1.98 (s, 3H, OAc), 1.75-1.56 (m, 7H, CH_2, CH_2, CH_3), 1.40-1.17 (m, 2H, CH_2); ^{13}C NMR (125 MHz, $CDCl_3$) δ 170.6 (C), 170.5 (C), 166.0 (C), 165.9 (C), 165.7 (C), 156.7 (C), 156.1 (C), 137.9 (CH, Ar-C), 137.7 (CH, Ar-C), 136.9 (CH, Ar-C), 136.8 (CH, Ar-C), 133.5 (CH, Ar-C), 133.2 (CH, Ar-C), 130.1 (CH, Ar-C), 130.0 (CH, Ar-C), 129.73 (CH, Ar-C), 129.66 (CH, Ar-C), 129.1 (CH, Ar-C), 128.5 (CH, Ar-C), 128.4 (CH, Ar-C), 127.9 (CH, Ar-C), 127.8 (CH, Ar-C), 127.6 (CH, Ar-C), 127.24 (CH, Ar-C), 127.19 (CH, Ar-C), 105.2 (CH, C-1''), 101.6 (CH, C-1), 98.9 (CH), 92.5 (CH, C-1'), 82.1 (CH, C-4'), 81.3 (CH, C-5'), 77.9 (CH, C-4''), 74.8 (CH, C-3'), 73.4 (CH_2, CH_2Ph), 71.1 (CH_2, C-6'), 71.0 (CH, C-2), 70.4 (CH, C-3''), 70.3 (CH_2, linker-CH_2), 70.2 (CH, C-5''), 68.6 (CH, C-2''), 68.2 (CH, C-3), 67.1 (CH_2, CH_2Ph), 65.9 (CH, C-4), 65.5 (CH_2, C-6), 65.4 (CH, C-5), 63.8 (CH_2, C-6''), 60.3 (CH, C-2'), 52.7 (CH_3, OCH_3), 50.2 (CH_2, CH_2Ph), 50.0 (CH_2, CH_2Ph), 46.9 (CH_2, linker-CH_2), 29.7 (CH_2, linker-CH_2), 29.33 (CH_2, linker-CH_2), 29.25 (CH_2, linker-CH_2), 25.5 (CH_3, CCH_3), 22.9 (CH_2, linker-CH_2), 22.7 (CH_2, linker-CH_2), 20.7 (CH_3, OAc); HRMS (ESI) calcd for $C_{54}H_{60}N_4O_{14}Na$ $[M + Na]^+$ 1389.4949, found 1389.4943.

3. Crystal data and structure refinement for compound 6

Identification code	i18300	
Empirical formula	$C_{34}H_{46}O_{15}S_2$	
Formula weight	758.83	
Temperature	100.0(2) K	
Wavelength	0.71073 Å	
Crystal system	Monoclinic	
Space group	$P2_1$	
Unit cell dimensions	*a* = 16.1017(4) Å	α = 90°.
	b = 7.7608(2) Å	β = 93.3680(10)°.
	c = 28.8344(8) Å	γ = 90°.
Volume	3596.98(16) $Å^3$	
Z	4	
Density (calculated)	1.401 Mg/m^3	
Absorption coefficient	0.219 mm^{-1}	
F(000)	1608	
Crystal size	0.363 × 0.252 × 0.052 mm^3	
Theta range for data collection	2.123 to 27.103°.	
Index ranges	-20<=h<=20, -9<=k<=9, -36<=l<=36	
Reflections collected	122169	
Independent reflections	15836 [R(int) = 0.0855]	
Completeness to theta = 25.242°	99.8 %	
Absorption correction	Numerical	
Max. and min. transmission	1 and 0.8971	
Refinement method	Full-matrix least-squares on F^2	
Data / restraints / parameters	15836 / 5 / 963	
Goodness-of-fit on F^2	1.027	
Final R indices [I>2sigma(I)]	R1 = 0.0364, wR2 = 0.0781	
R indices (all data)	R1 = 0.0459, wR2 = 0.0830	
Absolute structure parameter	-0.01(3)	
Extinction coefficient	n/a	
Largest diff. peak and hole	0.301 and -0.230 e.$Å^{-3}$	

4. References

[1] Fu, J.; Laval, S.; Yu, B. Total Synthesis of Nucleoside Antibiotics Plicacetin and Streptcytosine A. *J. Org. Chem.* **2018**, *83*, 7076-7084.

[2] Koeller, K. M.; Smith, M. E. B.; Wong, C.-H. Chemoenzymatic Synthesis of PSGL-1 Glycopeptides: Sulfation on Tyrosine Affects Glycosyltransferase-Catalyzed Synthesis of the *O*-Glycan. *Bioorg. Med. Chem.* **2000**, *8,* 1017-1025.

[3] Yang, Y.-Y.; Ficht, S.; Brik, A.; Wong, C.-H. Sugar-Assisted Ligation in Glycoprotein Synthesis. *J. Am. Chem. Soc.* **2007**, *129,* 7690-7701.

[4] Lin, M.-H.; Chang, C.-W.; Chiang, T.-Y.; Dhurandhare, V. M.; Wang, C.-C. Thiocarbonyl as a Switchable Relay-Auxiliary Group in Carbohydrate Synthesis. *Org. Lett.* **2021**, *23*, 7313-7318.

5. NMR spectra

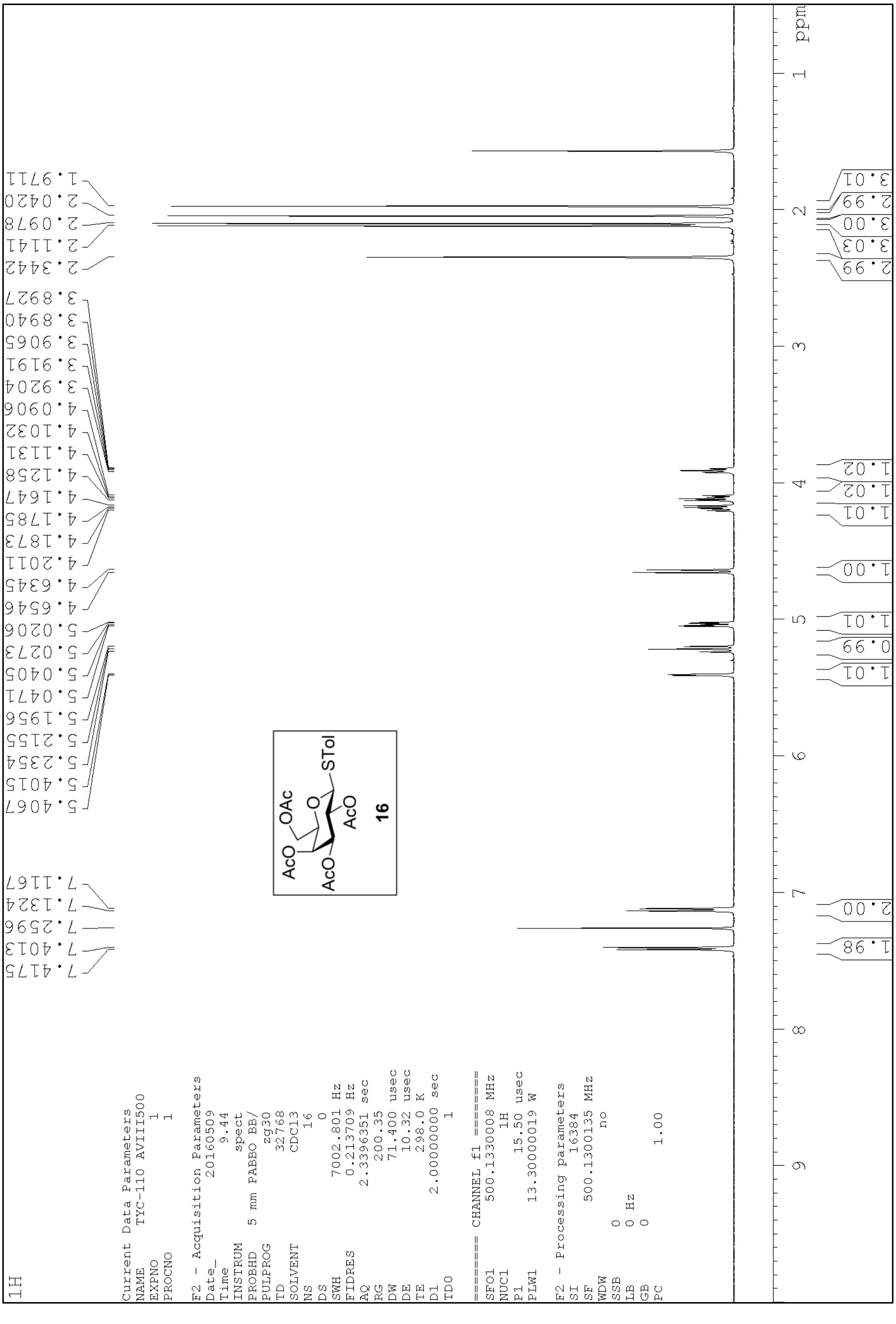

The ^{1}H spectrum in $CDCl_3$ of compound 16.

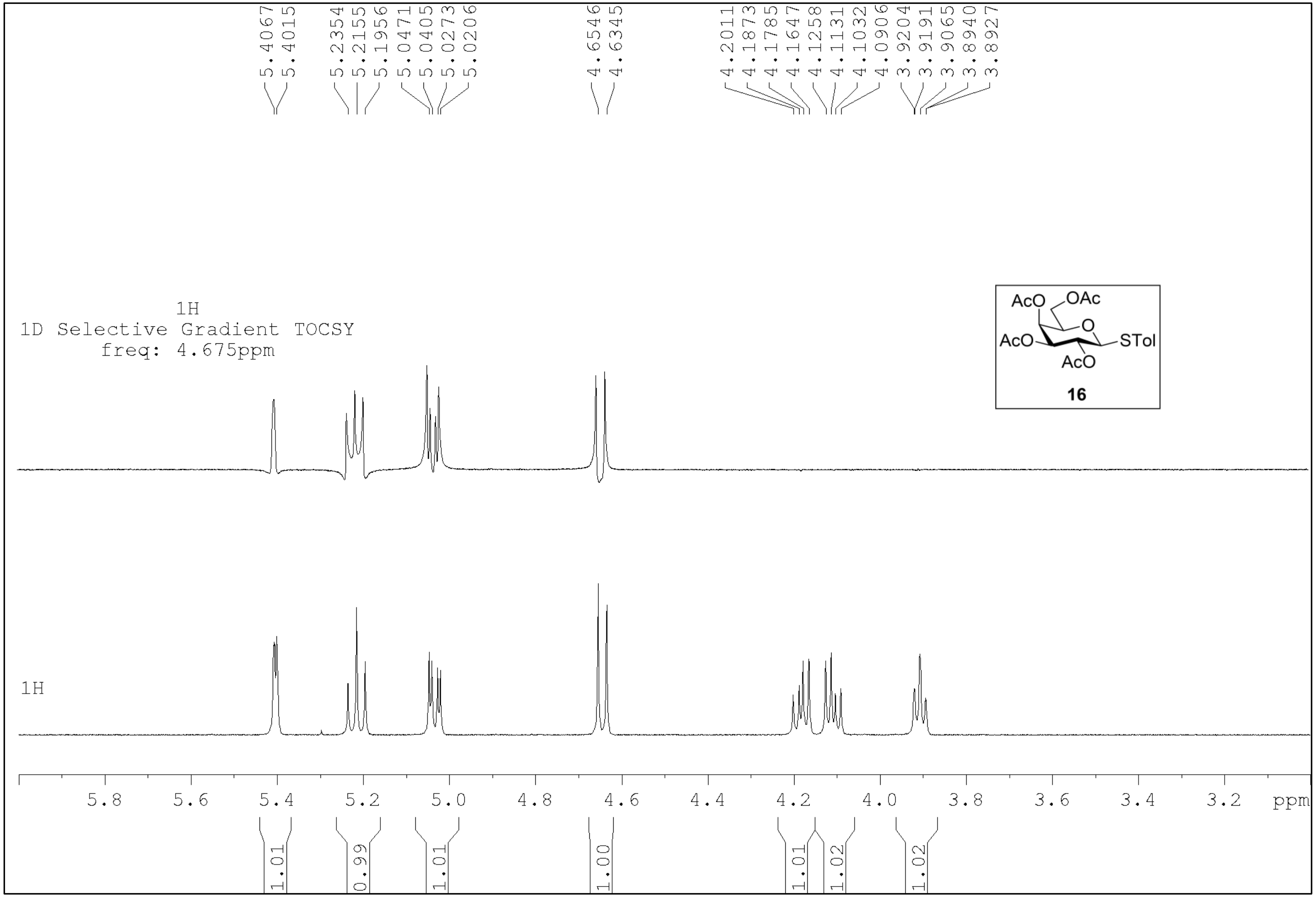

The 1D-TOCSY spectrum in $CDCl_3$ of compound 16.

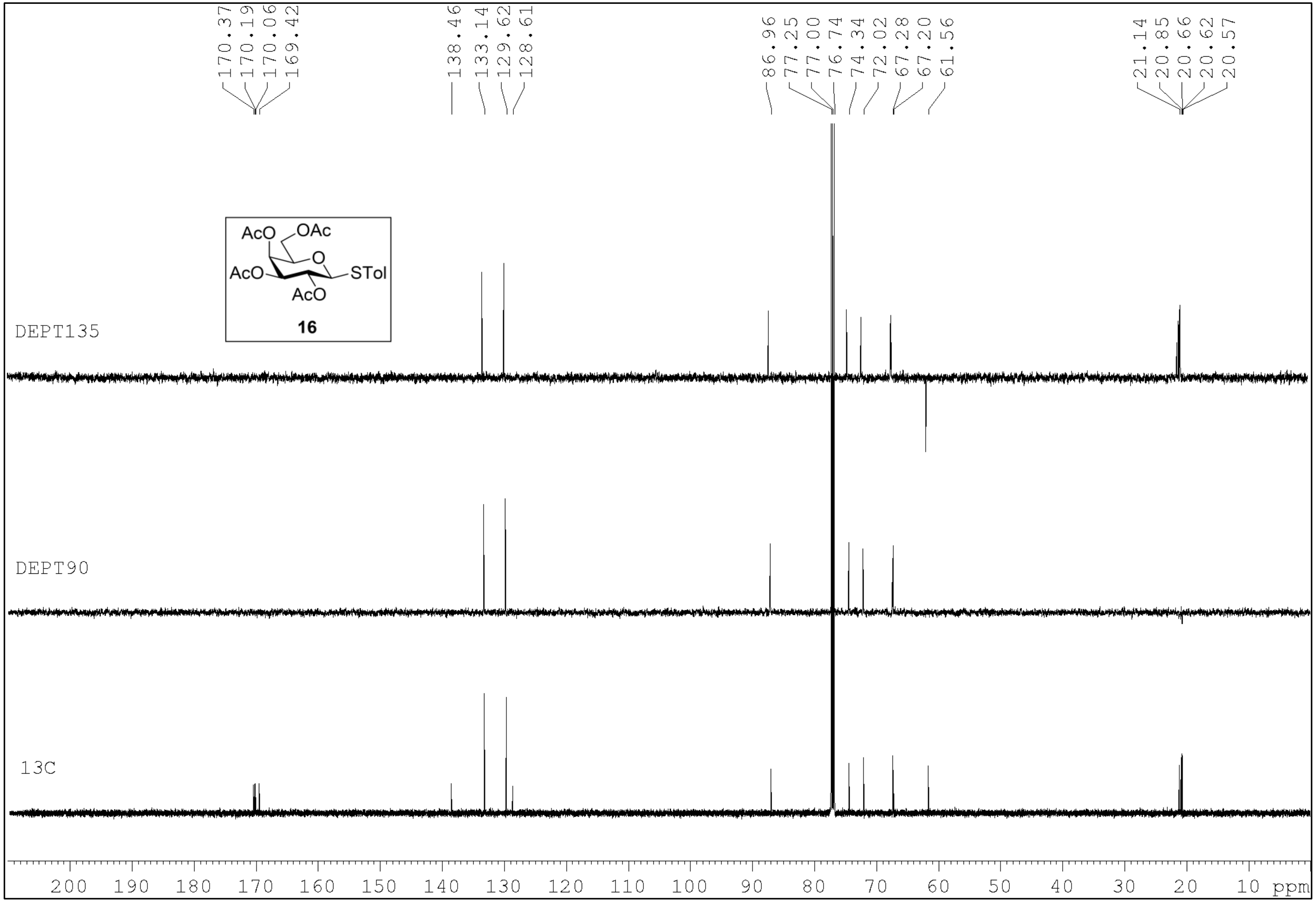

The ^{13}C spectrum in $CDCl_3$ of compound 16.

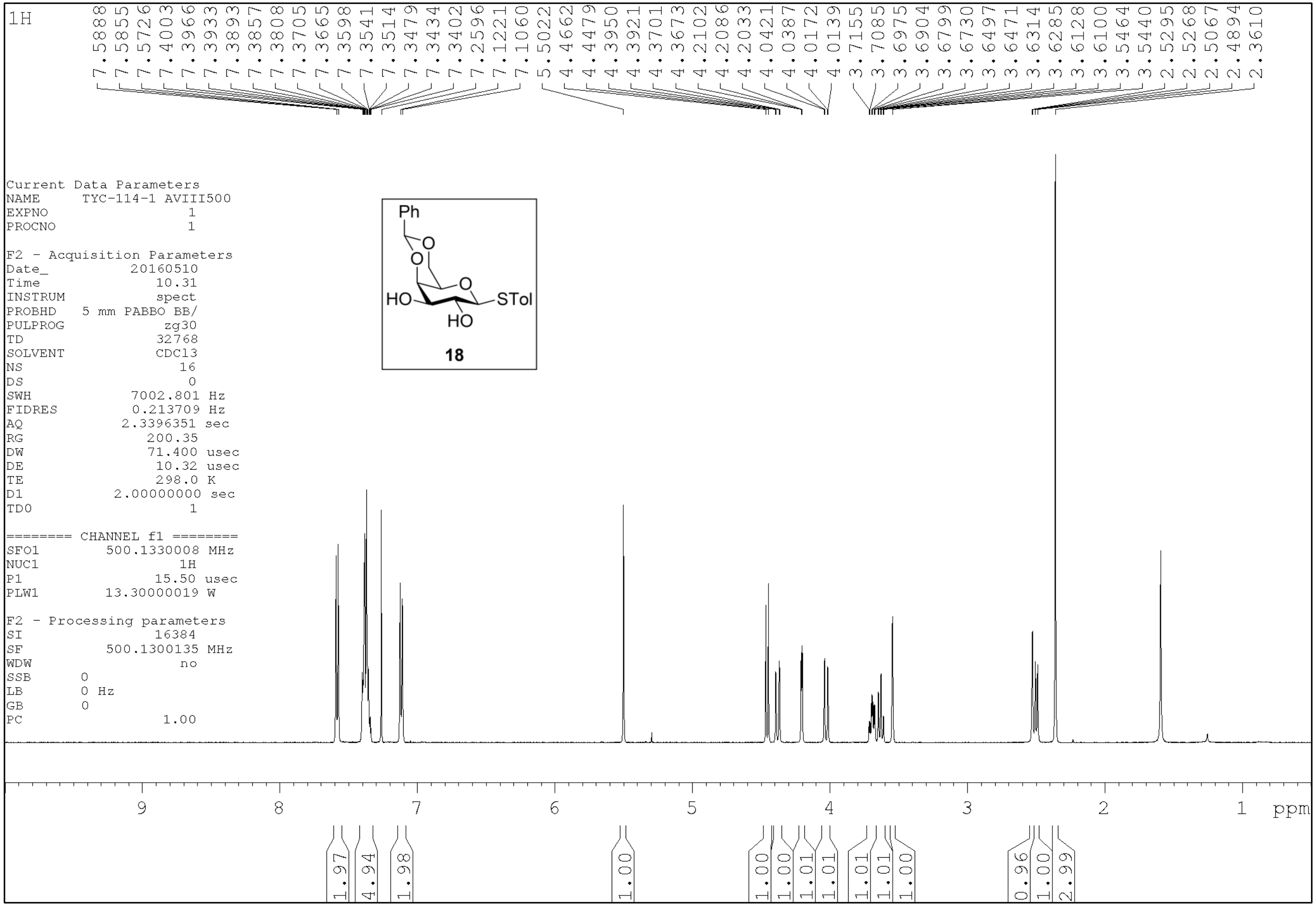

The ^{1}H spectrum in $CDCl_3$ of compound 18.

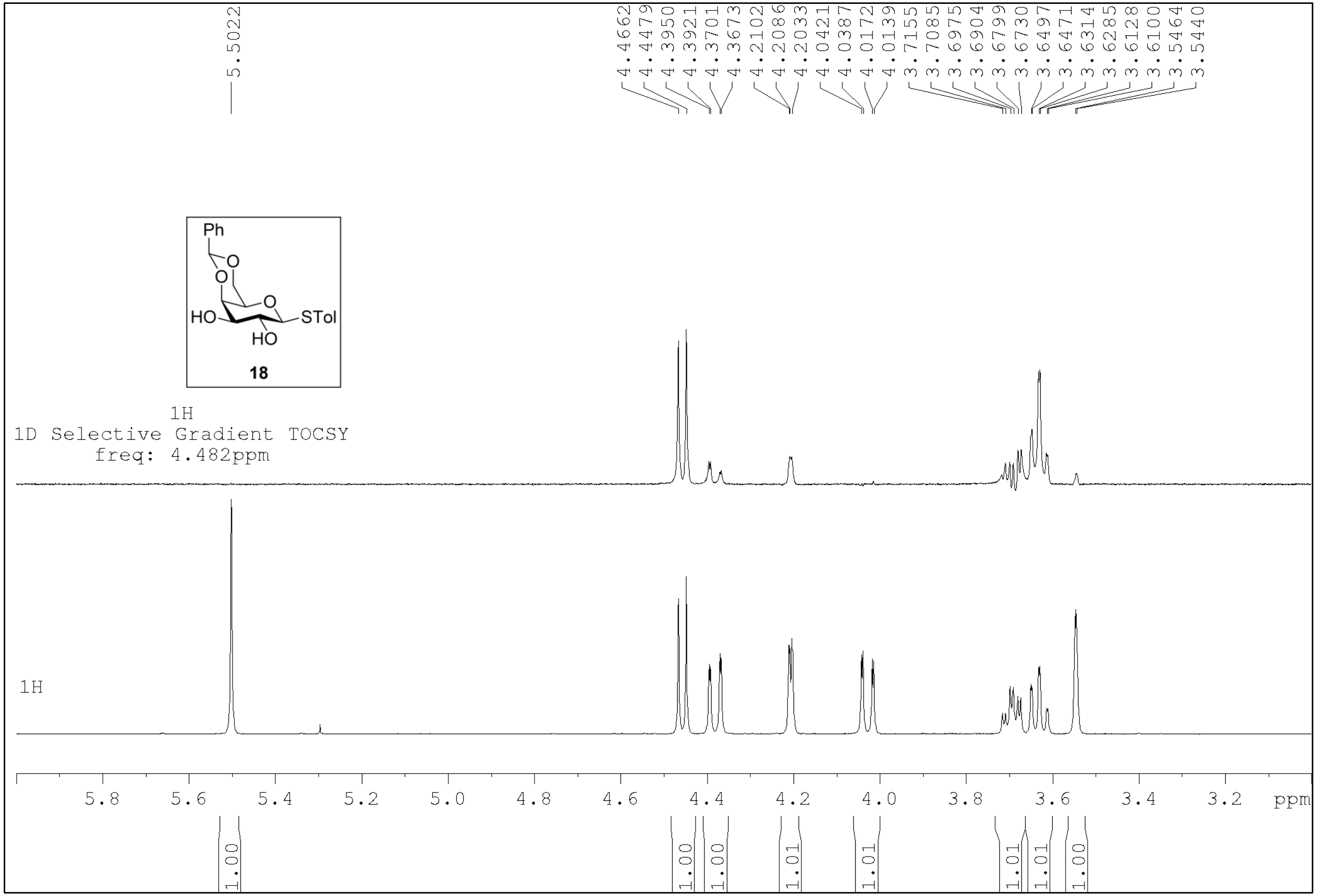

The 1D-TOCSY spectrum in $CDCl_3$ of compound 18.

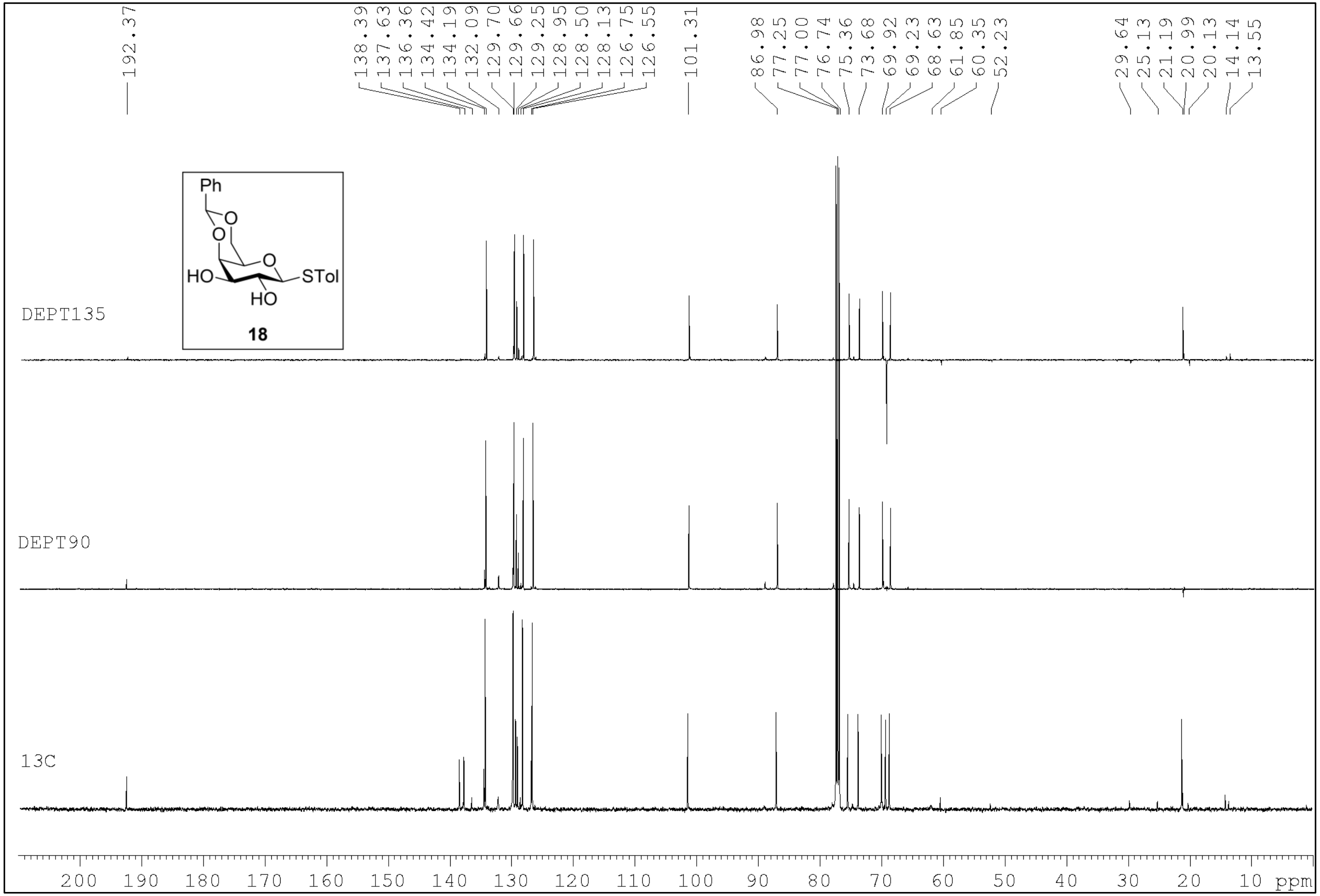

The ^{13}C spectrum in $CDCl_3$ of compound 18.

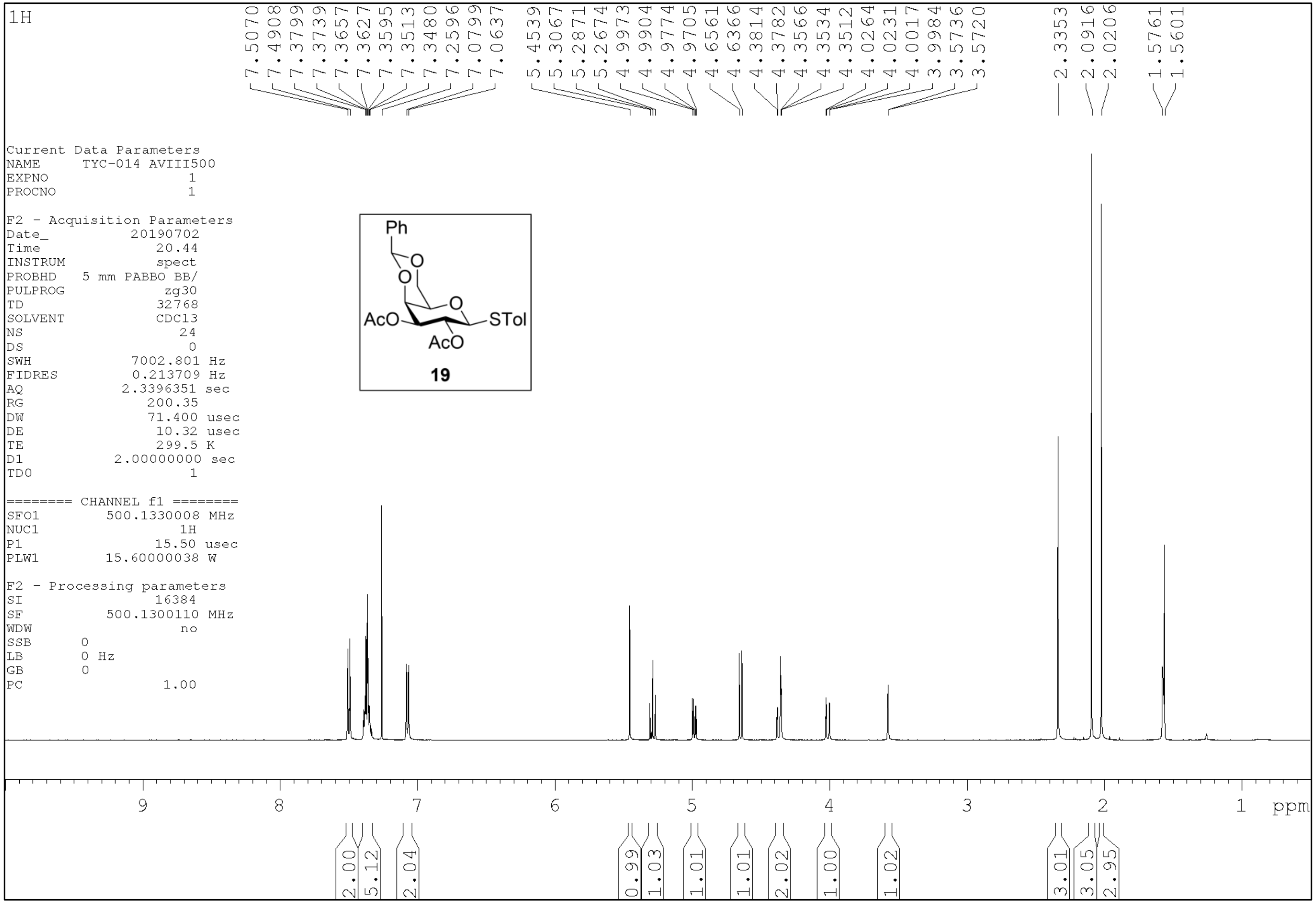

The ^{1}H spectrum in $CDCl_3$ of compound 19.

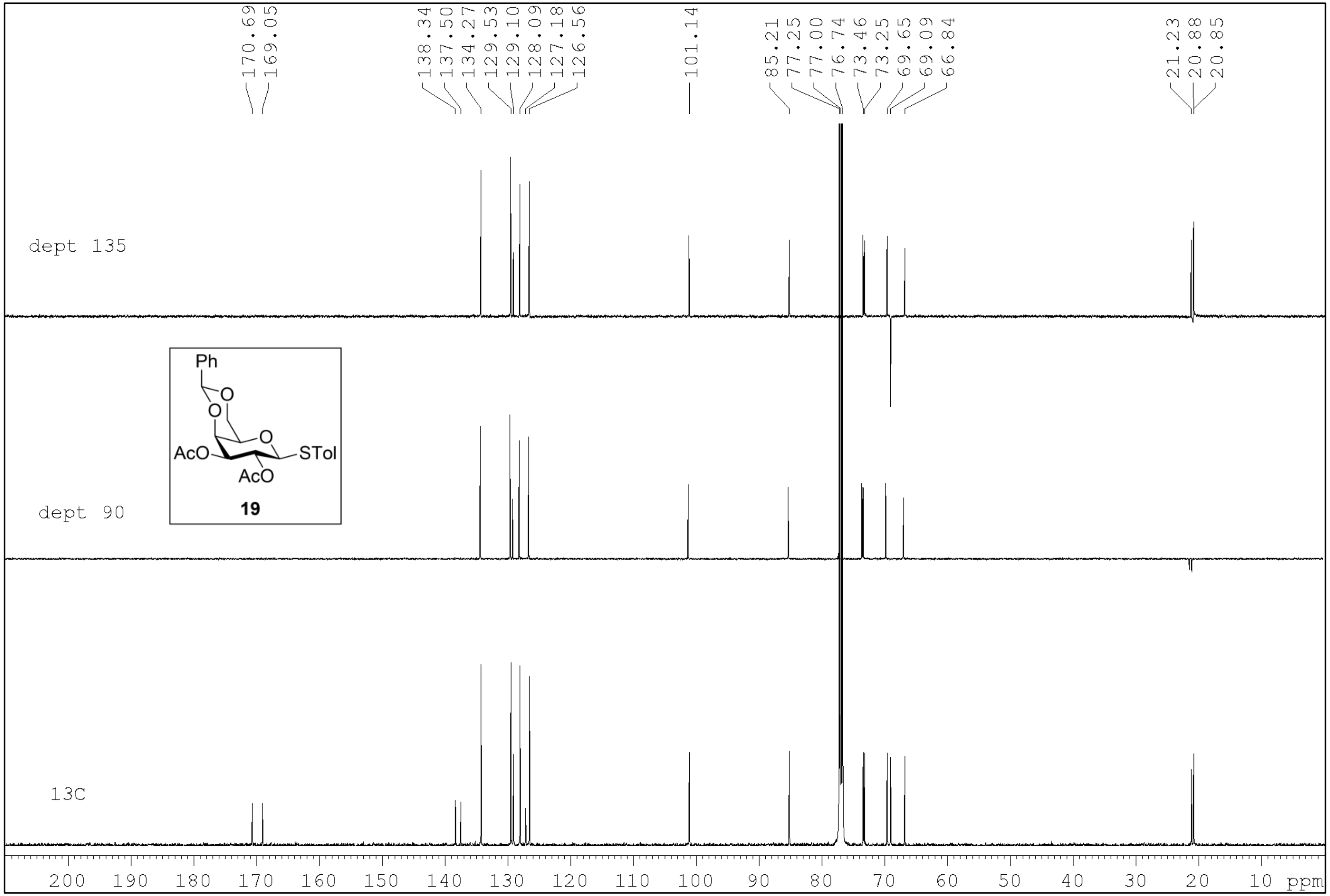

The ^{13}C spectrum in $CDCl_3$ of compound 19.

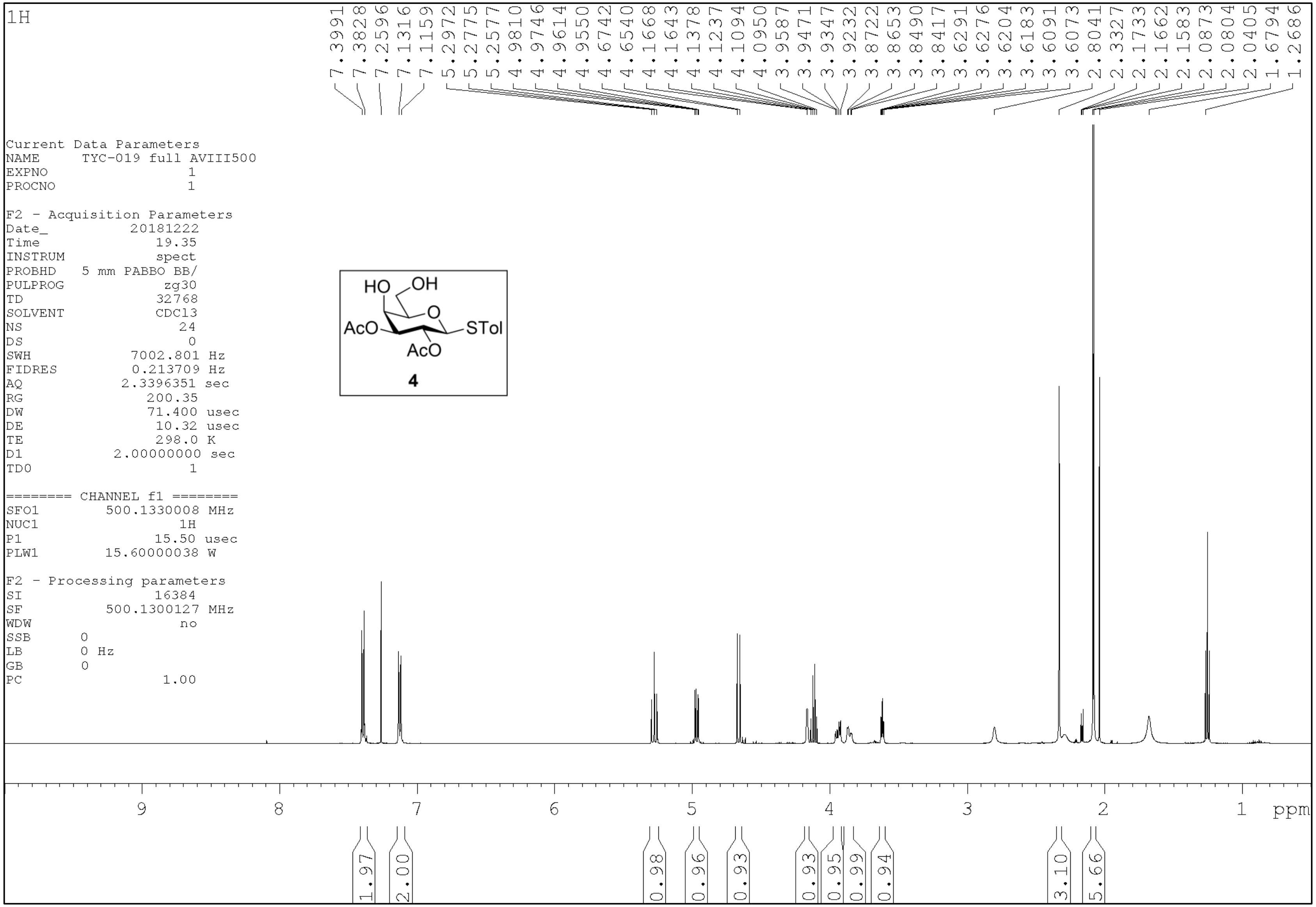

The ^1H spectrum in $CDCl_3$ of compound 4.

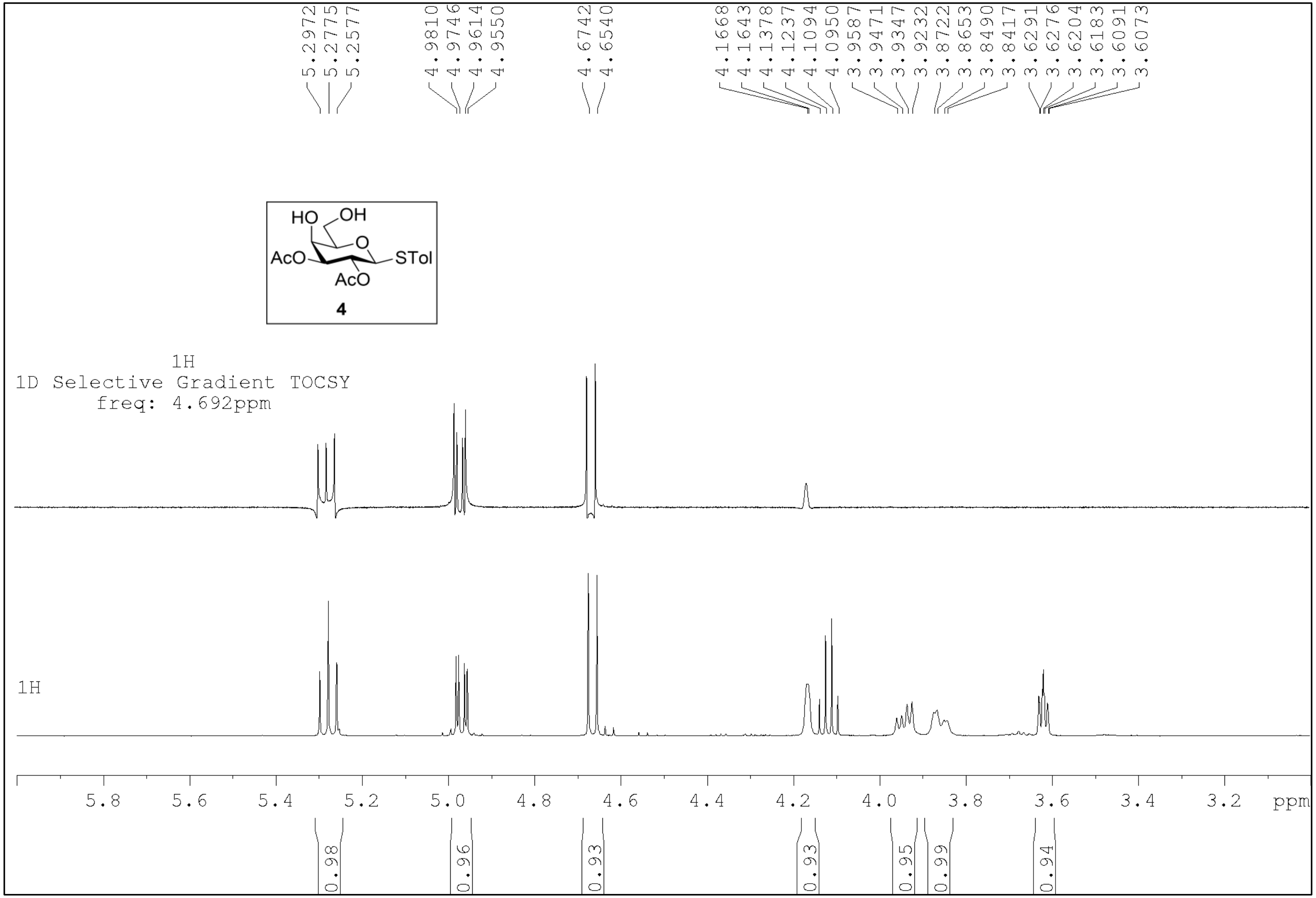

The 1D-TOCSY spectrum in $CDCl_3$ of compound 4.

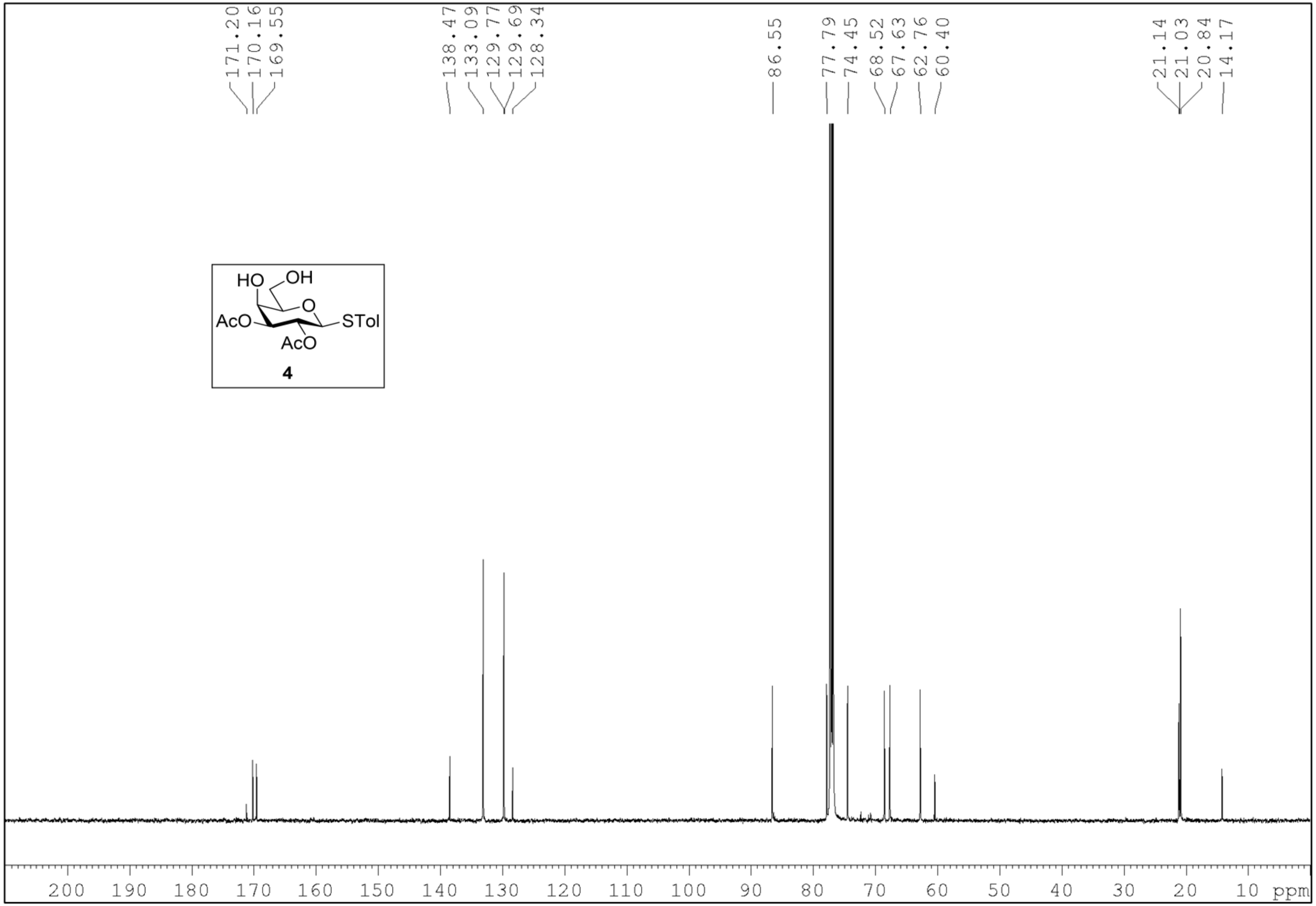

The ^{13}C spectrum in $CDCl_3$ of compound 4.

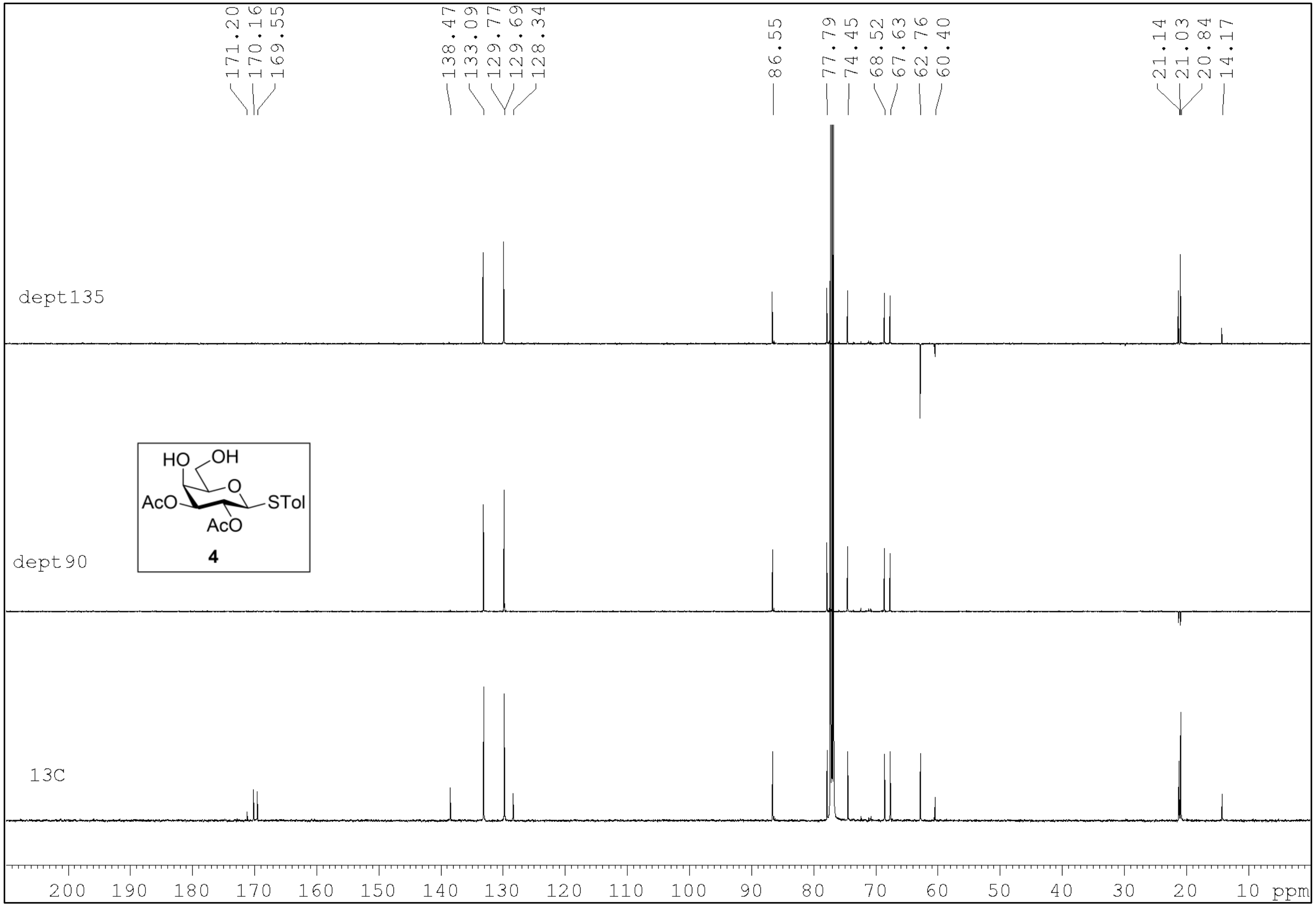

The ^{13}C spectrum in $CDCl_3$ of compound 4.

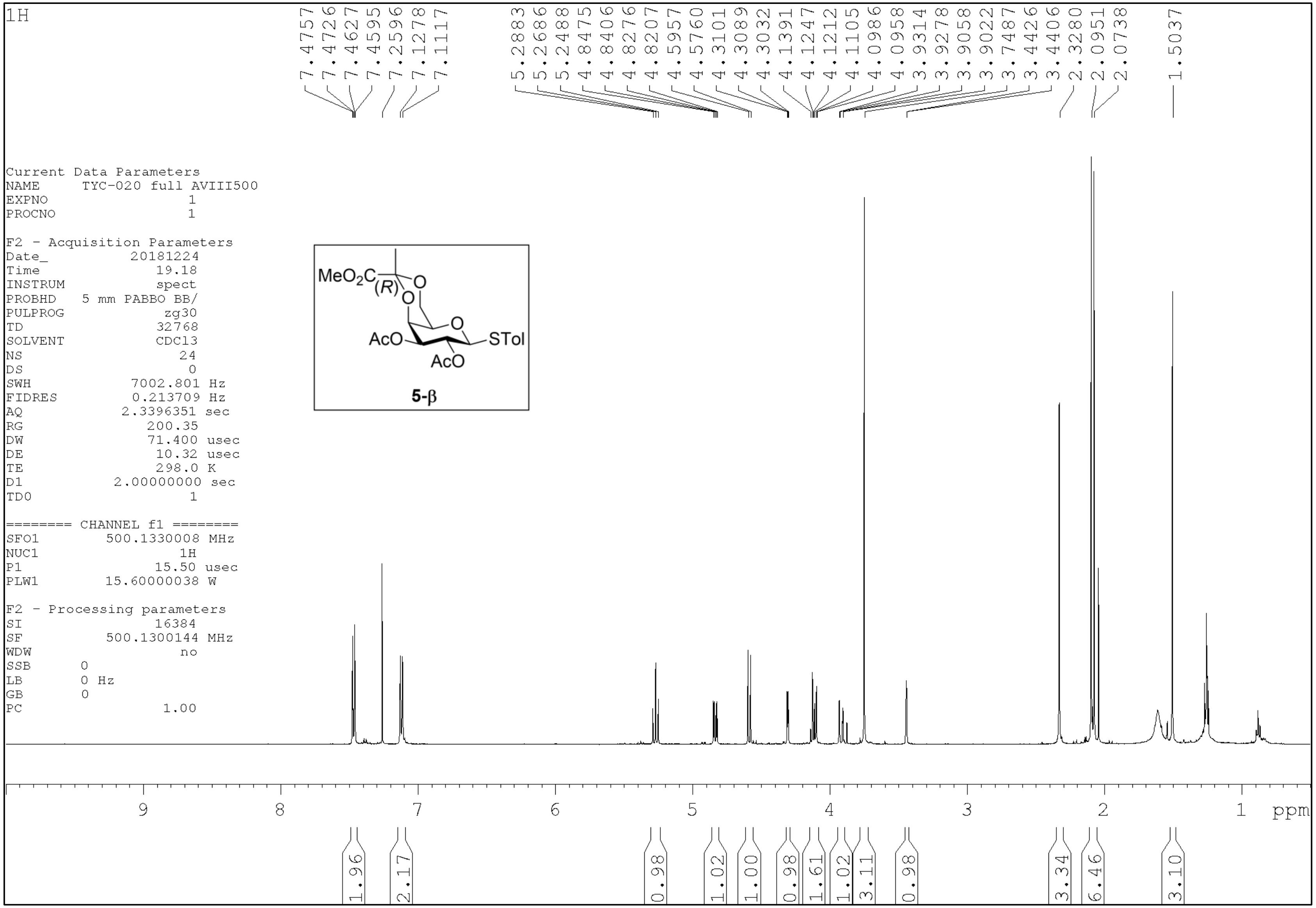

The ^{1}H spectrum in $CDCl_3$ of compound 5-β.

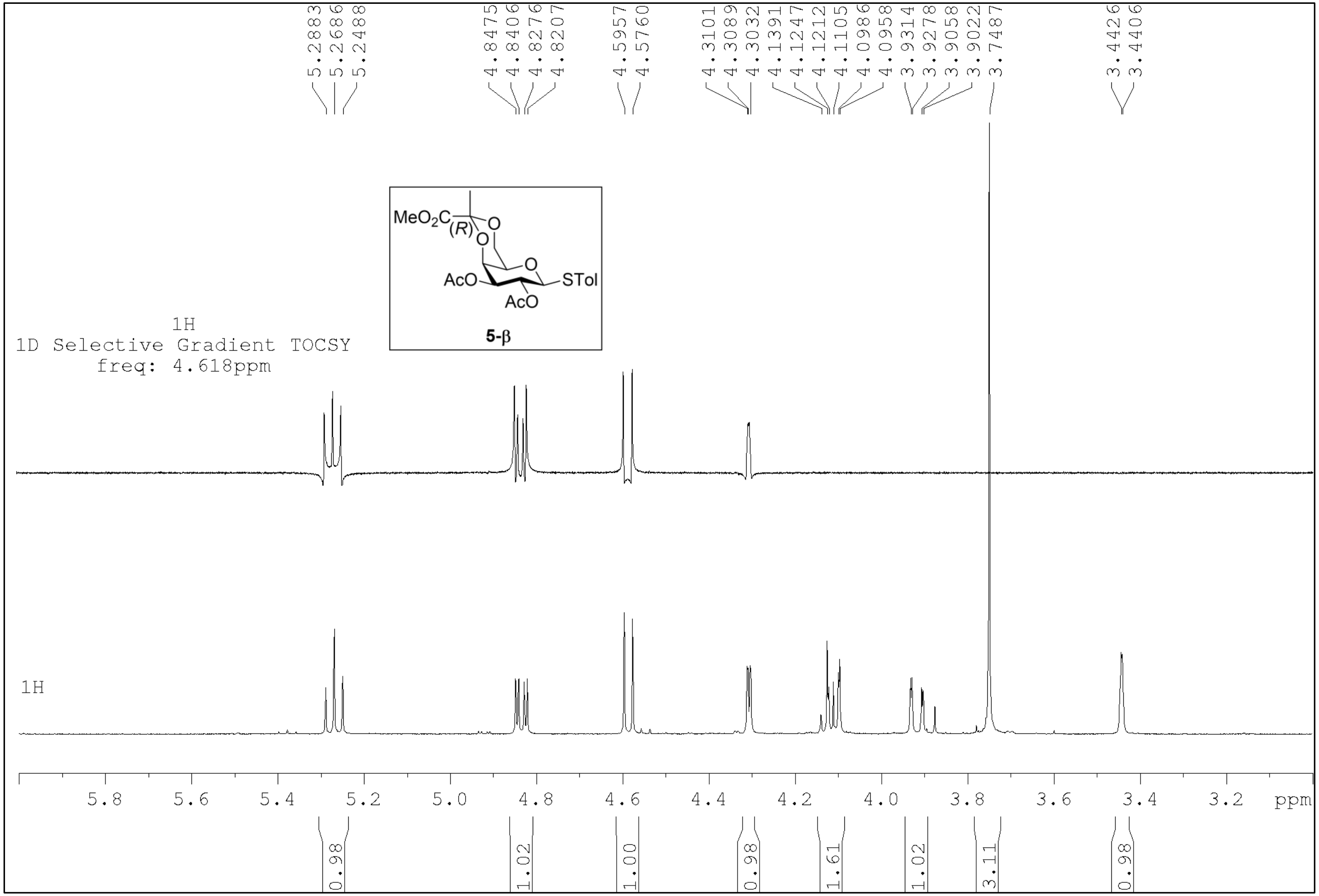

The 1D-TOCSY spectrum in $CDCl_3$ of compound 5-β.

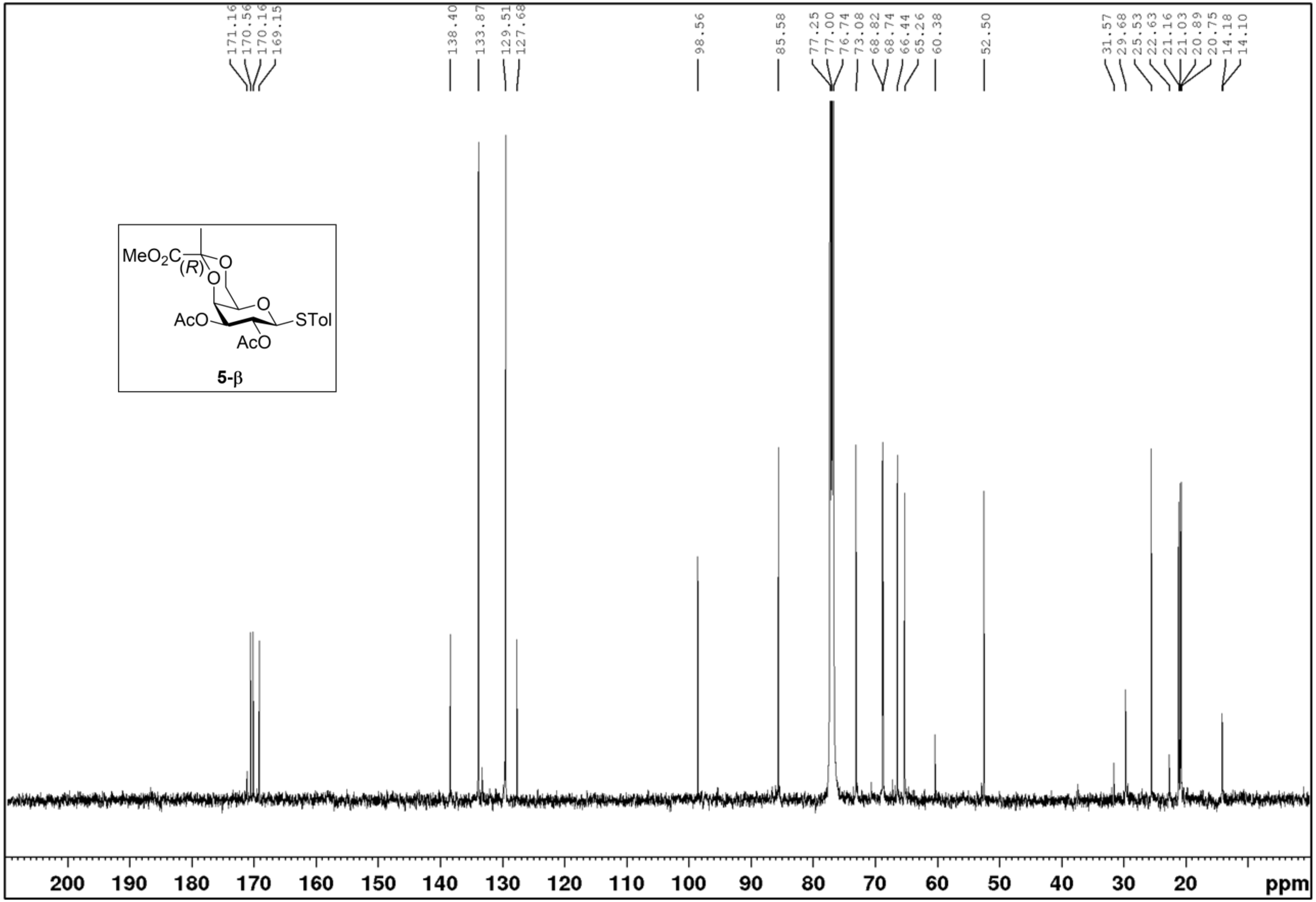

The ^{13}C spectrum in $CDCl_3$ of compound 5-β.

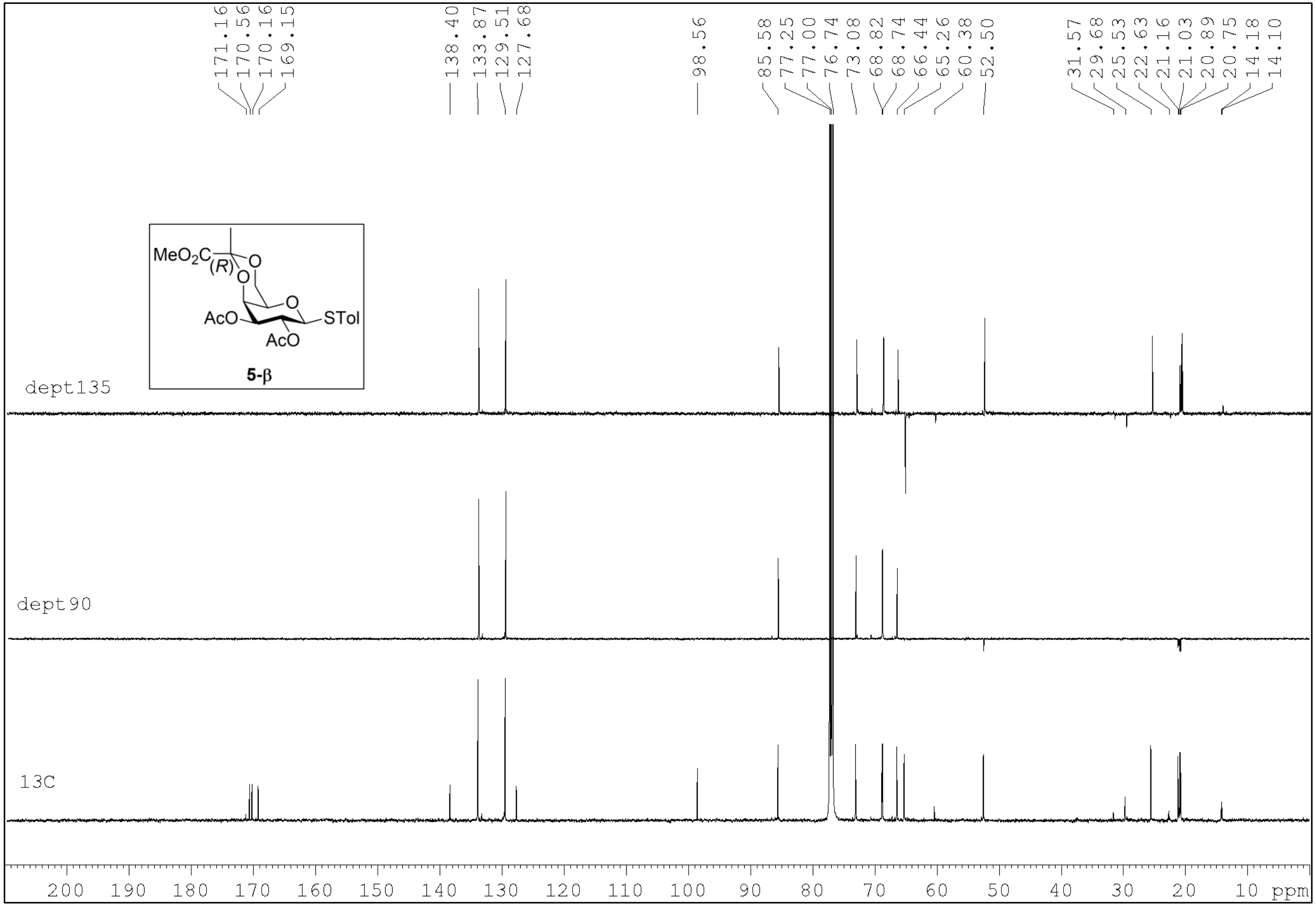

The ^{13}C spectrum in $CDCl_3$ of compound 5-β.

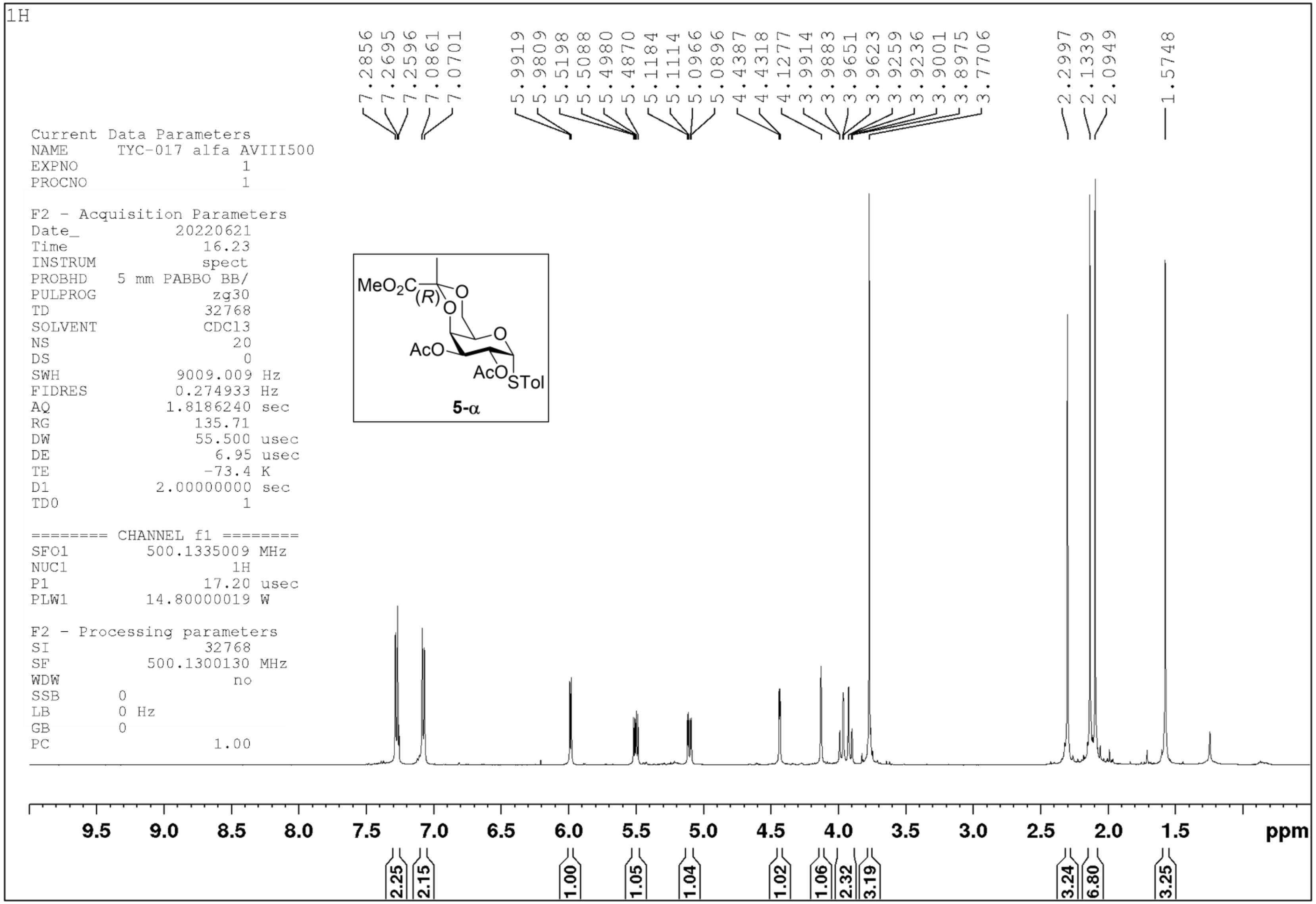

The ^{1}H spectrum in $CDCl_3$ of compound 5-α.

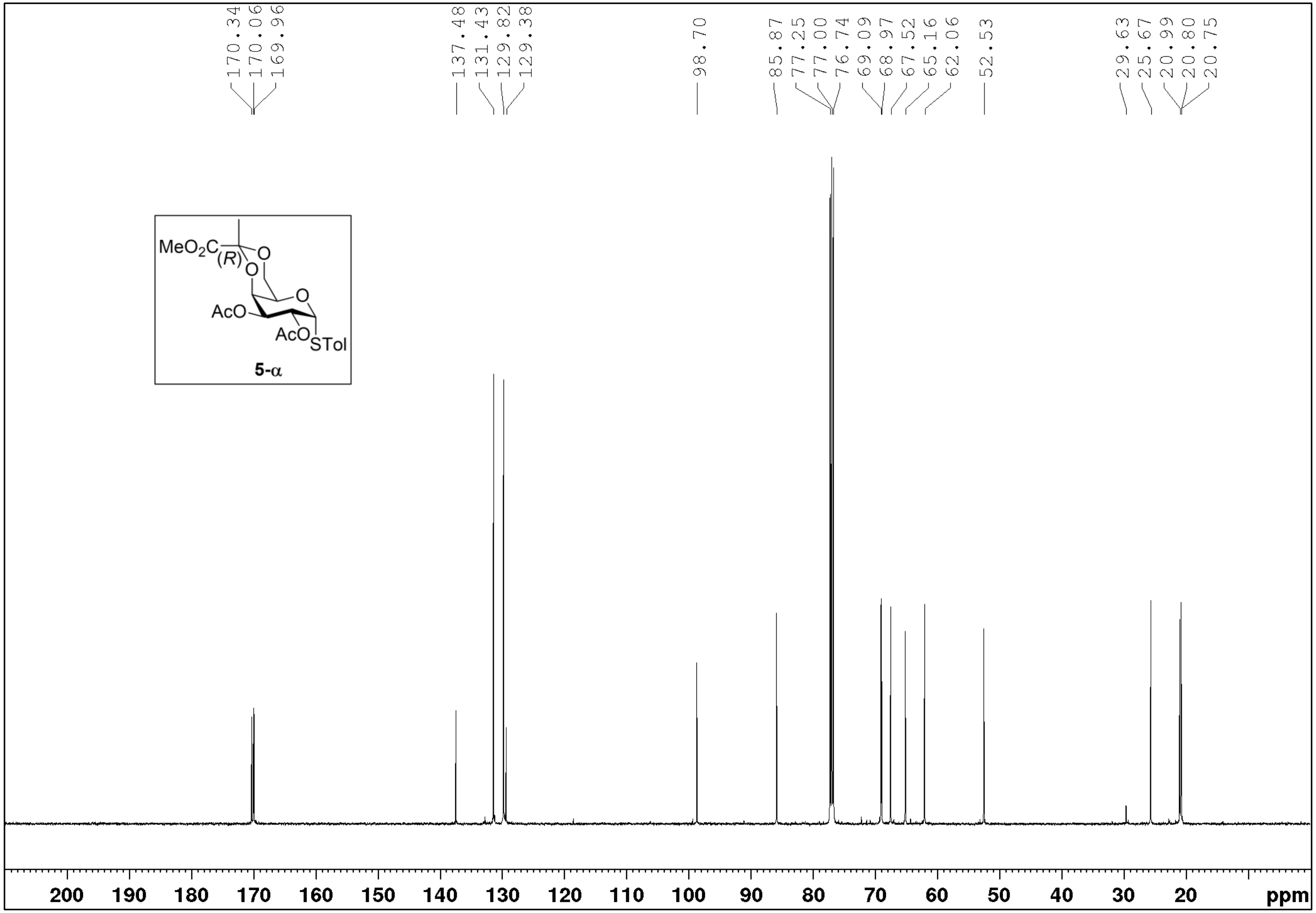

The ^{13}C spectrum in $CDCl_3$ of compound 5-α.

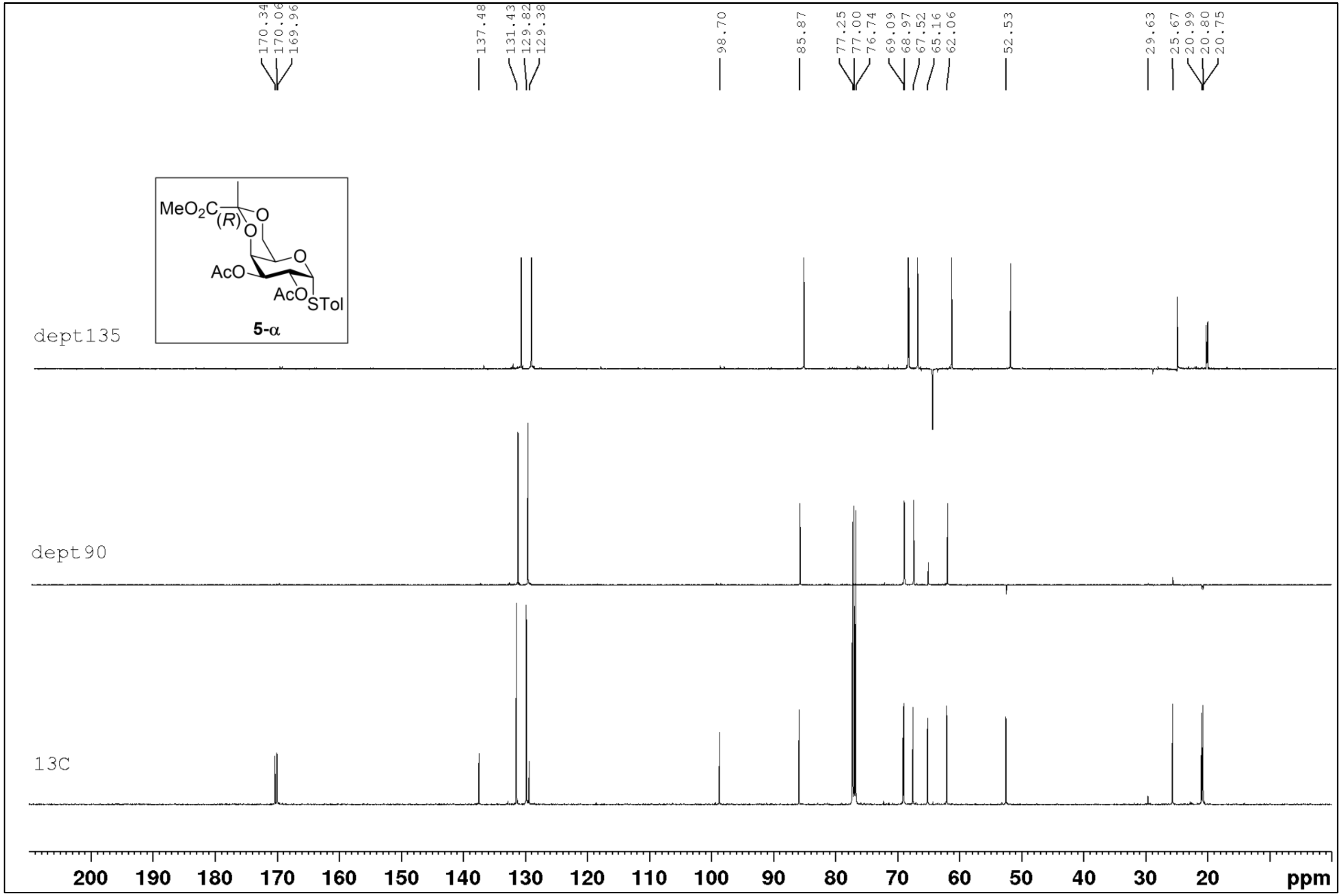

The ^{13}C spectrum in $CDCl_3$ of compound 5-α.

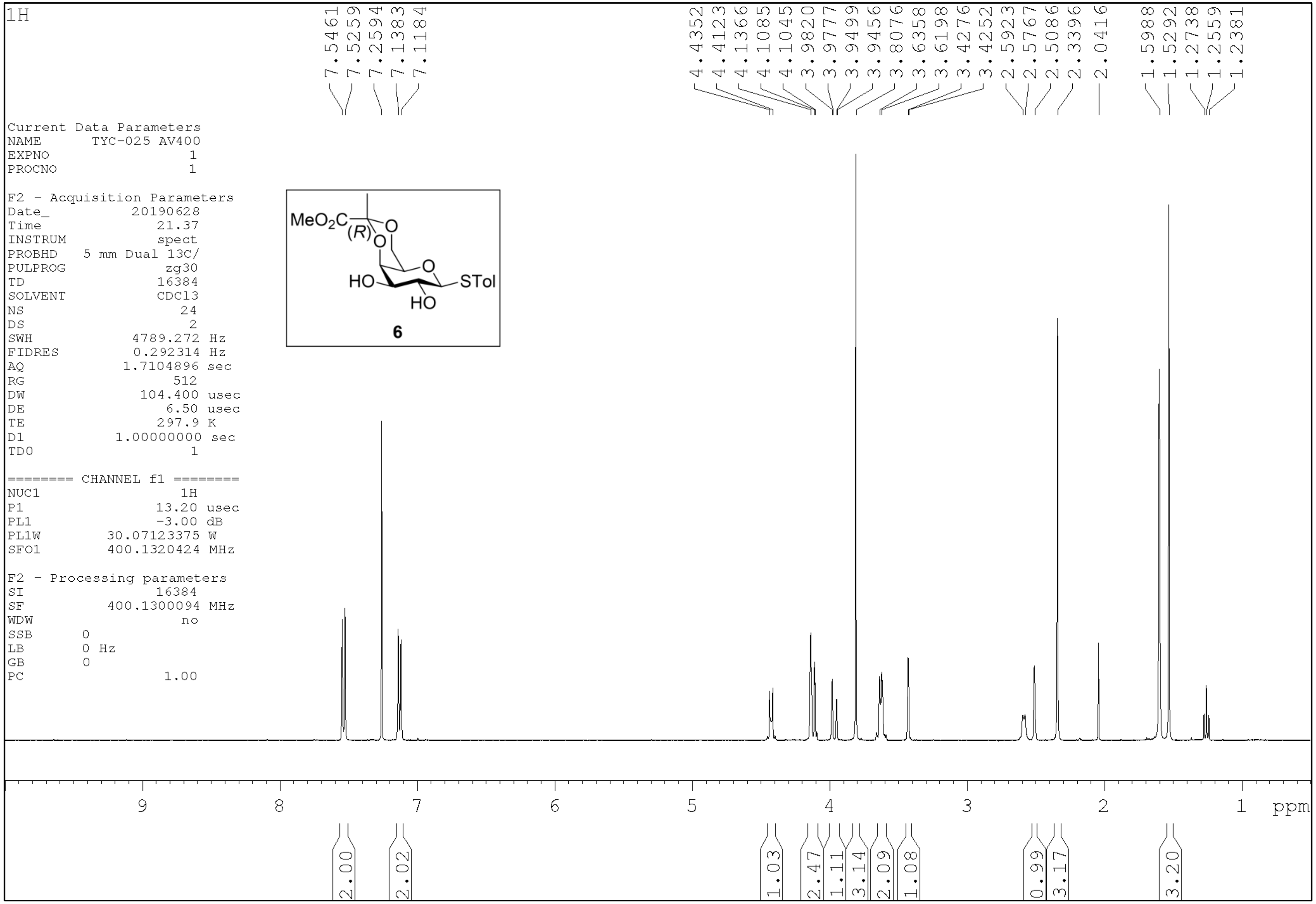

The ^{1}H spectrum in $CDCl_3$ of compound 6.

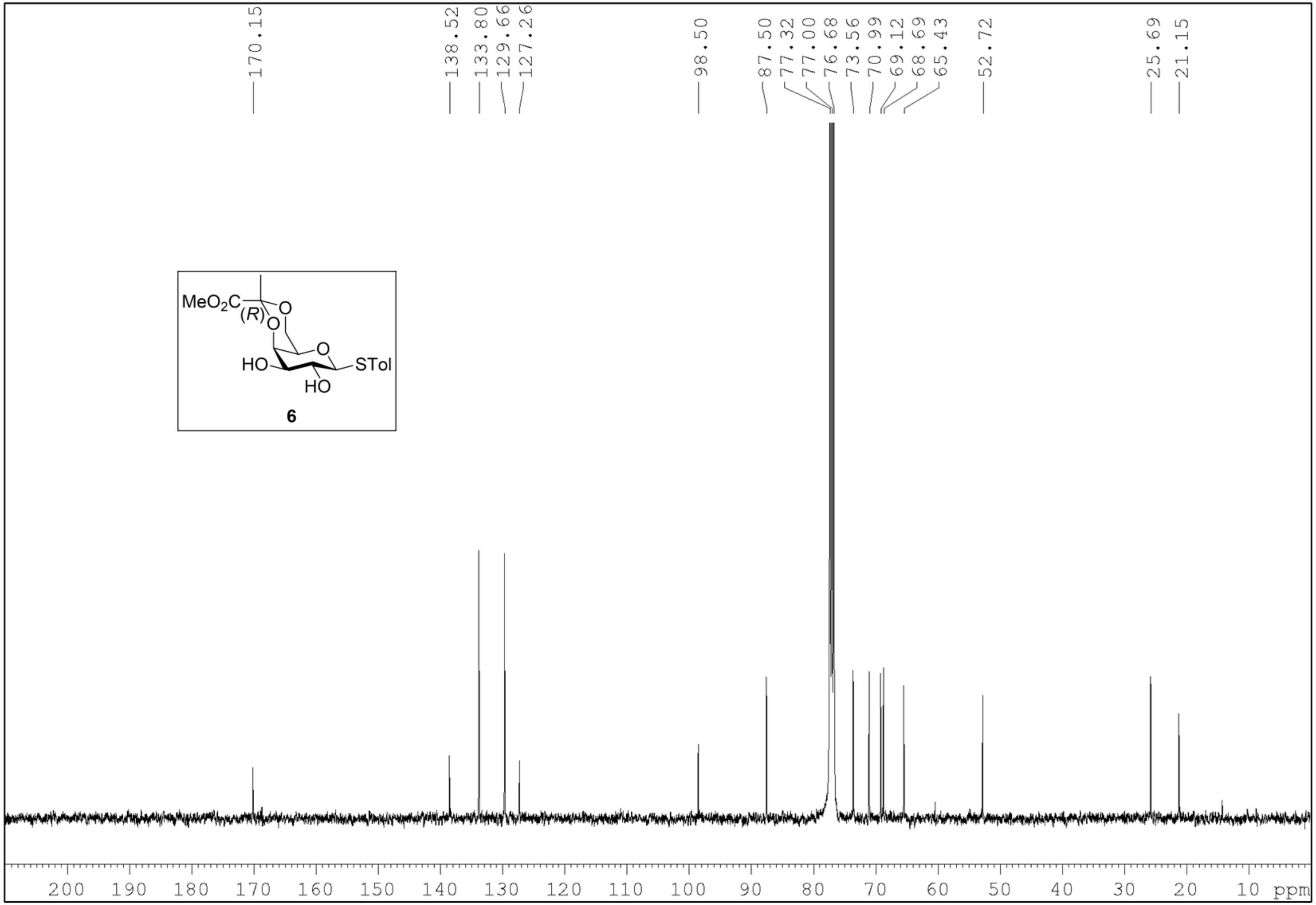

The ^{13}C spectrum in $CDCl_3$ of compound 6.

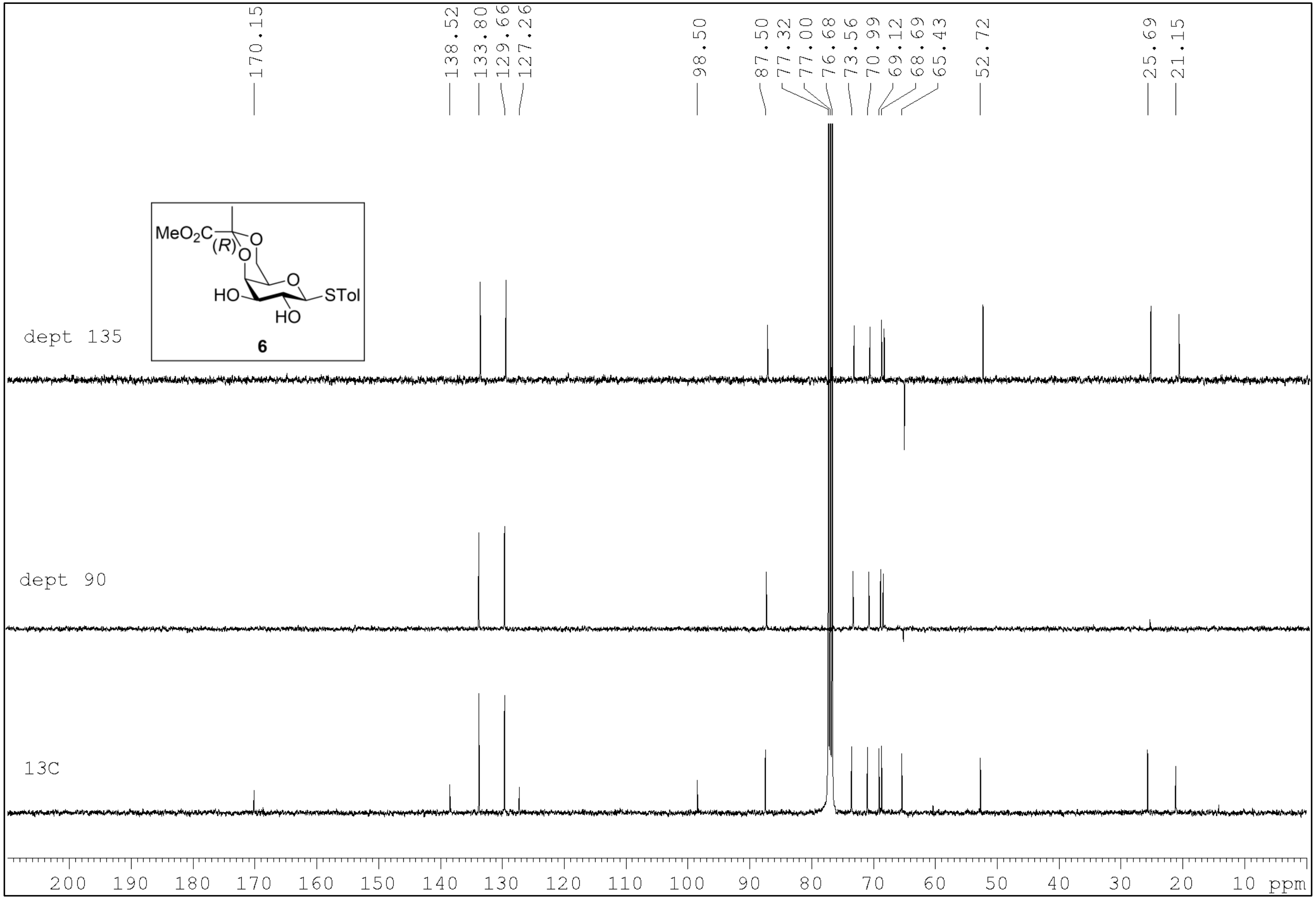

The ^{13}C spectrum in $CDCl_3$ of compound 6.

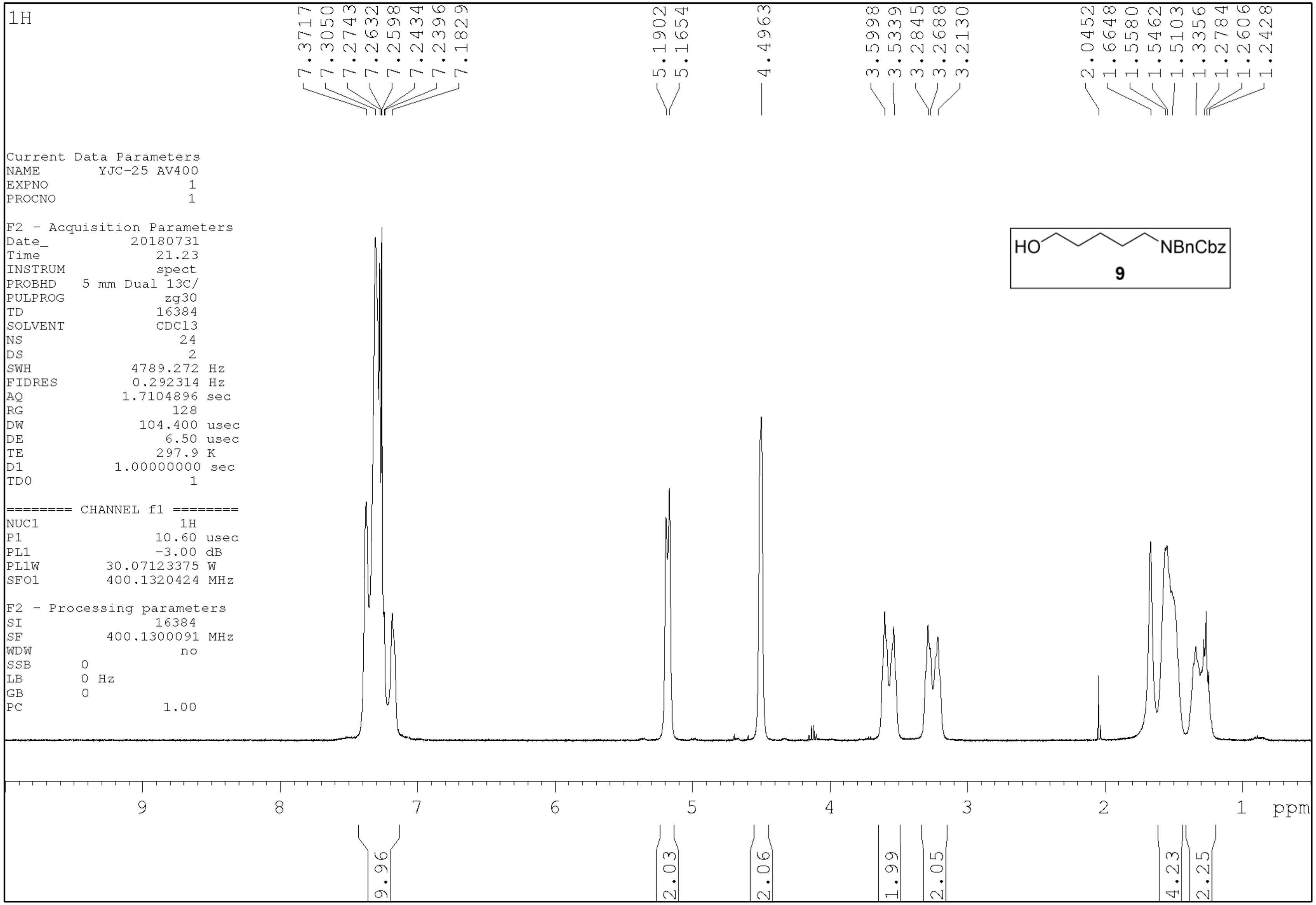

The ^{1}H spectrum in $CDCl_3$ of compound 9.

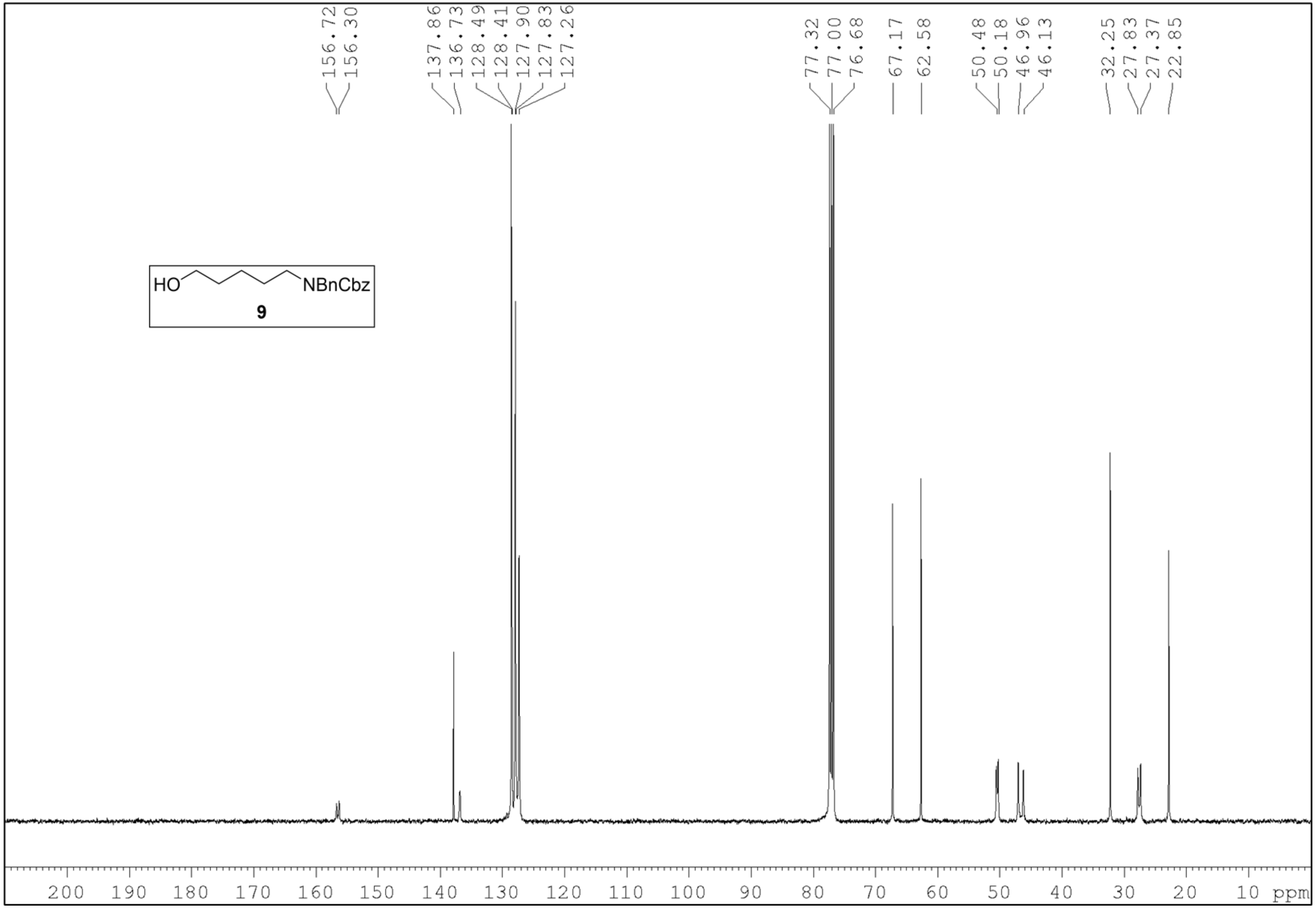

The ^{13}C spectrum in $CDCl_3$ of compound 9.

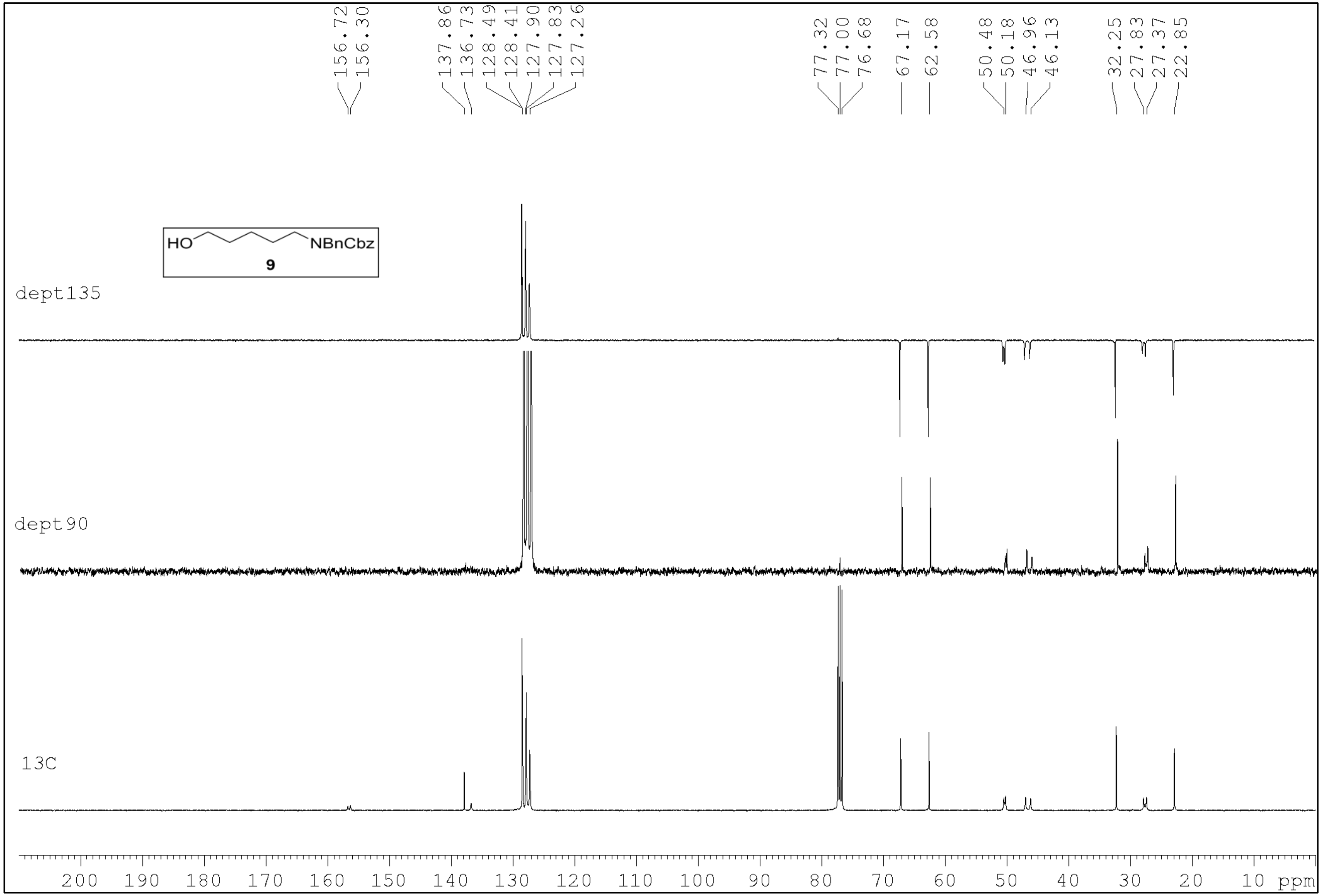

The ^{13}C spectrum in $CDCl_3$ of compound 9.

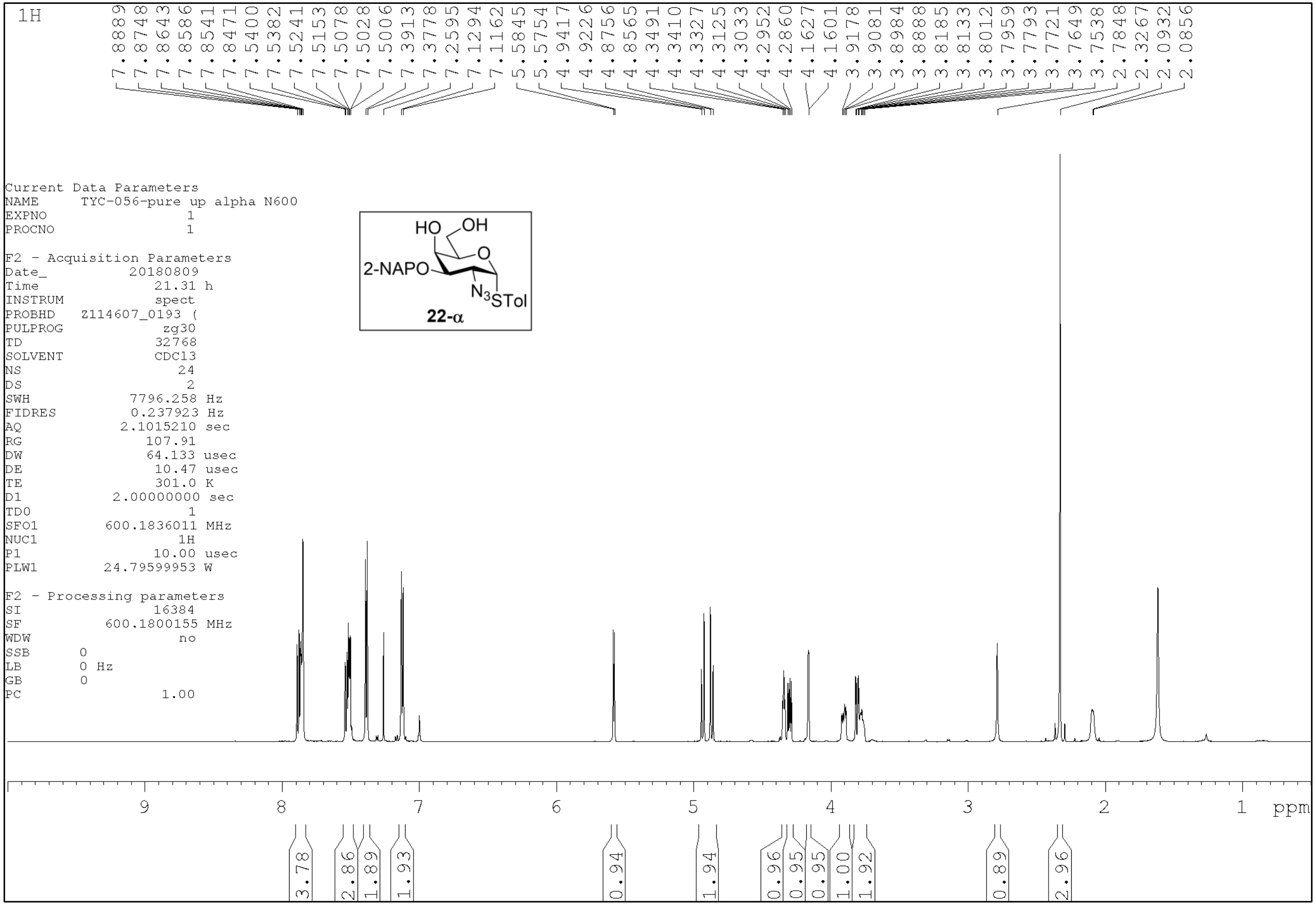

The ^{1}H spectrum in $CDCl_3$ of compound 22-α.

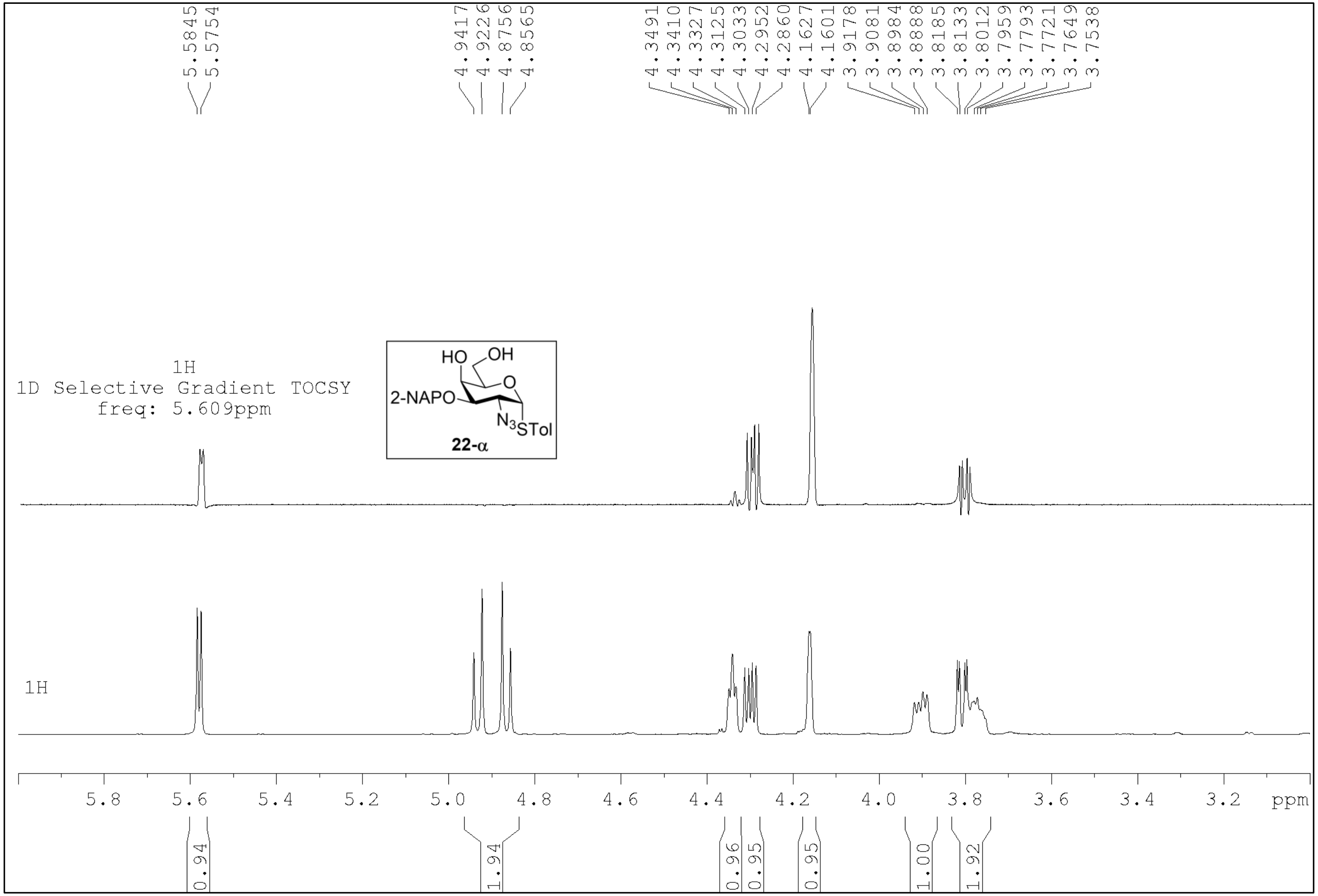

The 1D-TOCSY spectrum in $CDCl_3$ of compound 22-α.

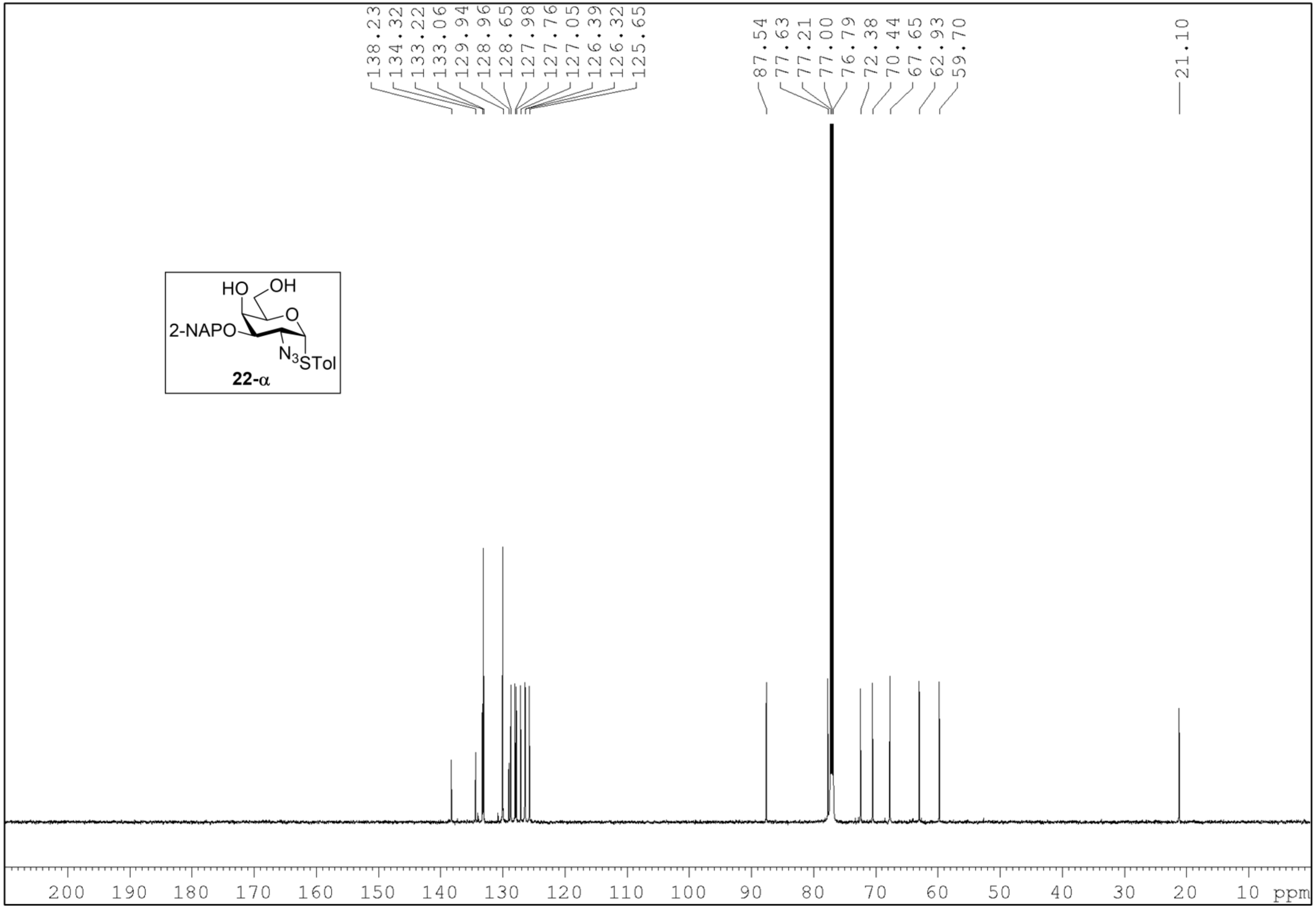

The ^{13}C spectrum in $CDCl_3$ of compound 22-α.

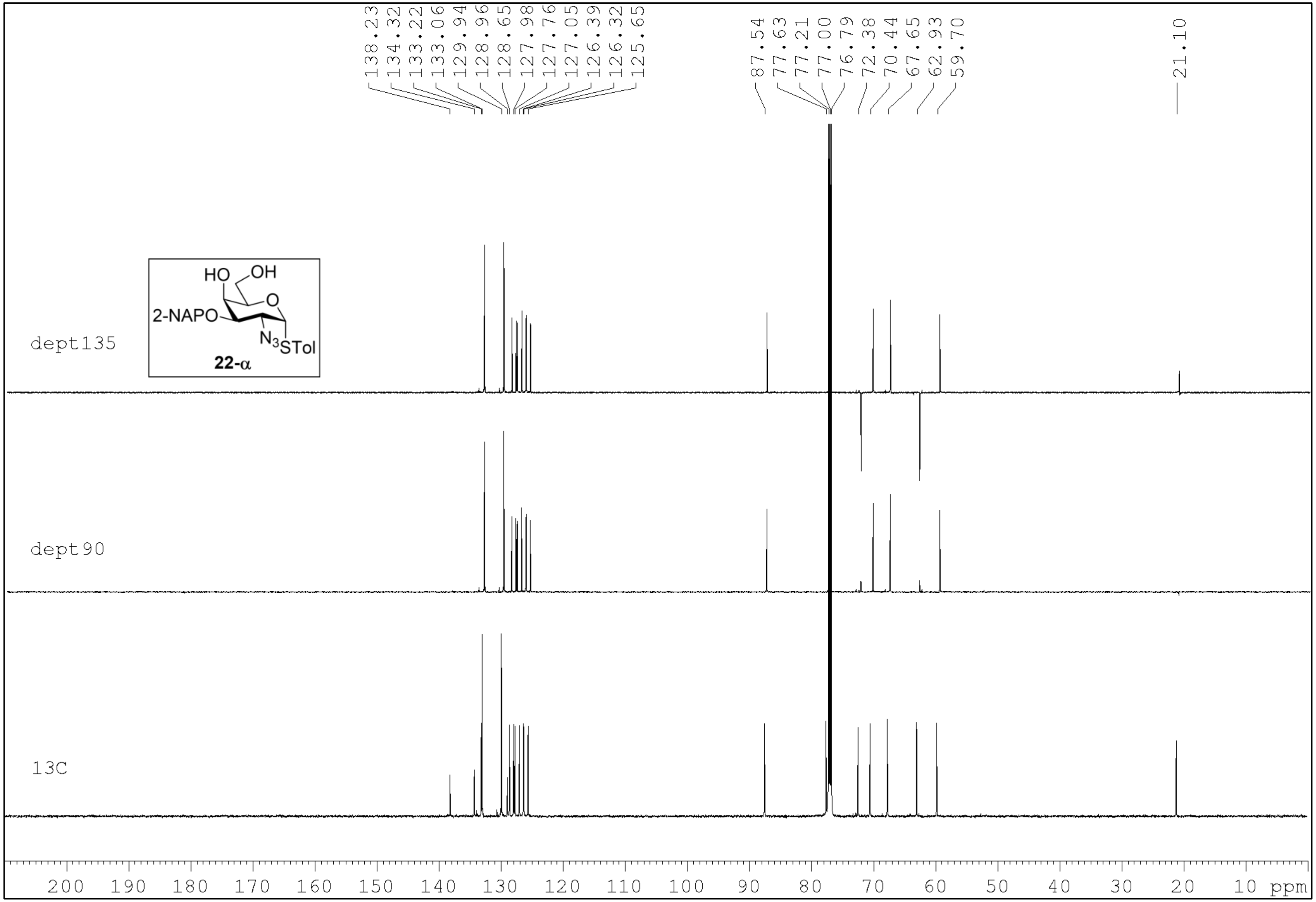

The ^{13}C spectrum in $CDCl_3$ of compound 22-α.

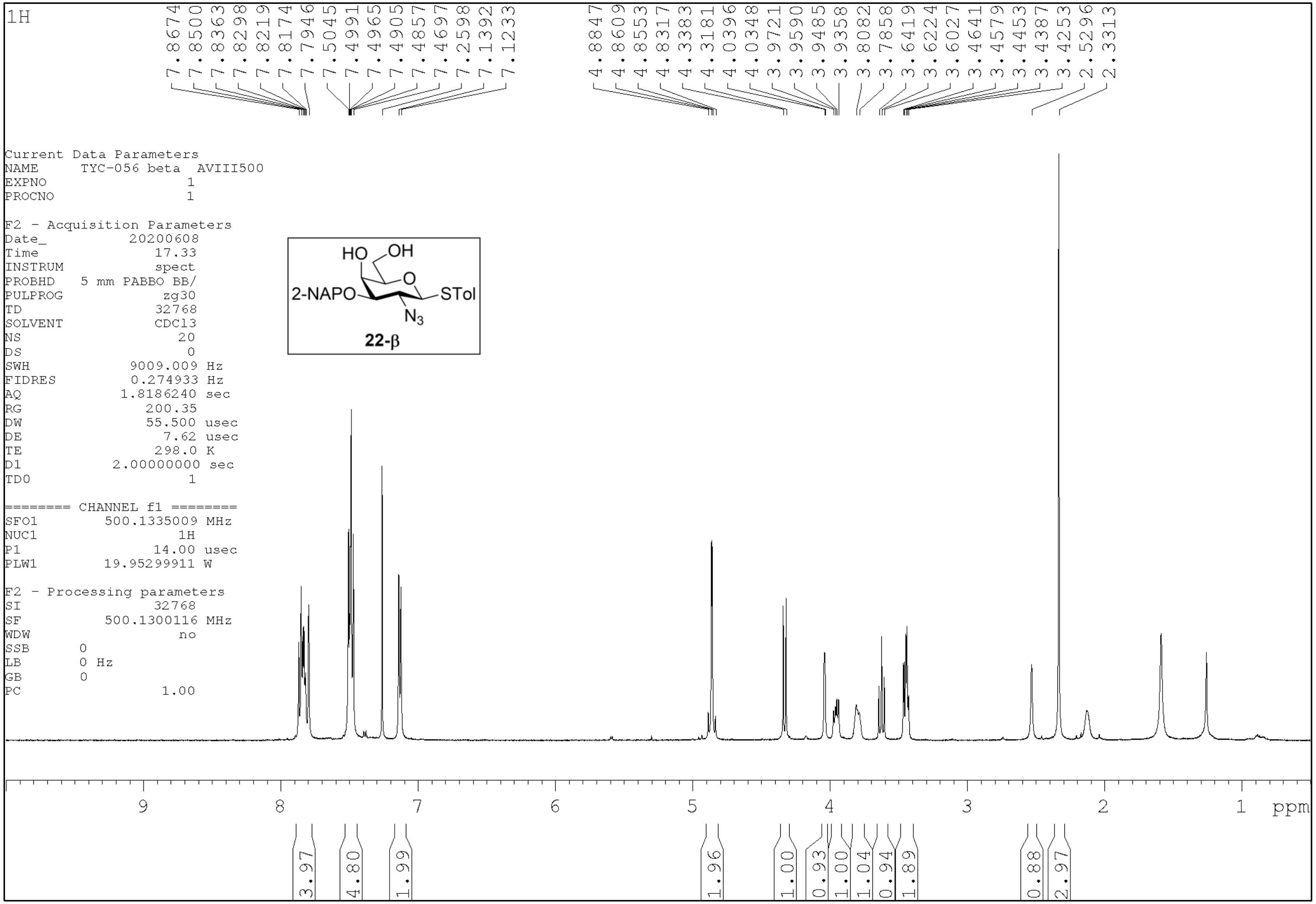

The ^{1}H spectrum in $CDCl_3$ of compound 22-β.

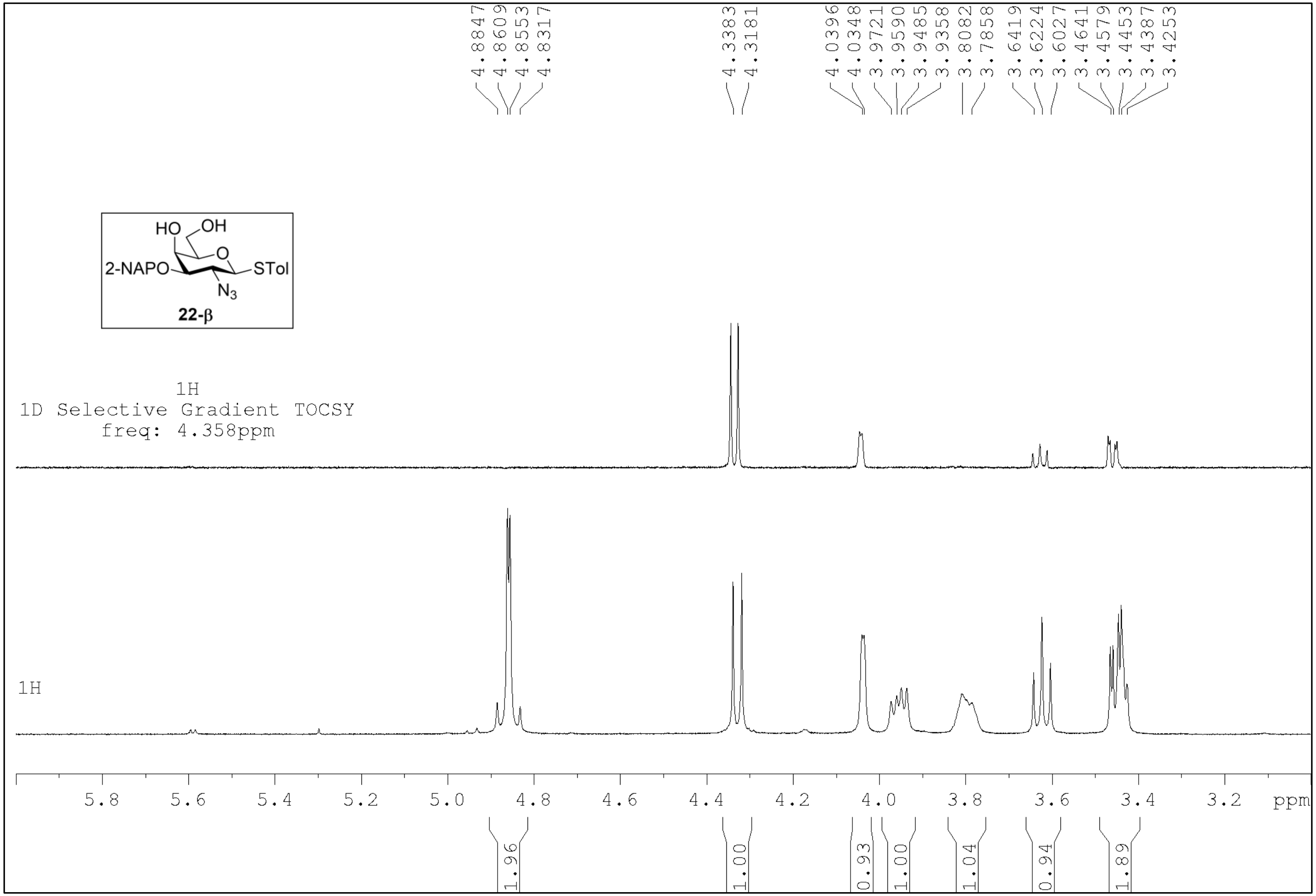

The 1D-TOCSY spectrum in $CDCl_3$ of compound 22-β.

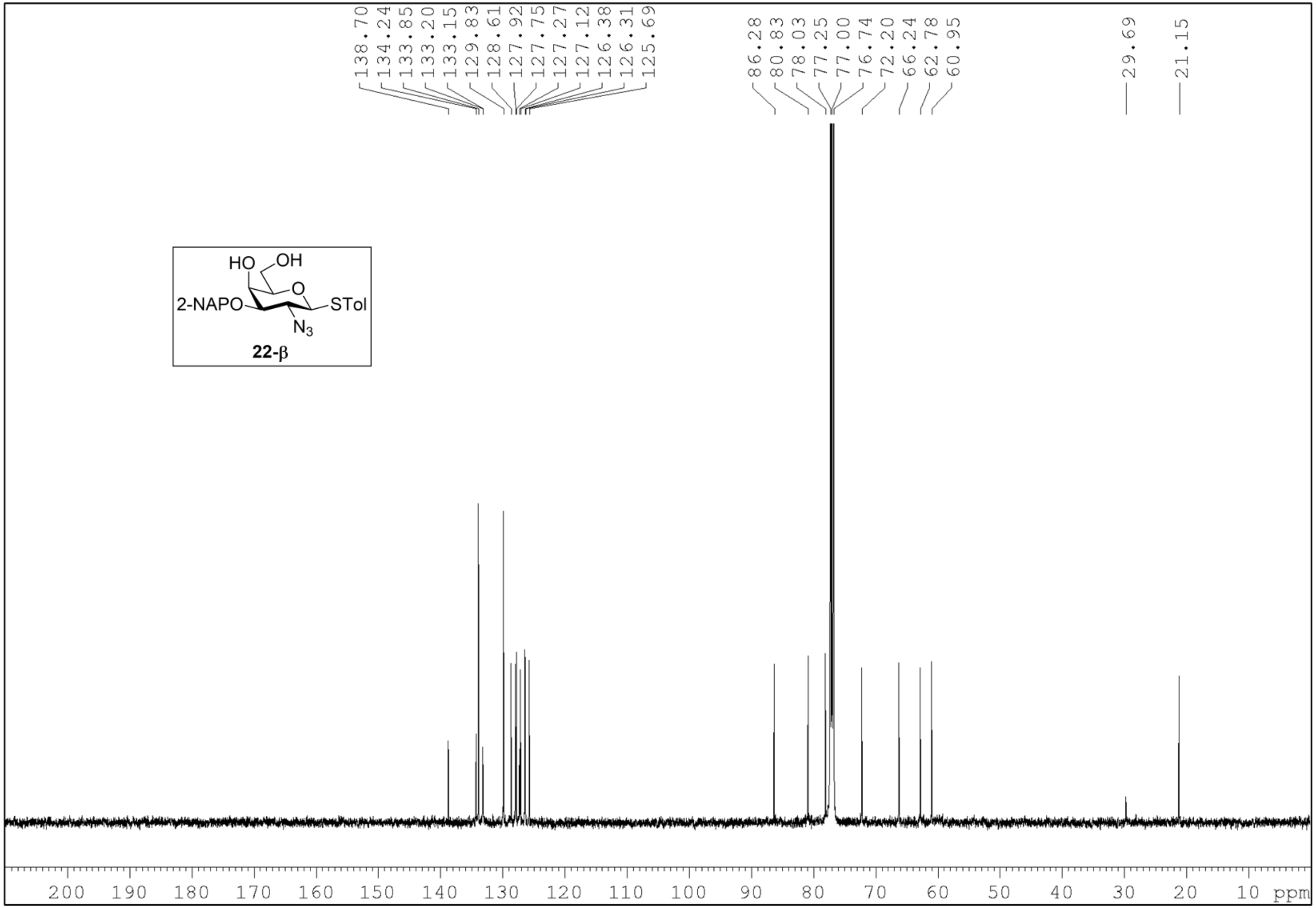

The ^{13}C spectrum in $CDCl_3$ of compound 22-β.

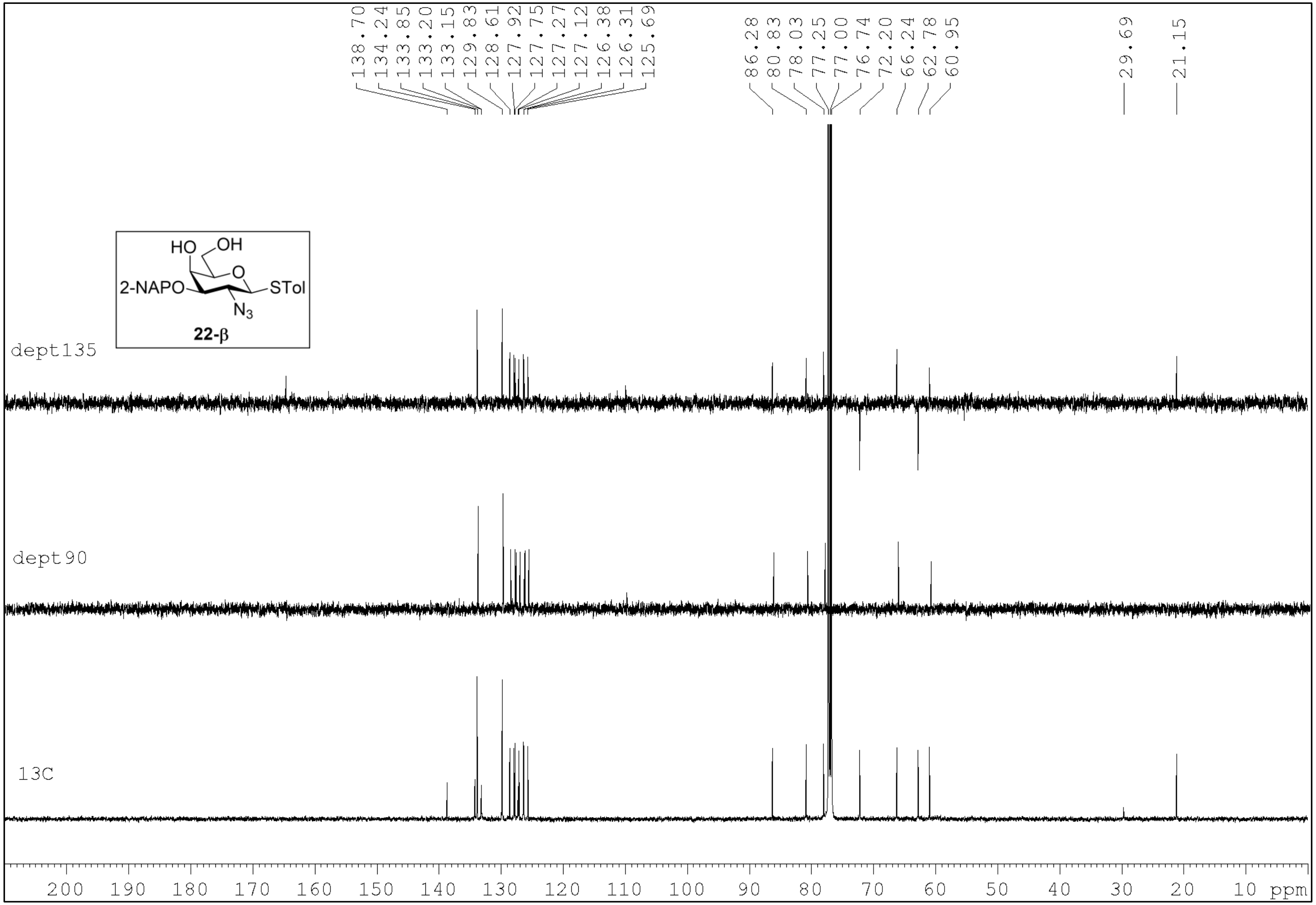

The ^{13}C spectrum in $CDCl_3$ of compound 22-β.

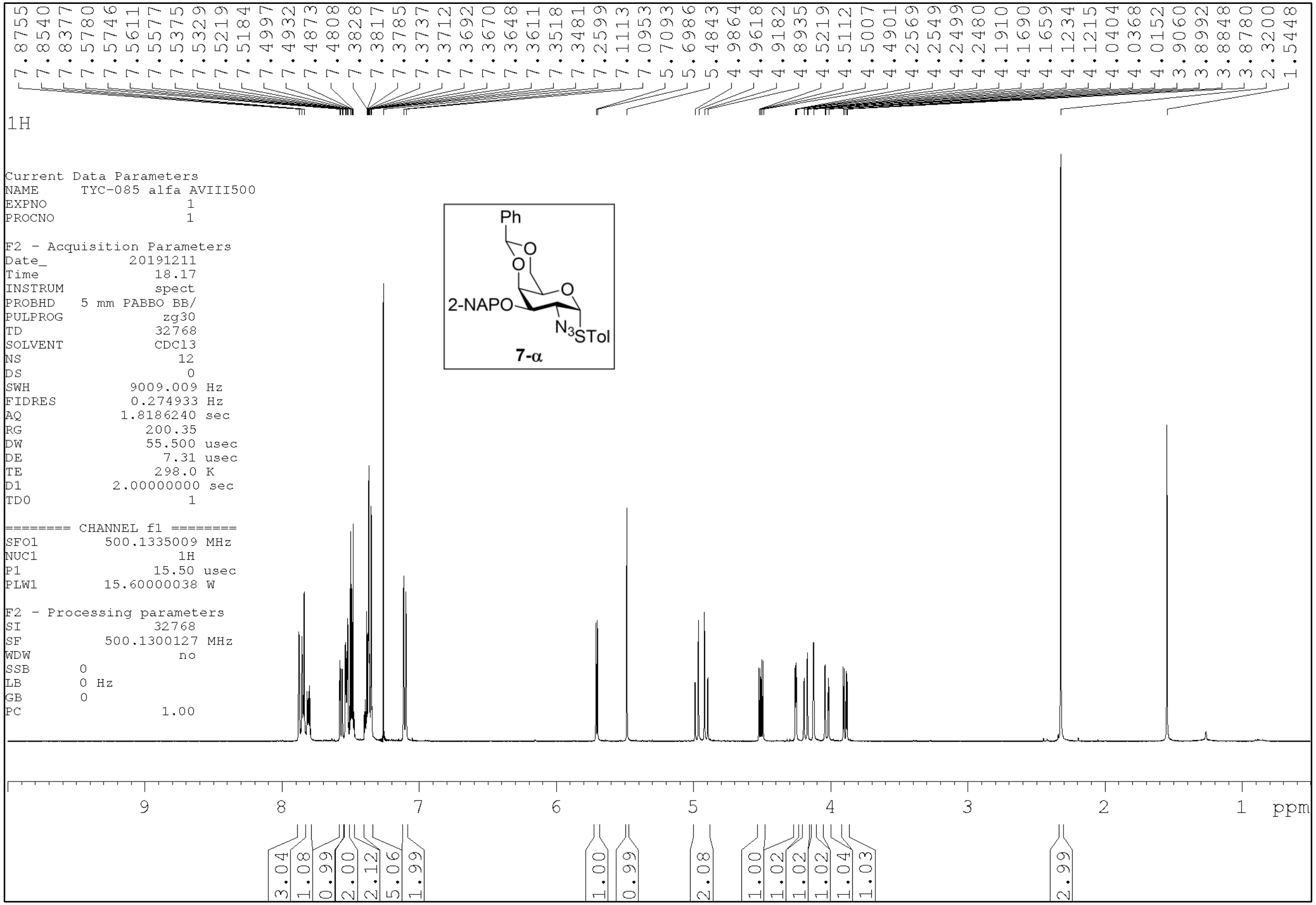

The ^{1}H spectrum in $CDCl_3$ of compound 7-α.

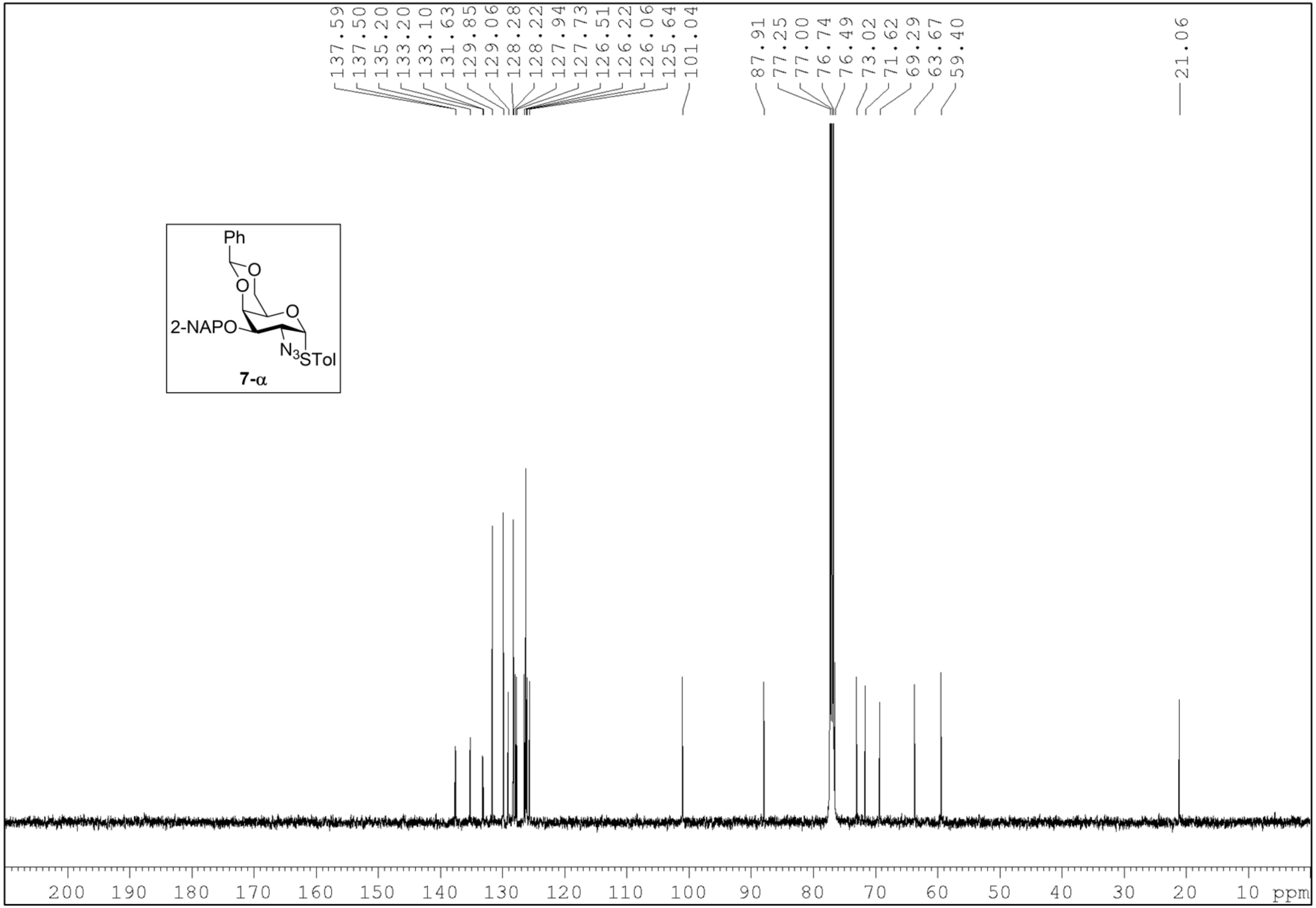

The ^{13}C spectrum in $CDCl_3$ of compound 7-α.

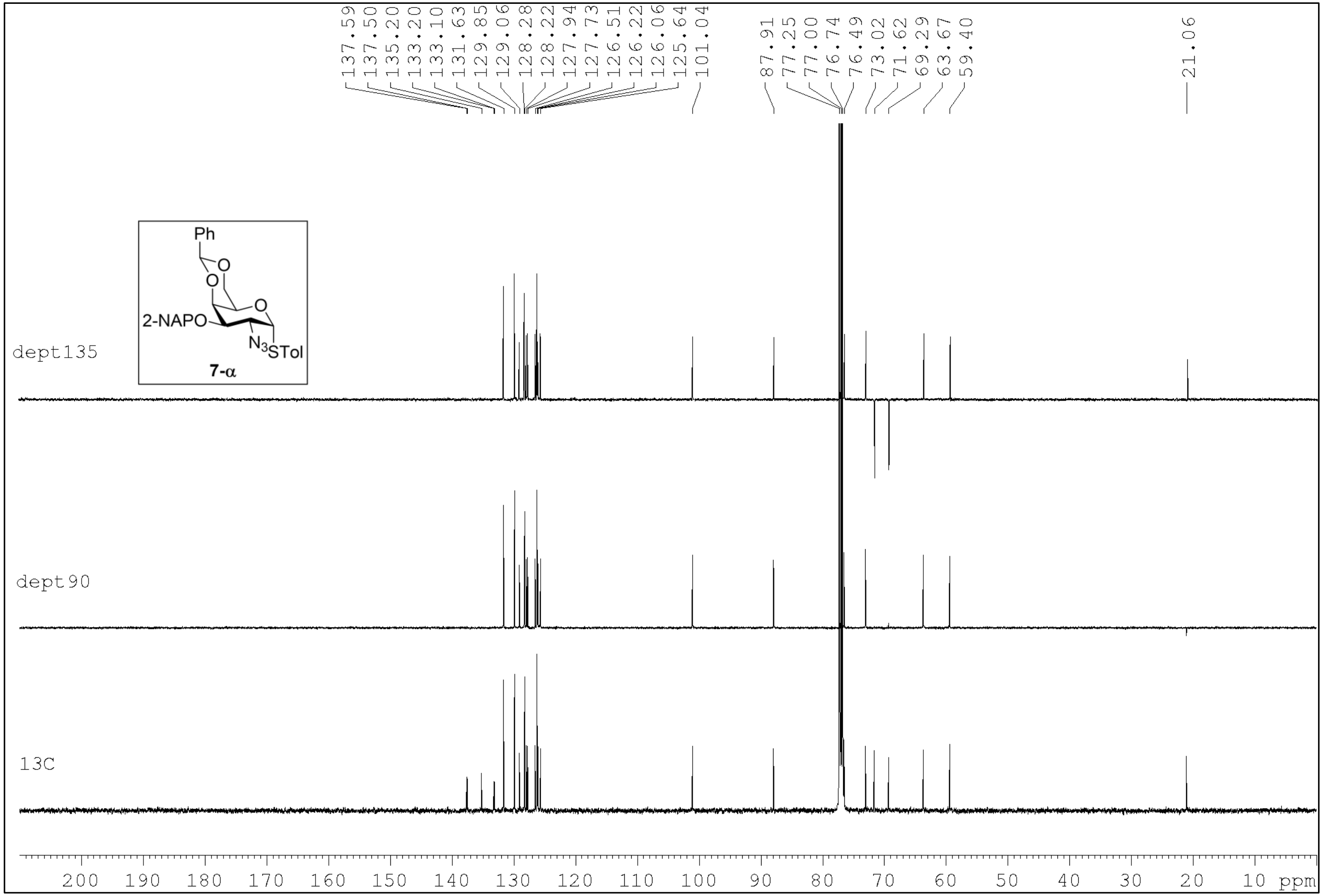

The ^{13}C spectrum in $CDCl_3$ of compound 7-α.

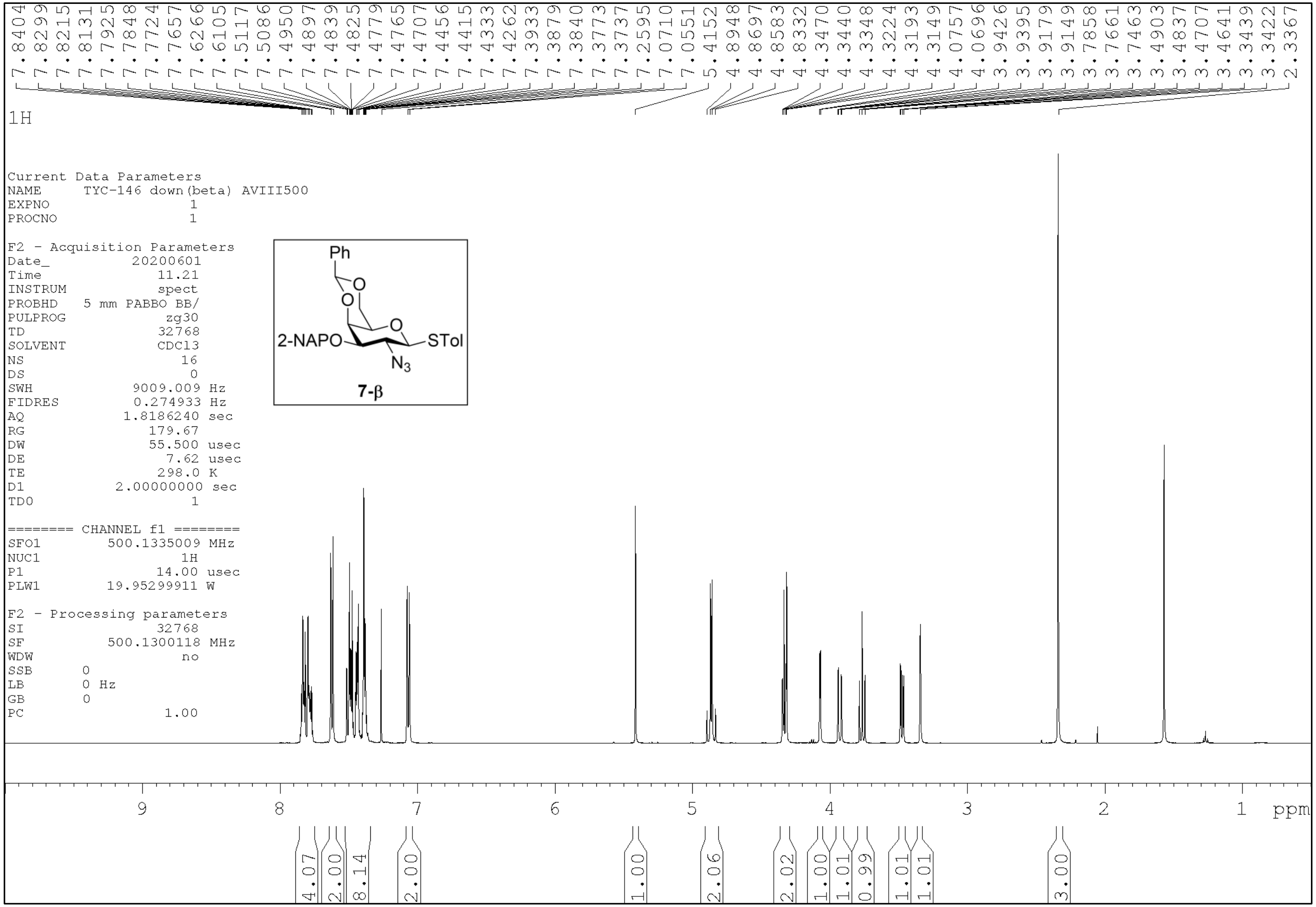

The ^{1}H spectrum in $CDCl_3$ of compound 7-β.

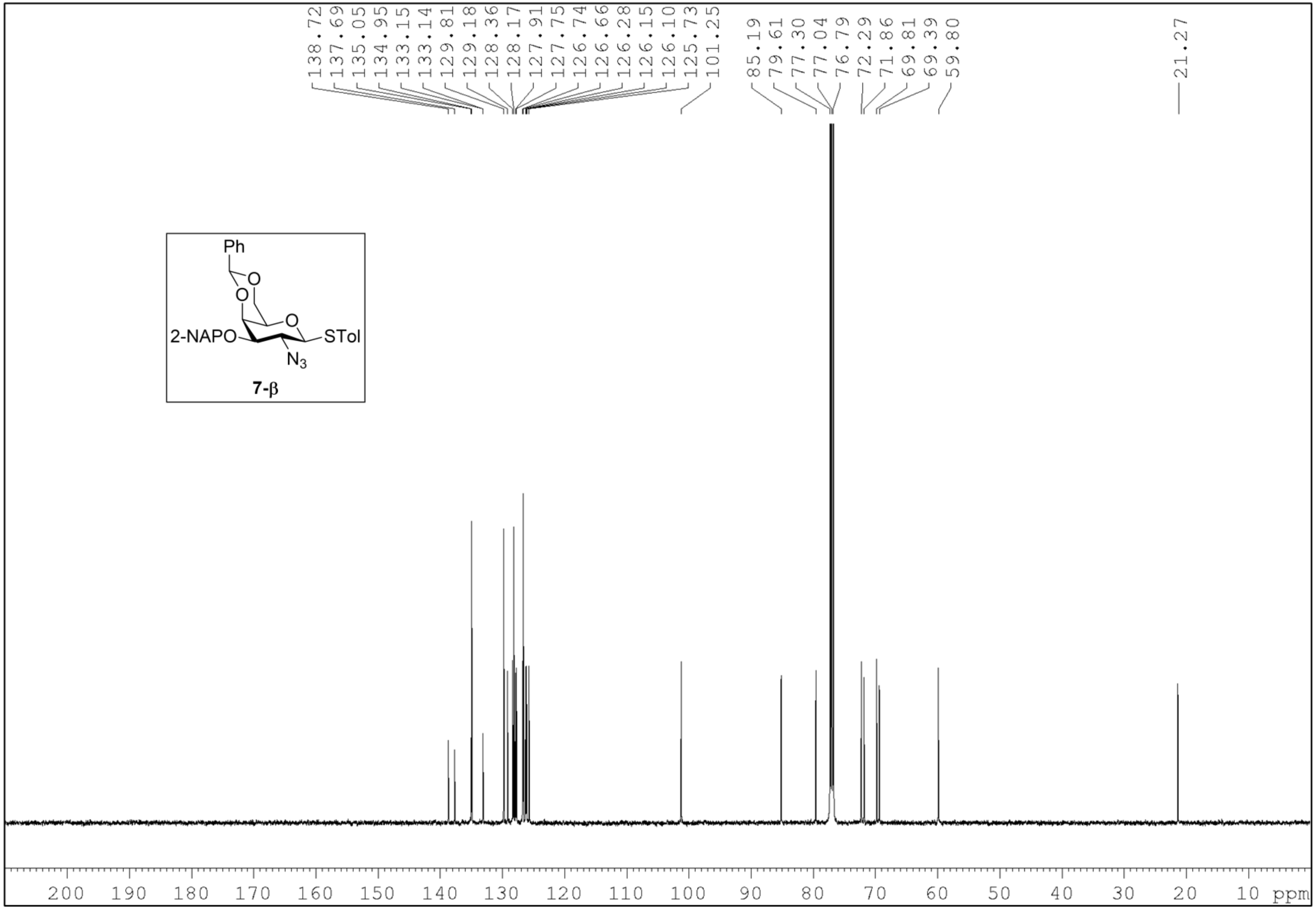

The ^{13}C spectrum in $CDCl_3$ of compound 7-β.

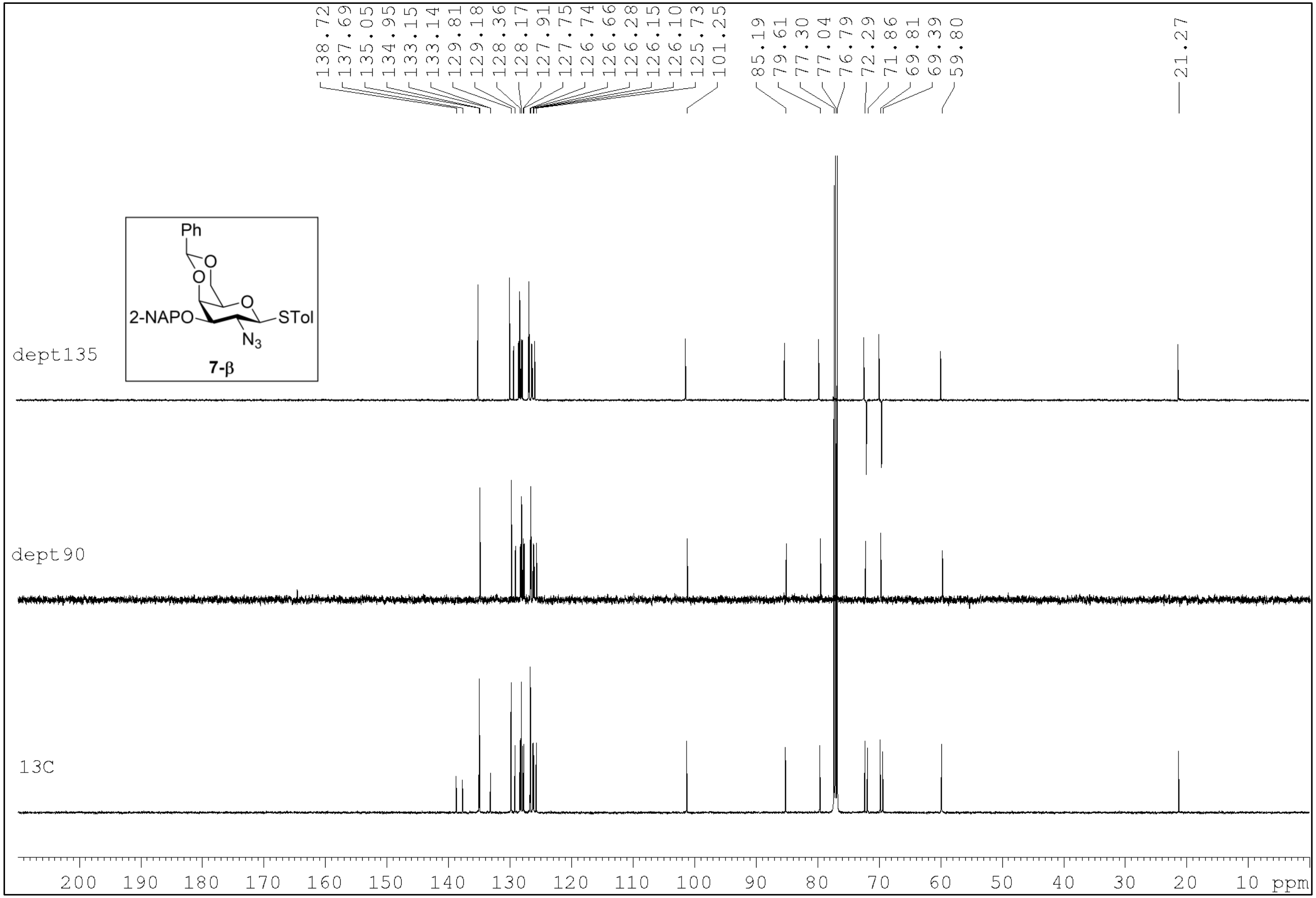

The ^{13}C spectrum in $CDCl_3$ of compound 7-β.

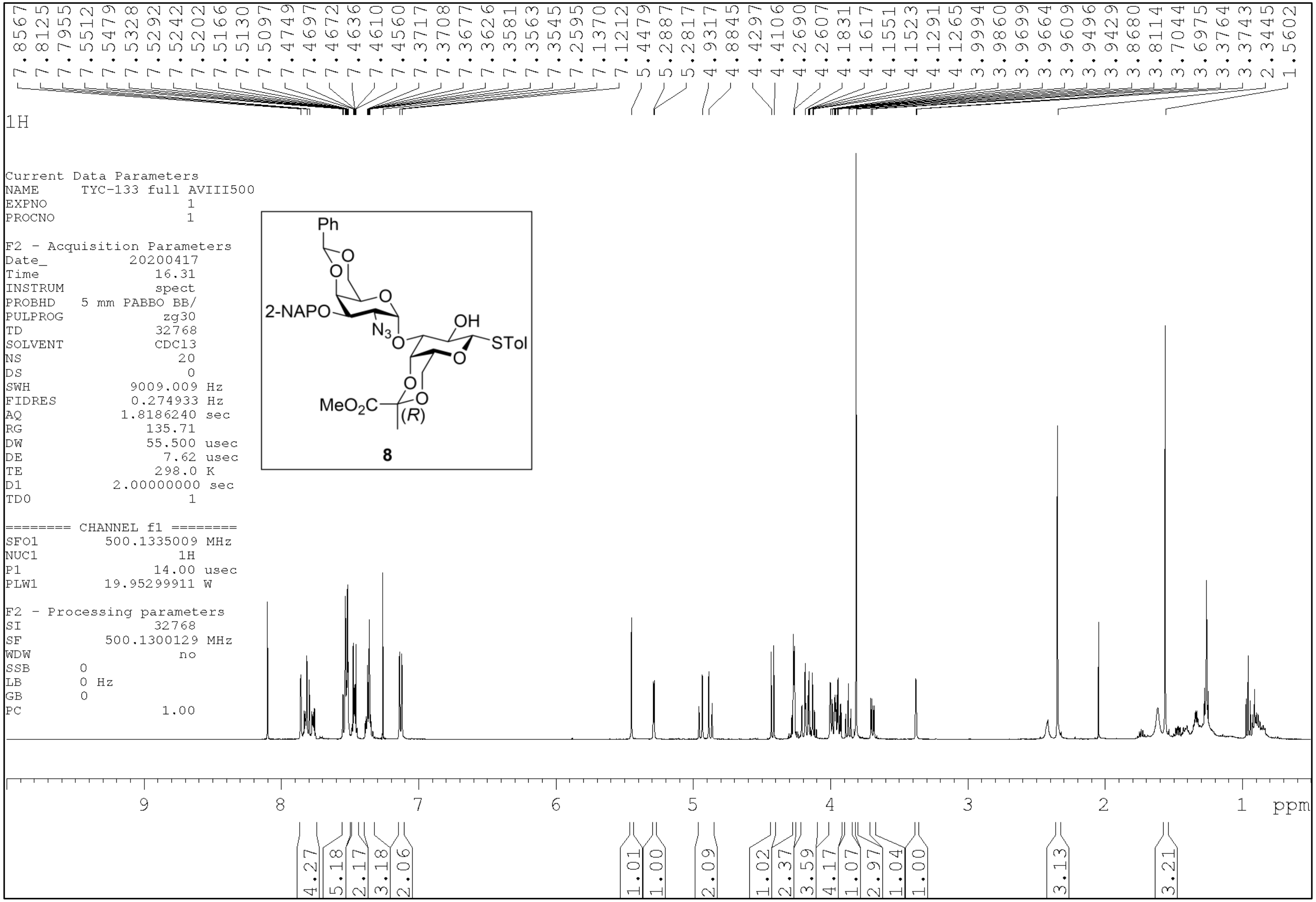

The ^1H spectrum in $CDCl_3$ of compound 8.

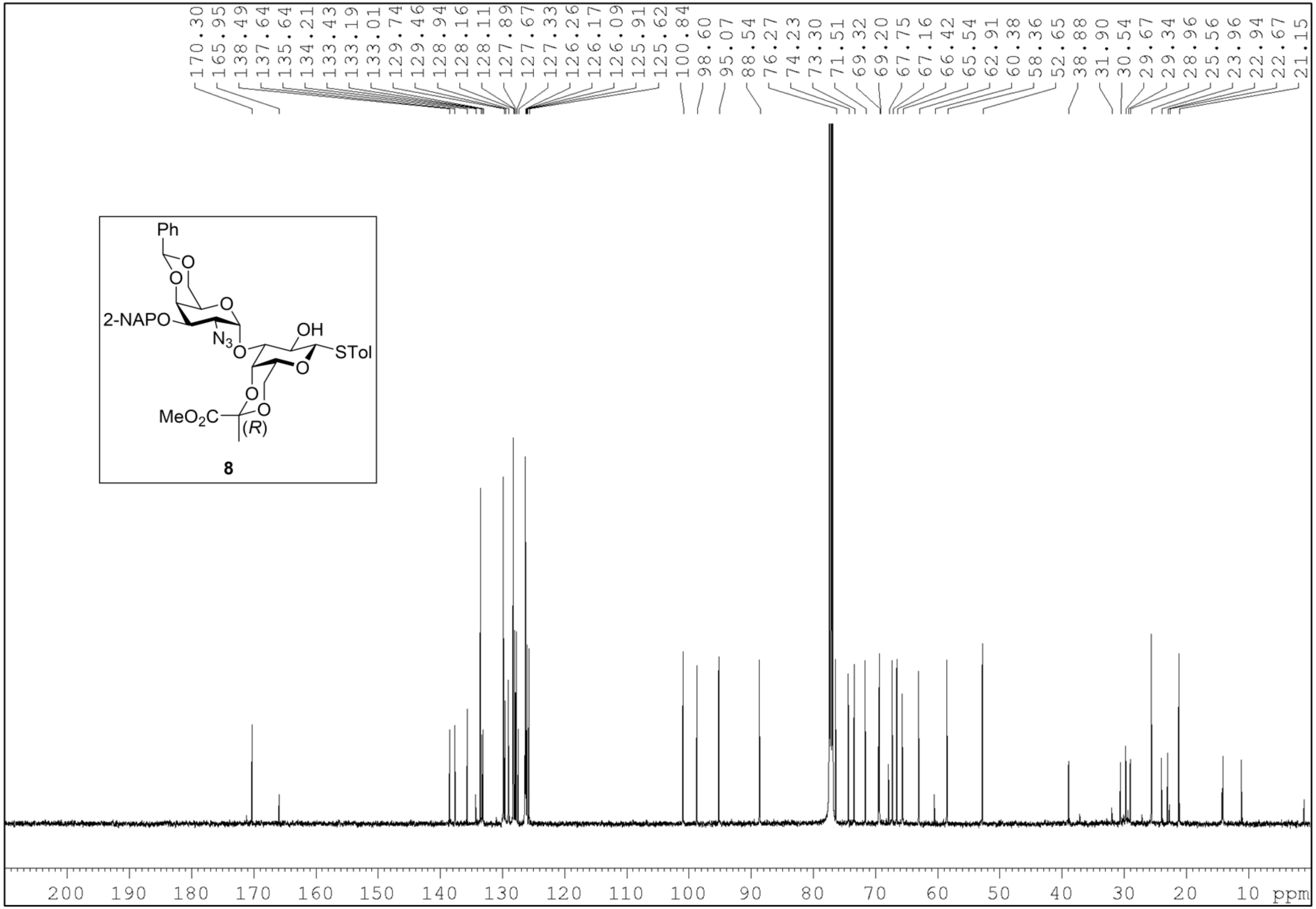

The ^{13}C spectrum in $CDCl_3$ of compound 8.

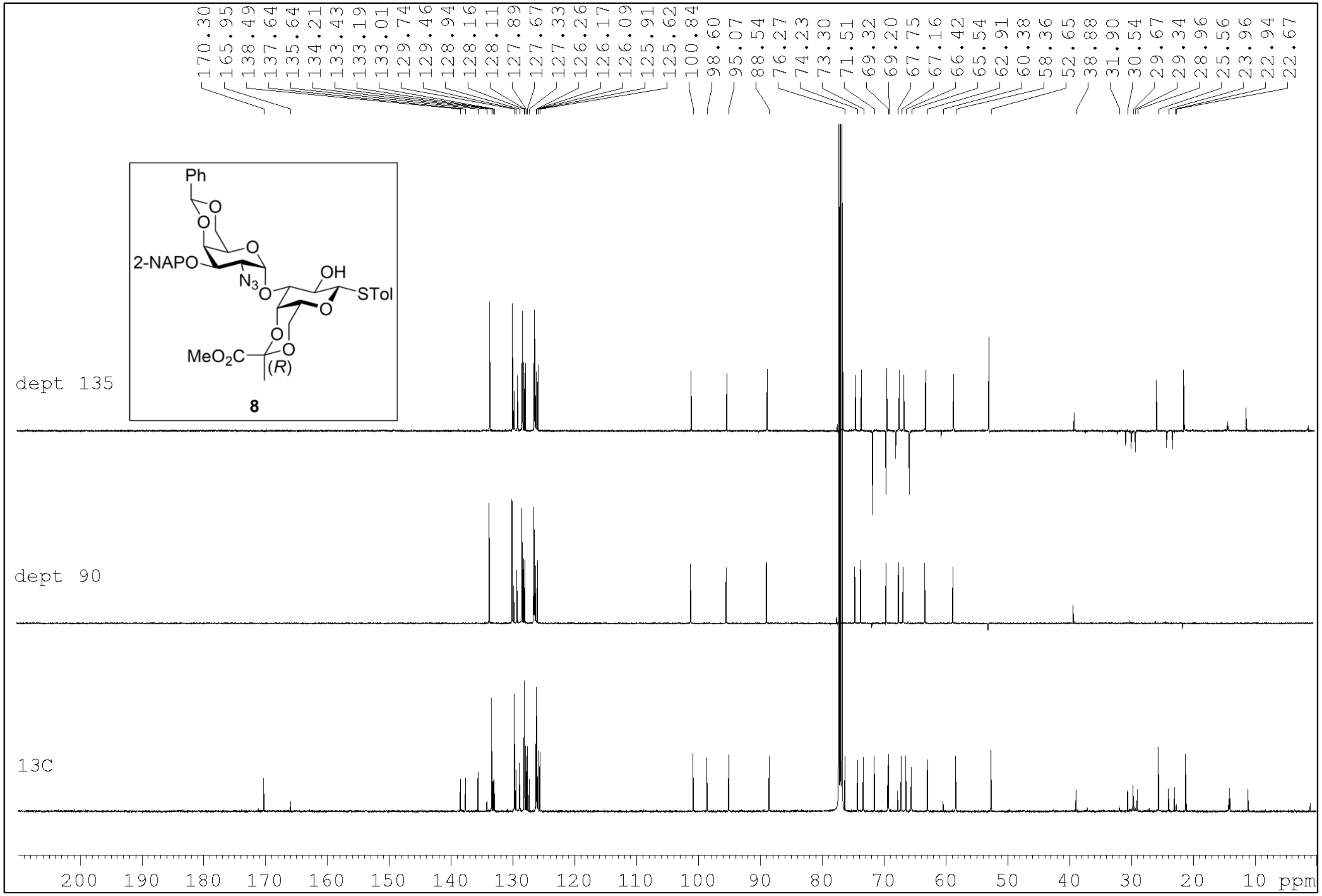

The ^{13}C spectrum in $CDCl_3$ of compound 8.

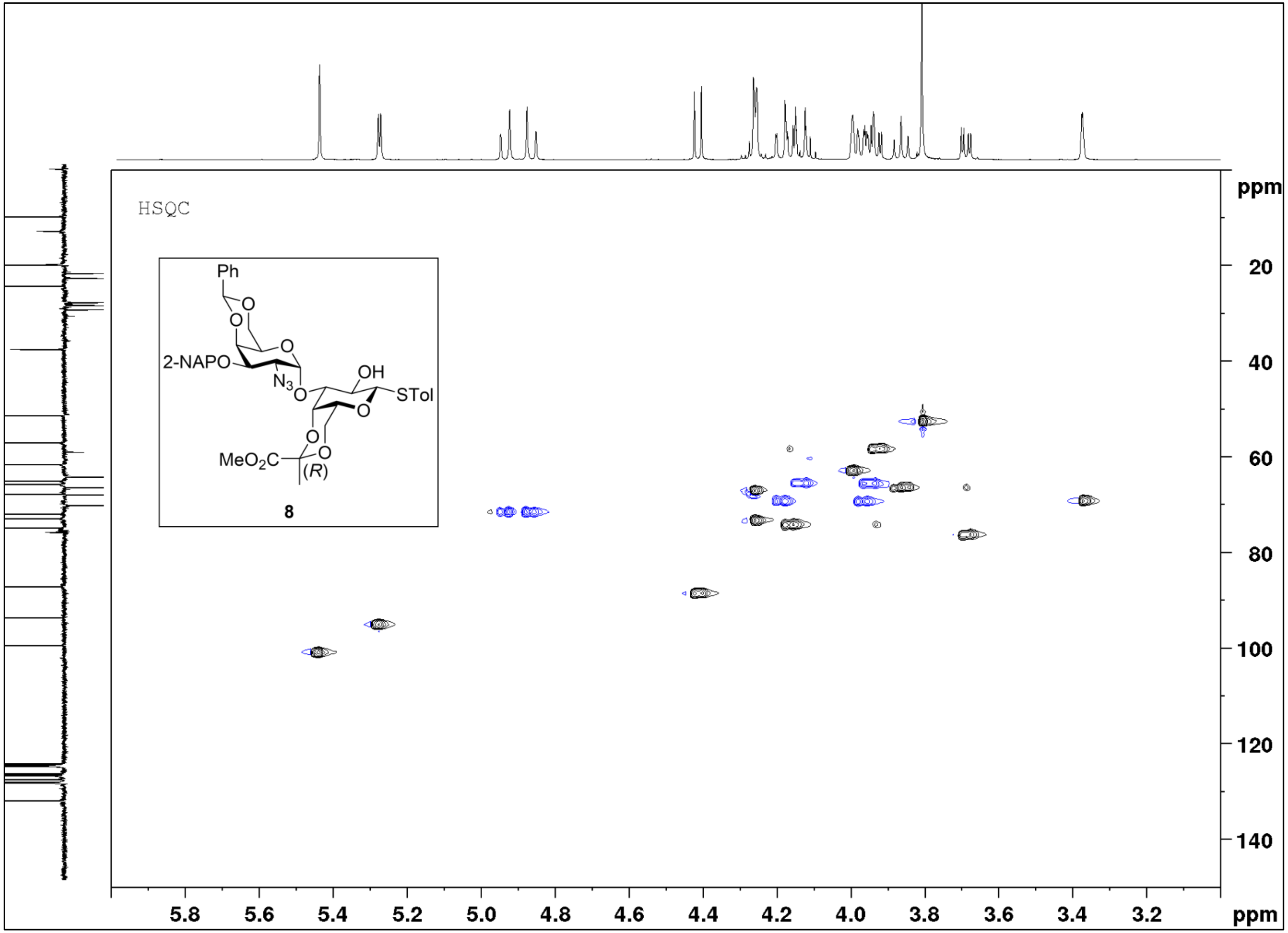

The ^{1}H-^{13}C HSQC spectrum in $CDCl_3$ of compound 8.

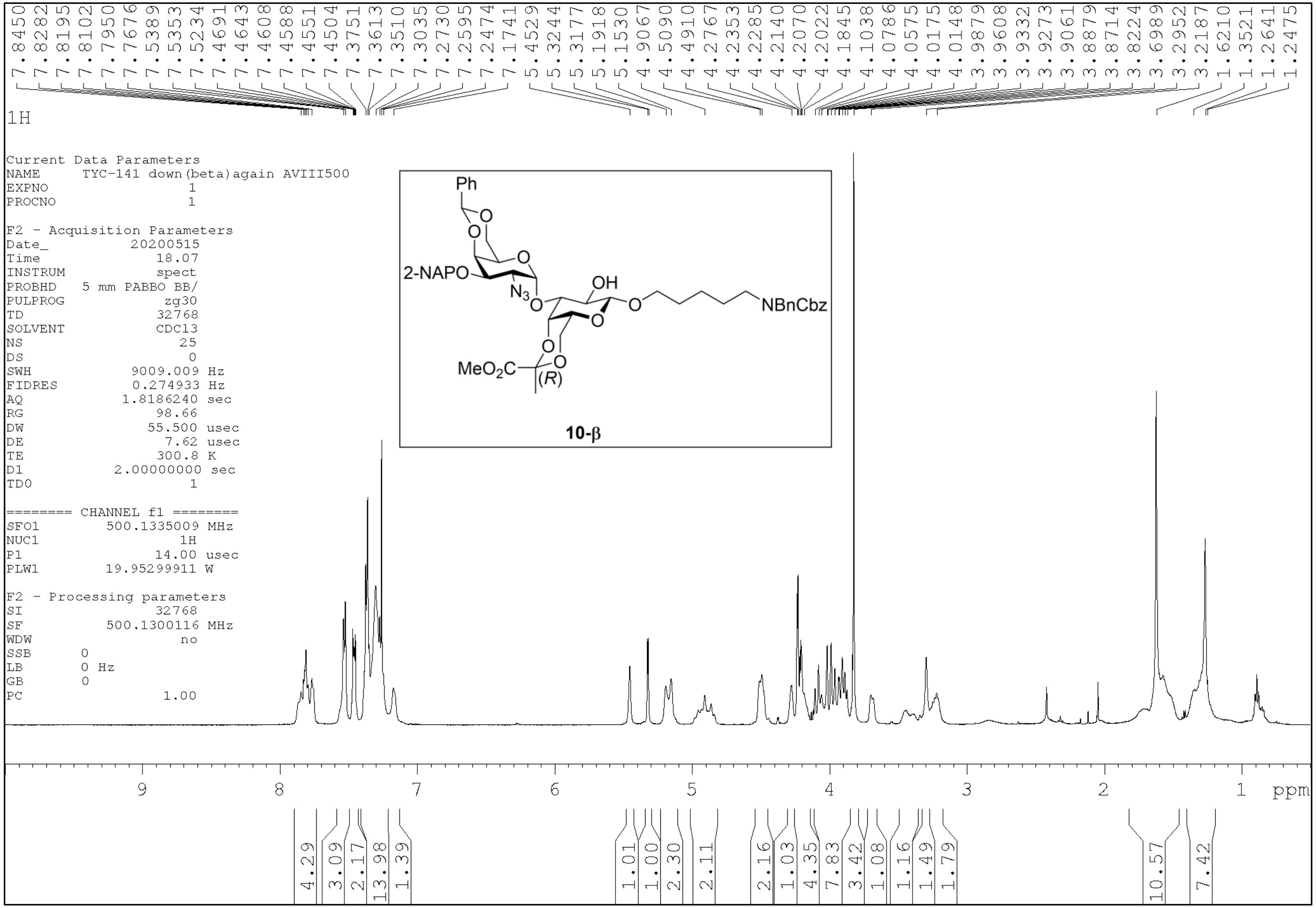

The ^{1}H spectrum in $CDCl_3$ of compound 10-β.

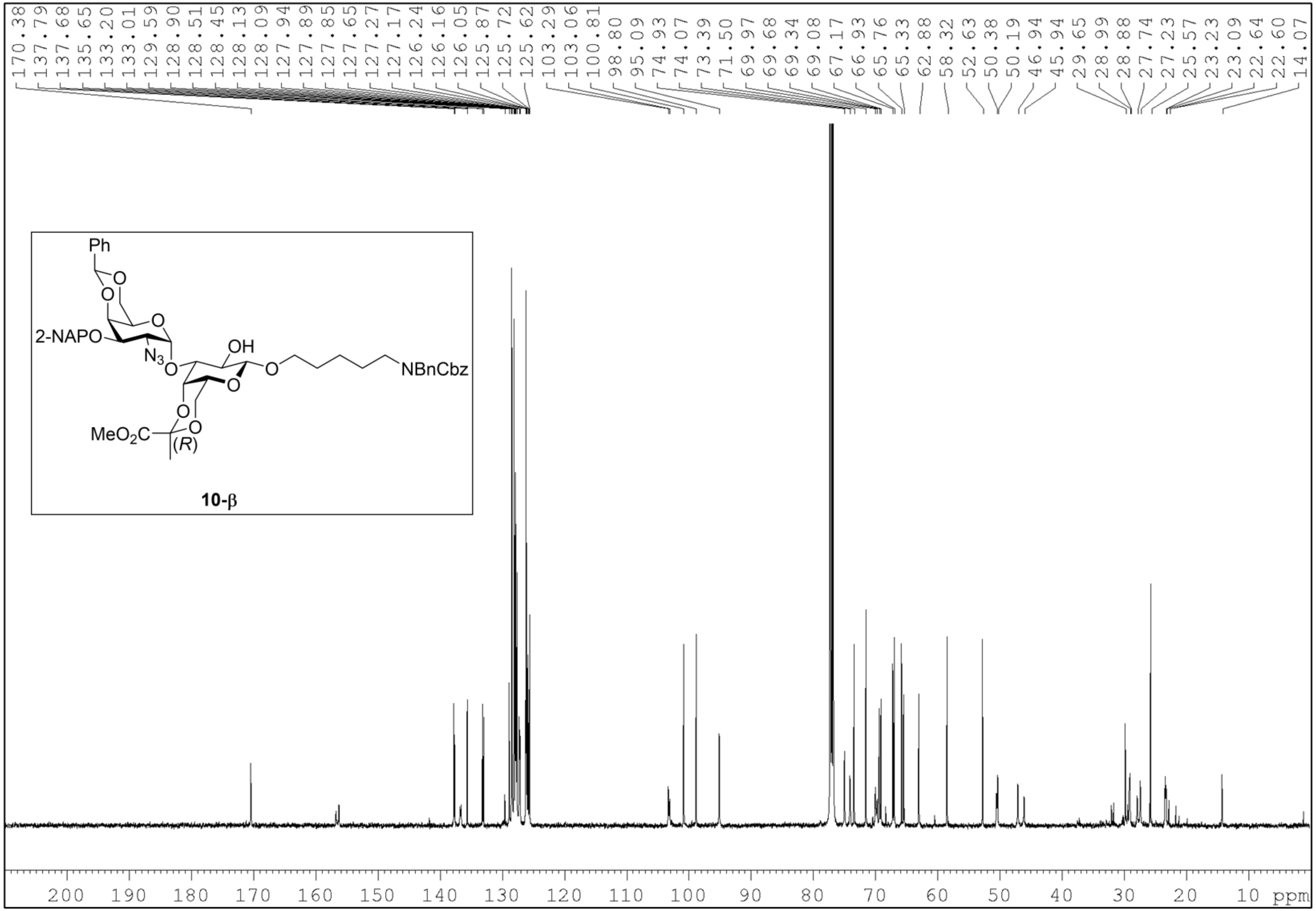

The ^{13}C spectrum in $CDCl_3$ of compound 10-β.

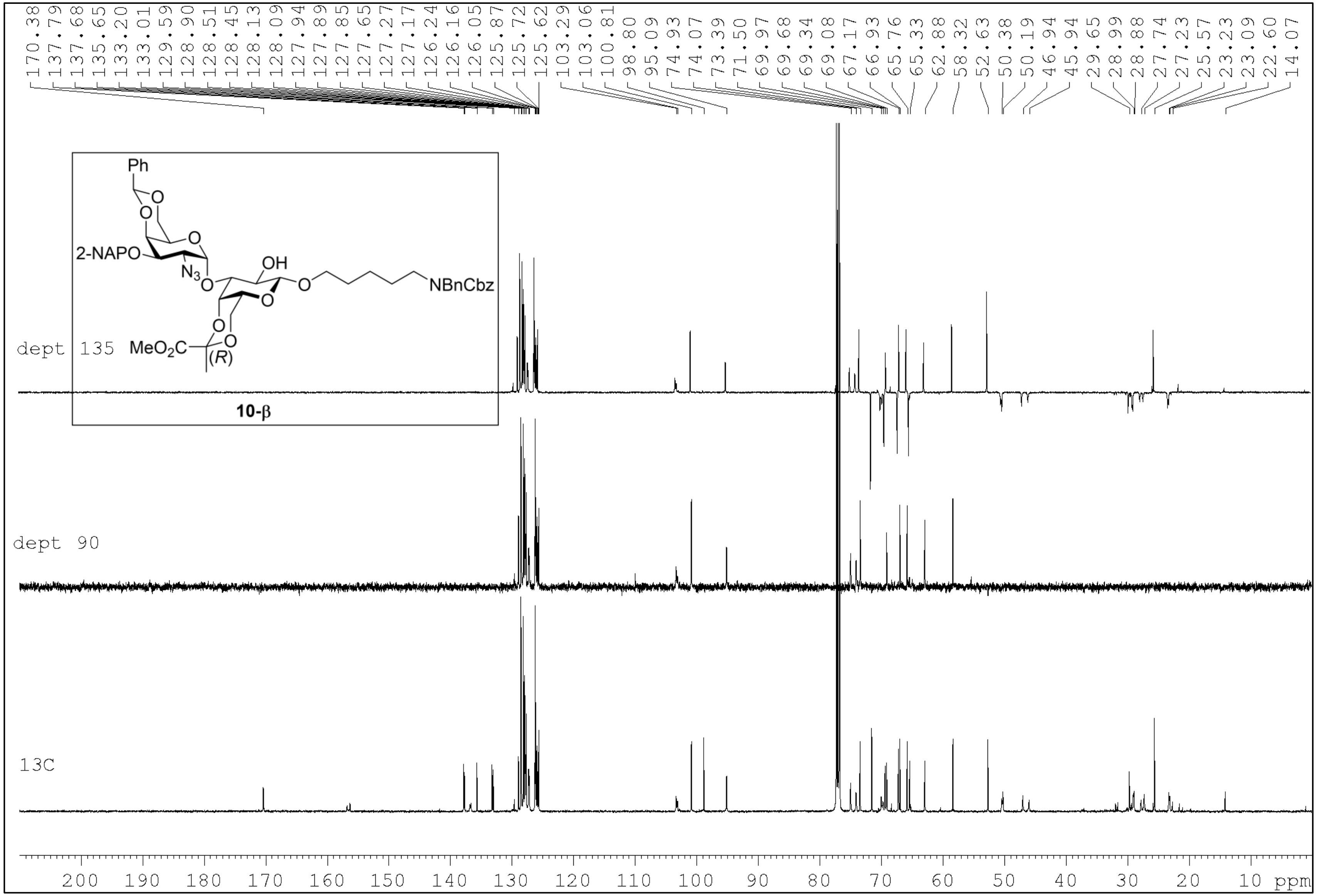

The ^{13}C spectrum in $CDCl_3$ of compound 10-β.

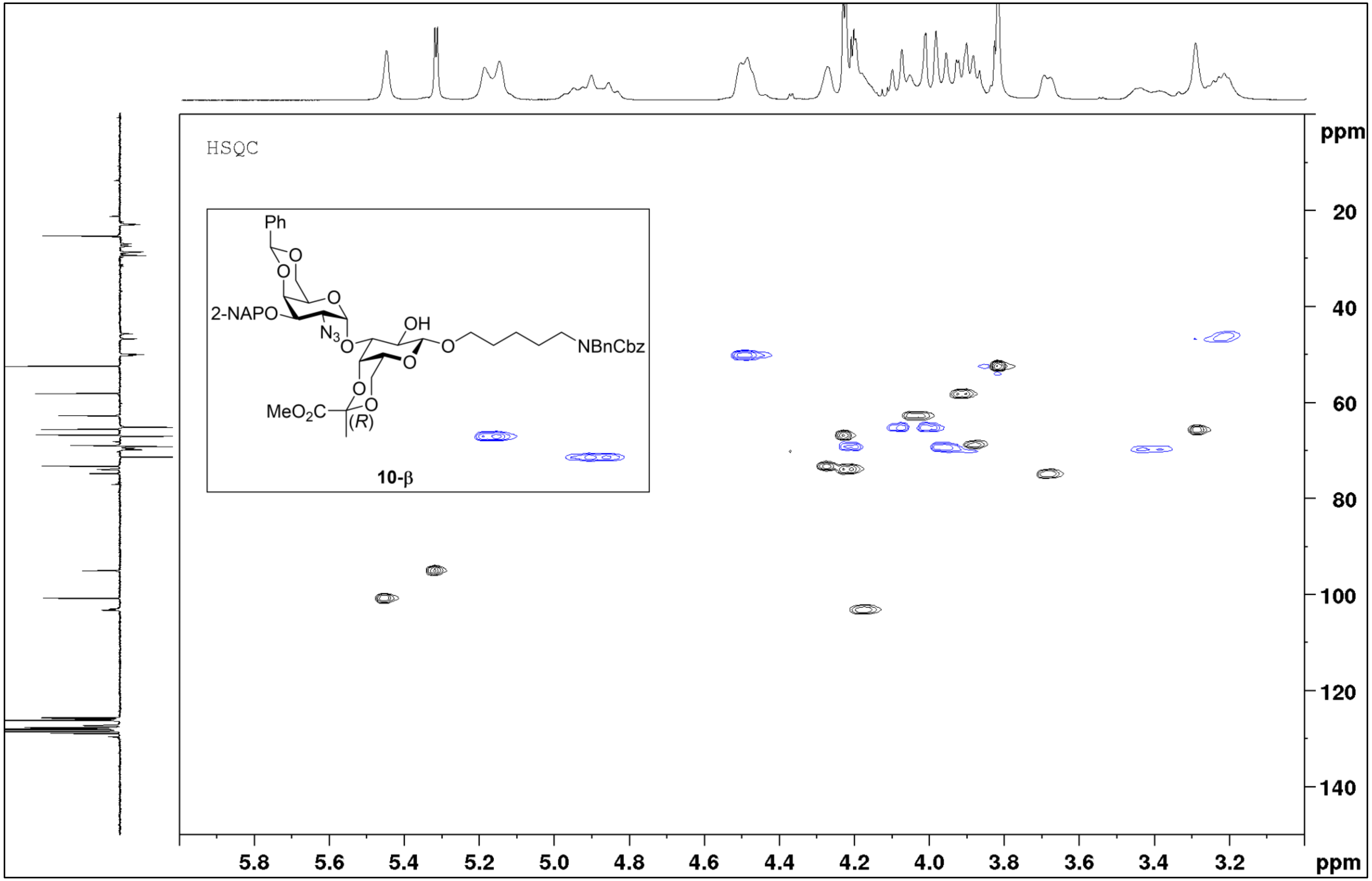

The ^{1}H-^{13}C HSQC spectrum in $CDCl_3$ of compound **10-β**.

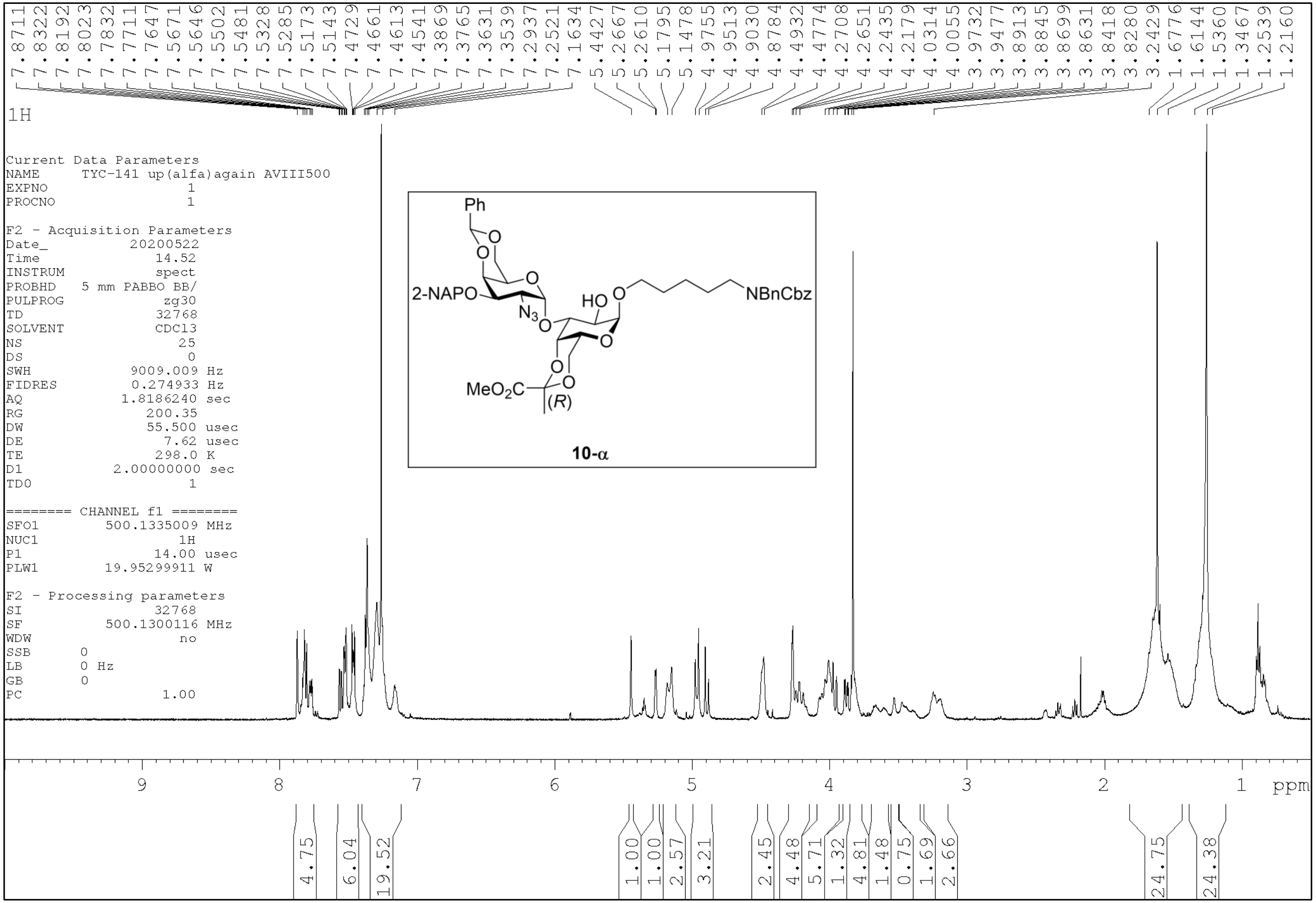

The ^{1}H spectrum in $CDCl_3$ of compound 10-α.

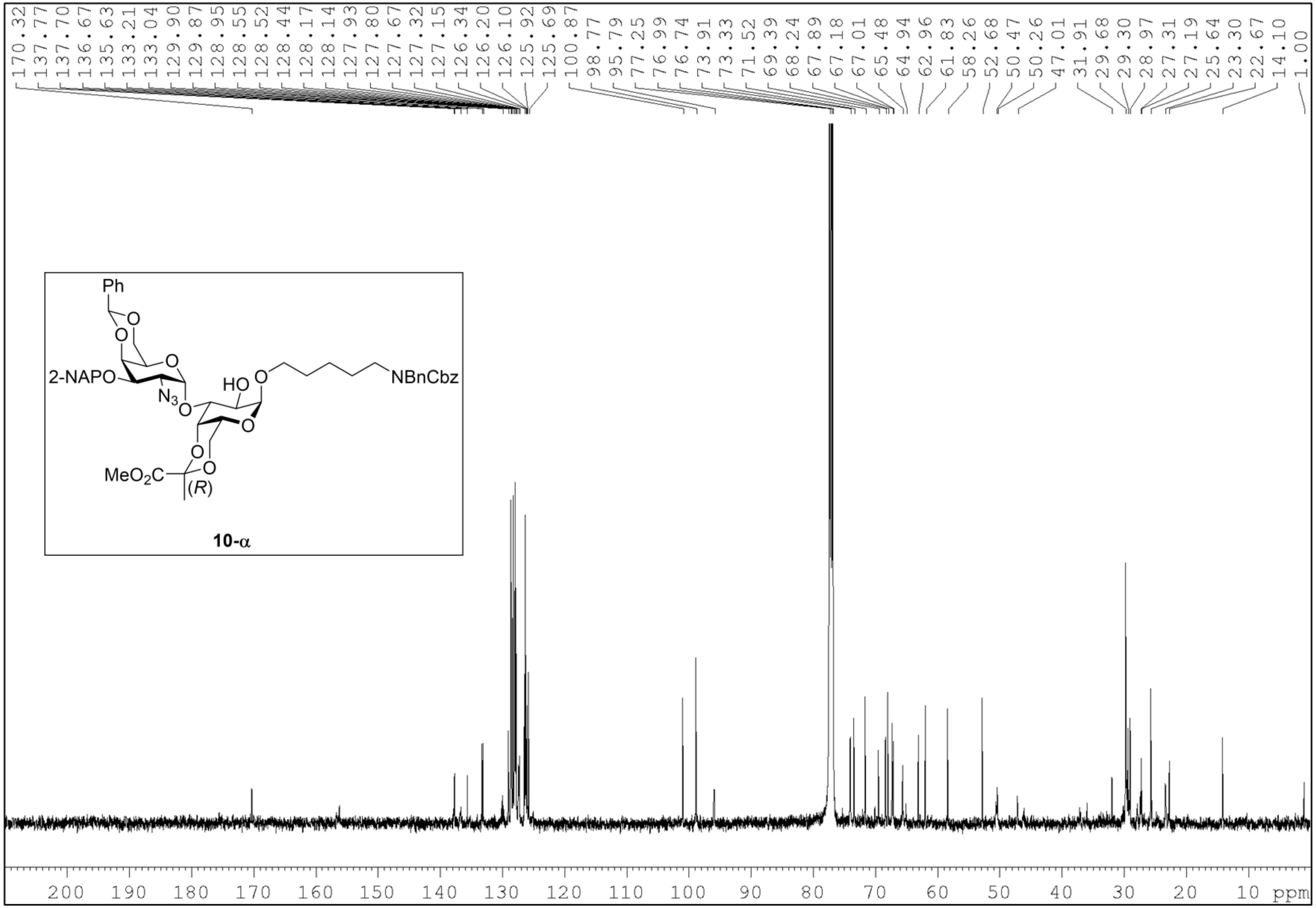

The ^{13}C spectrum in $CDCl_3$ of compound 10-α.

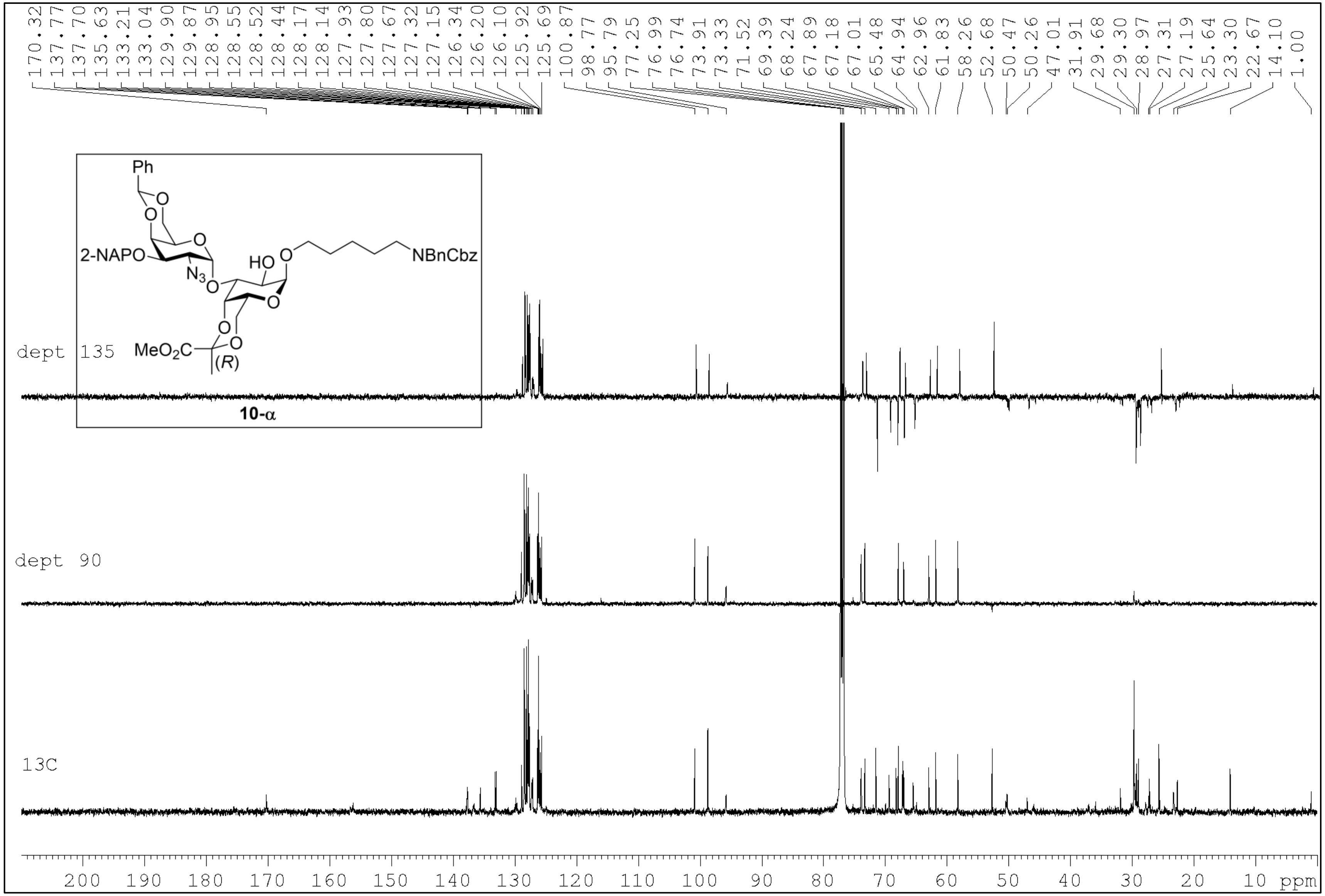

The ^{13}C spectrum in $CDCl_3$ of compound 10-α.

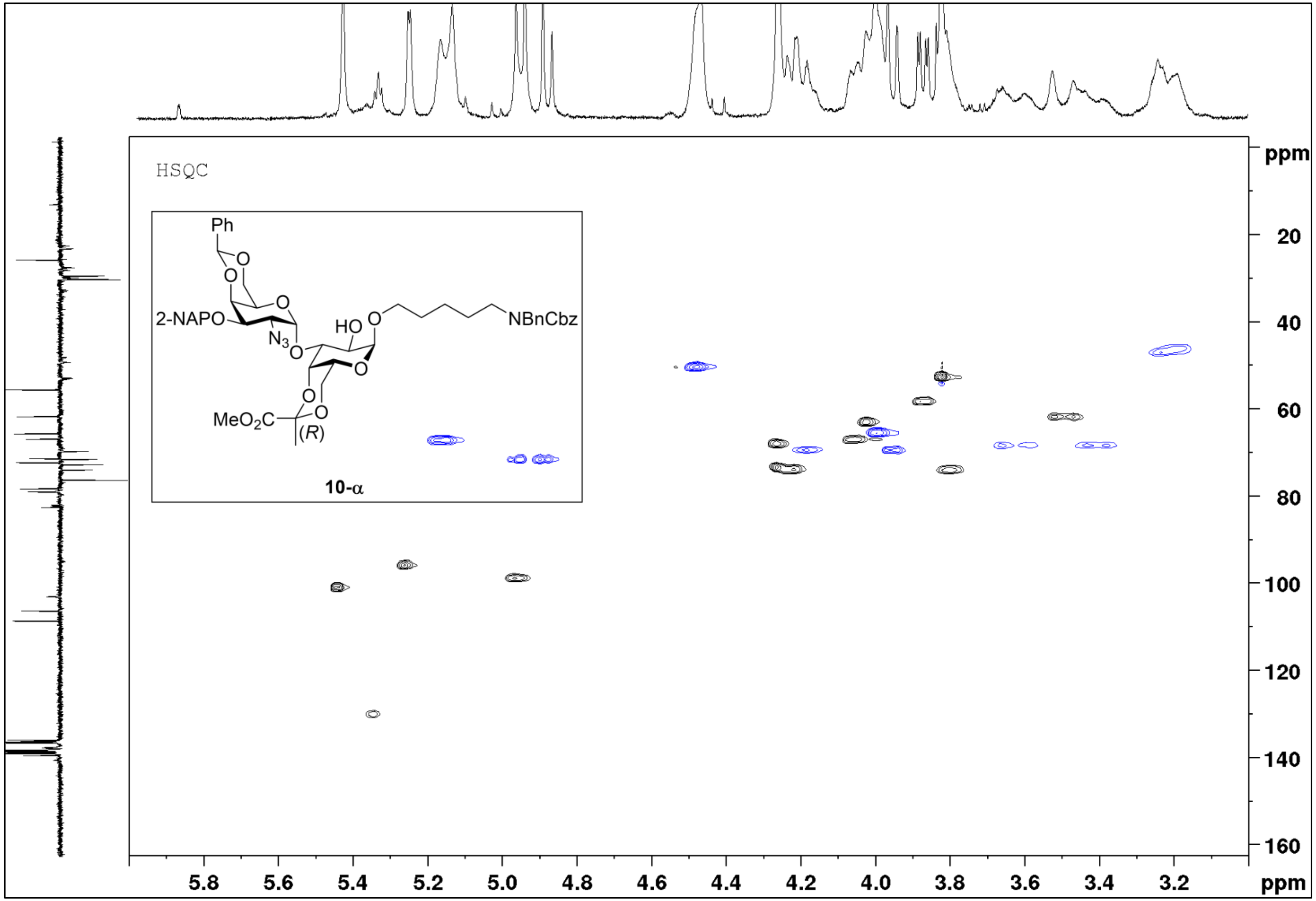

The ^1H-^{13}C HSQC spectrum in $CDCl_3$ of compound 10-α.

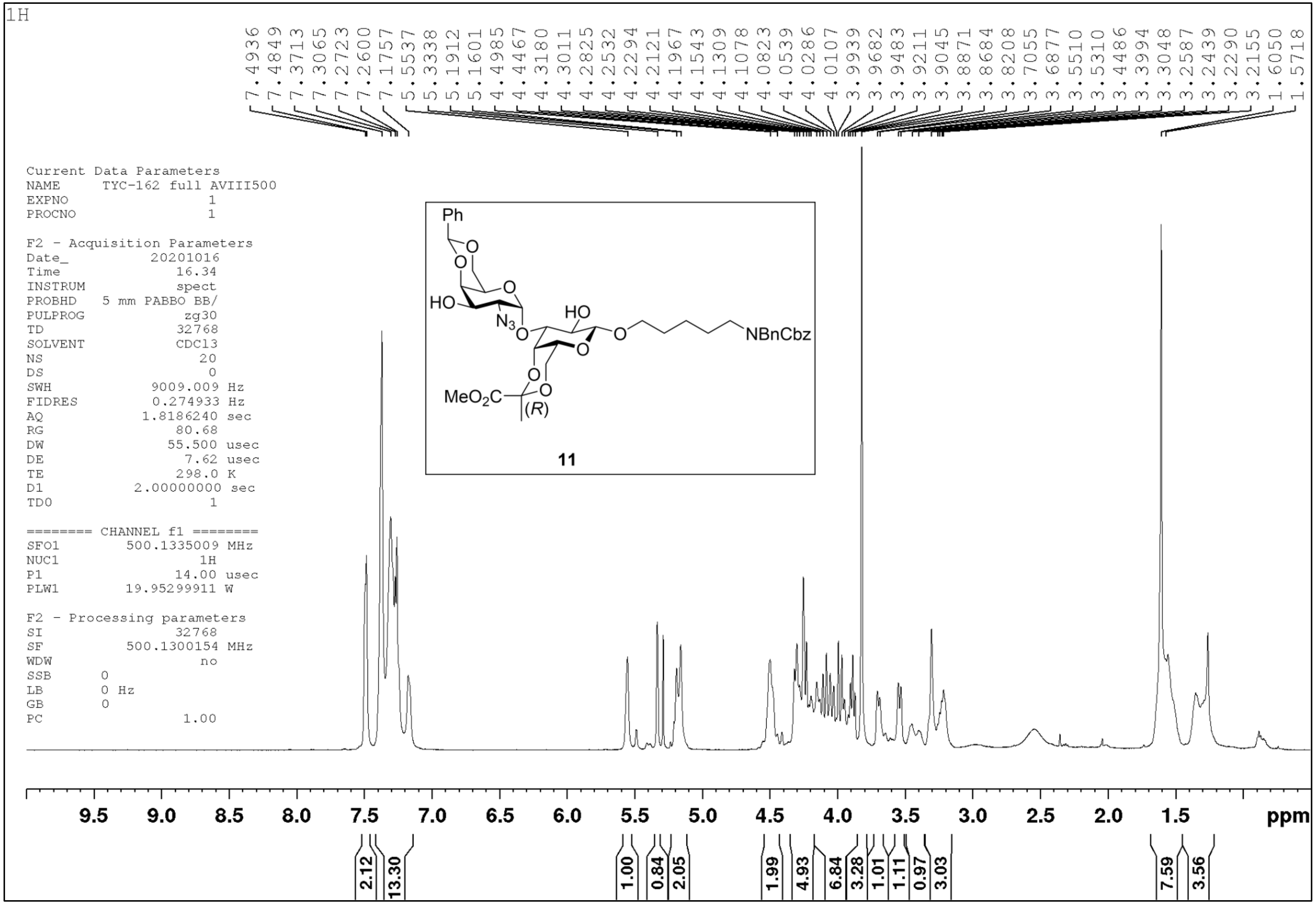

The ^{1}H spectrum in $CDCl_3$ of compound 11.

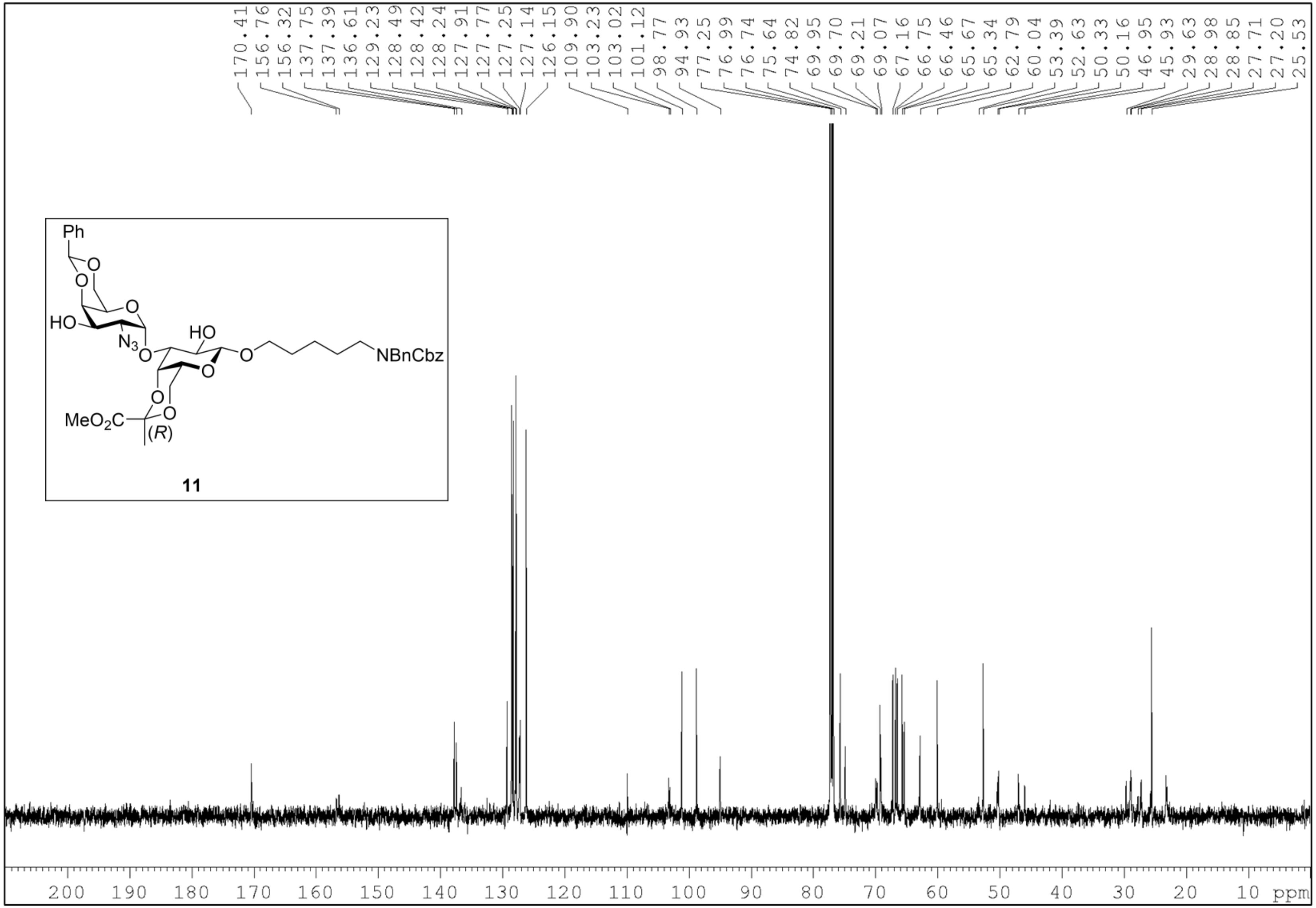

The ^{13}C spectrum in $CDCl_3$ of compound 11.

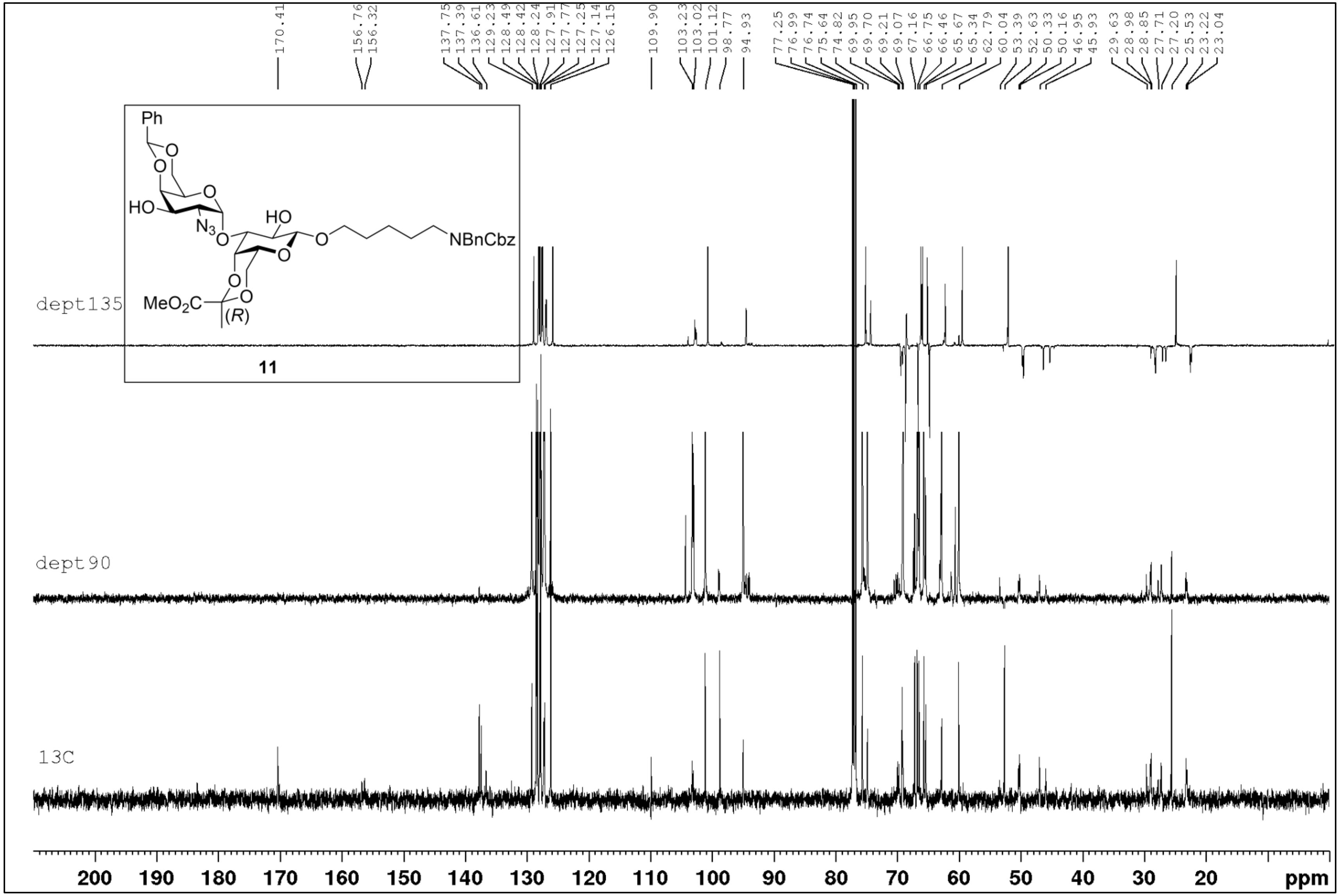

The ^{13}C spectrum in $CDCl_3$ of compound 11.

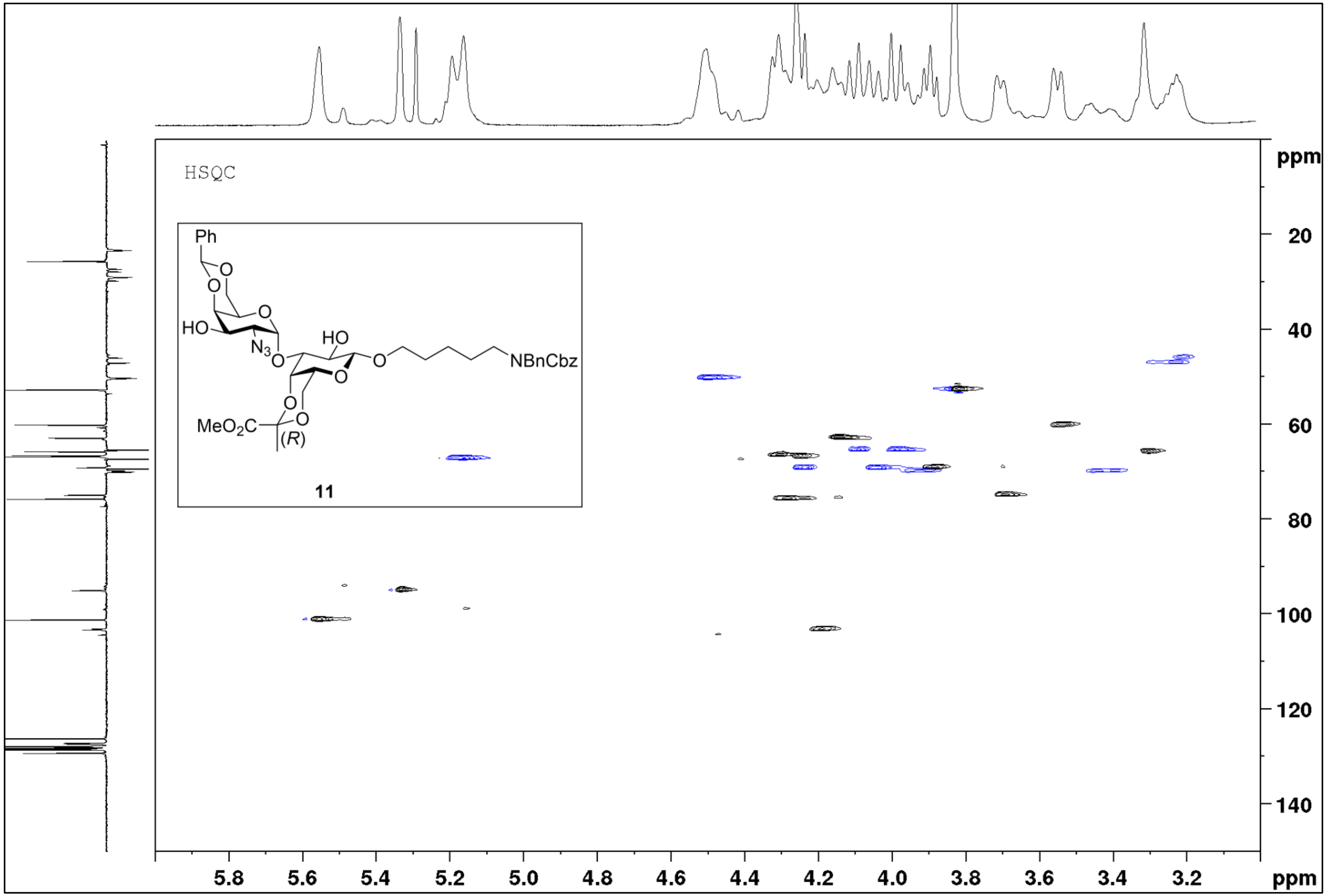

The ^1H-^{13}C HSQC spectrum in $CDCl_3$ of compound 11.

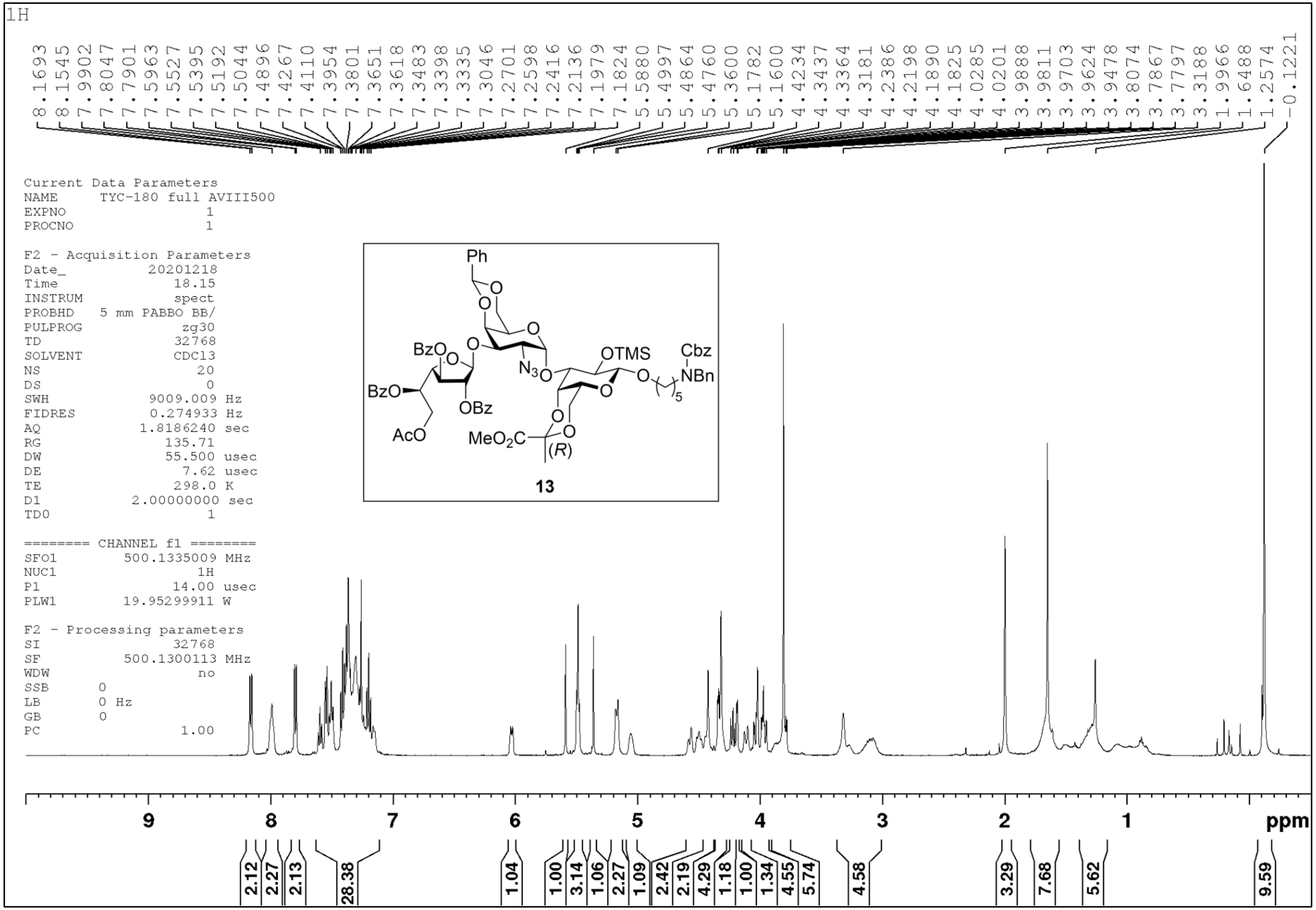

The ^{1}H spectrum in CDCl3 of compound 13.

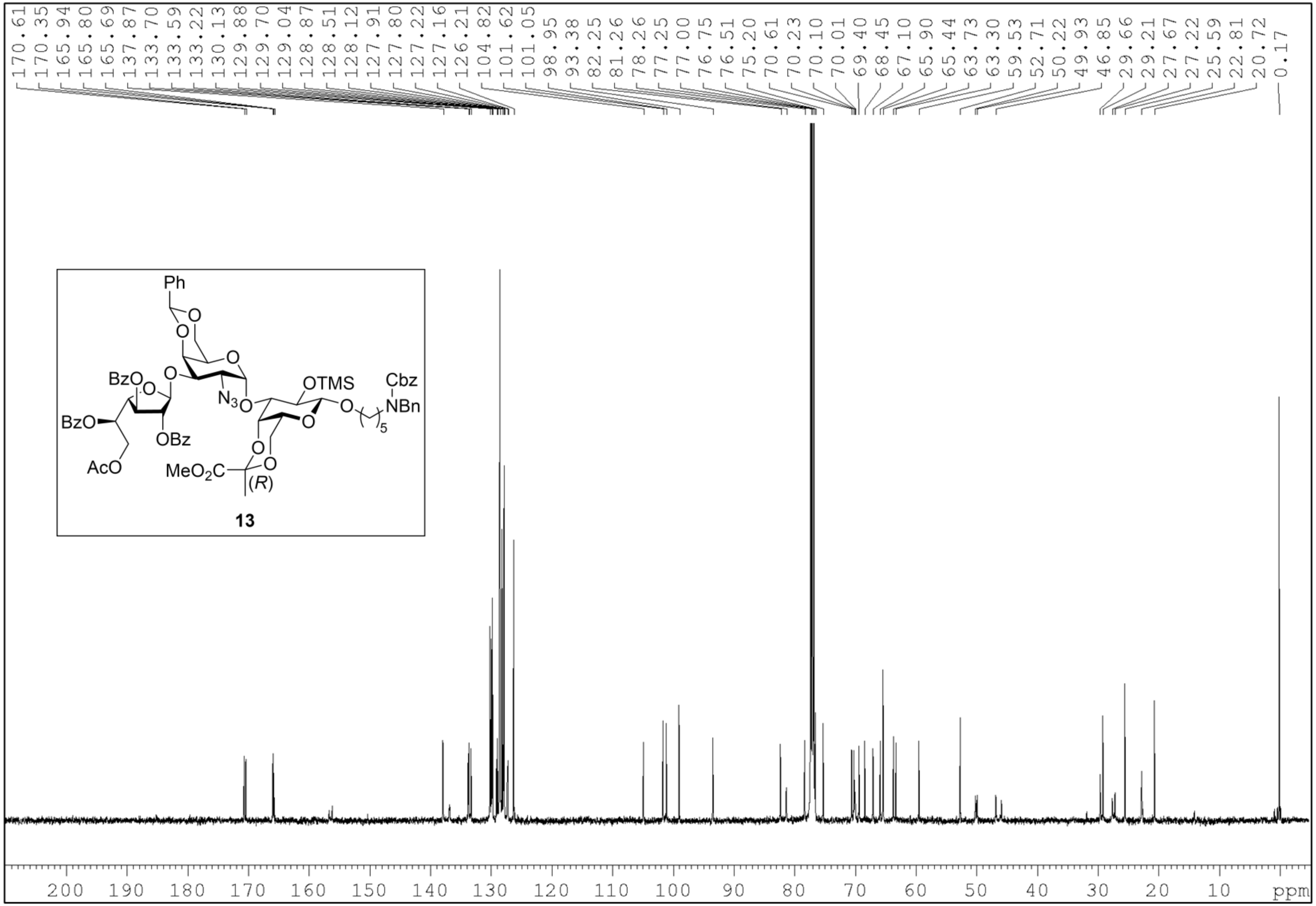

The ^{13}C spectrum in $CDCl_3$ of compound 13.

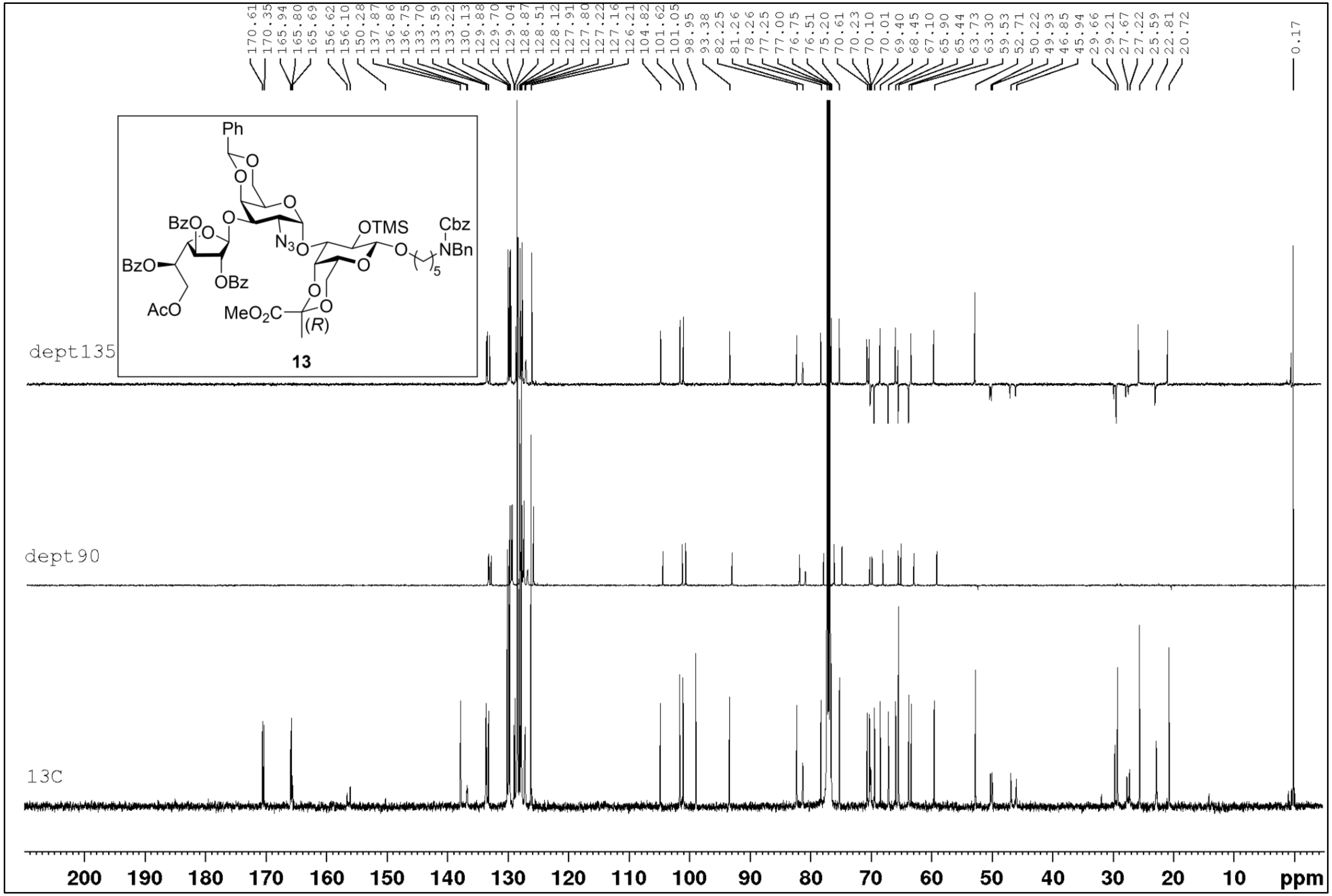

The ^{13}C spectrum in $CDCl_3$ of compound 13.

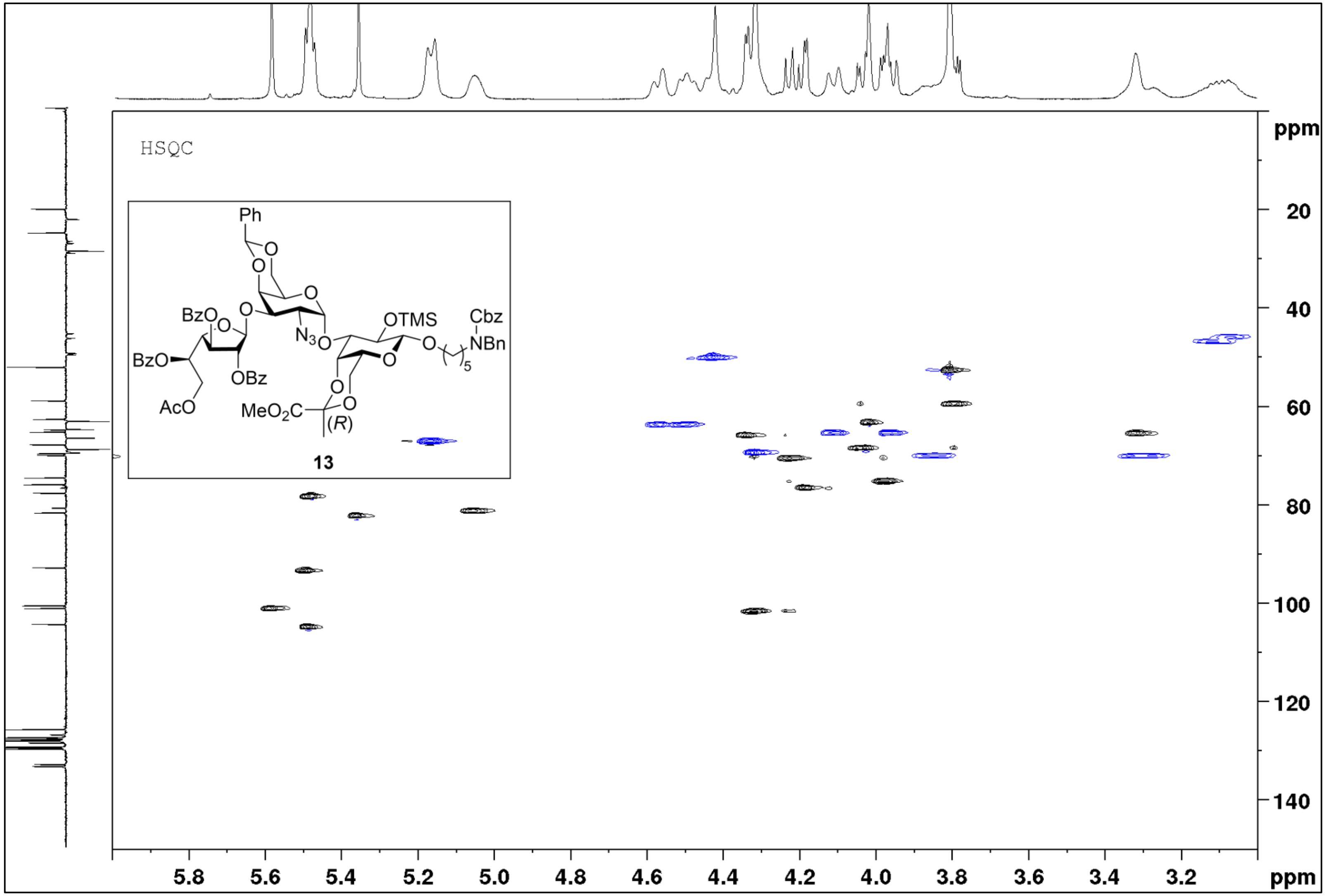

The ^{1}H-^{13}C HSQC spectrum in $CDCl_3$ of compound 13.

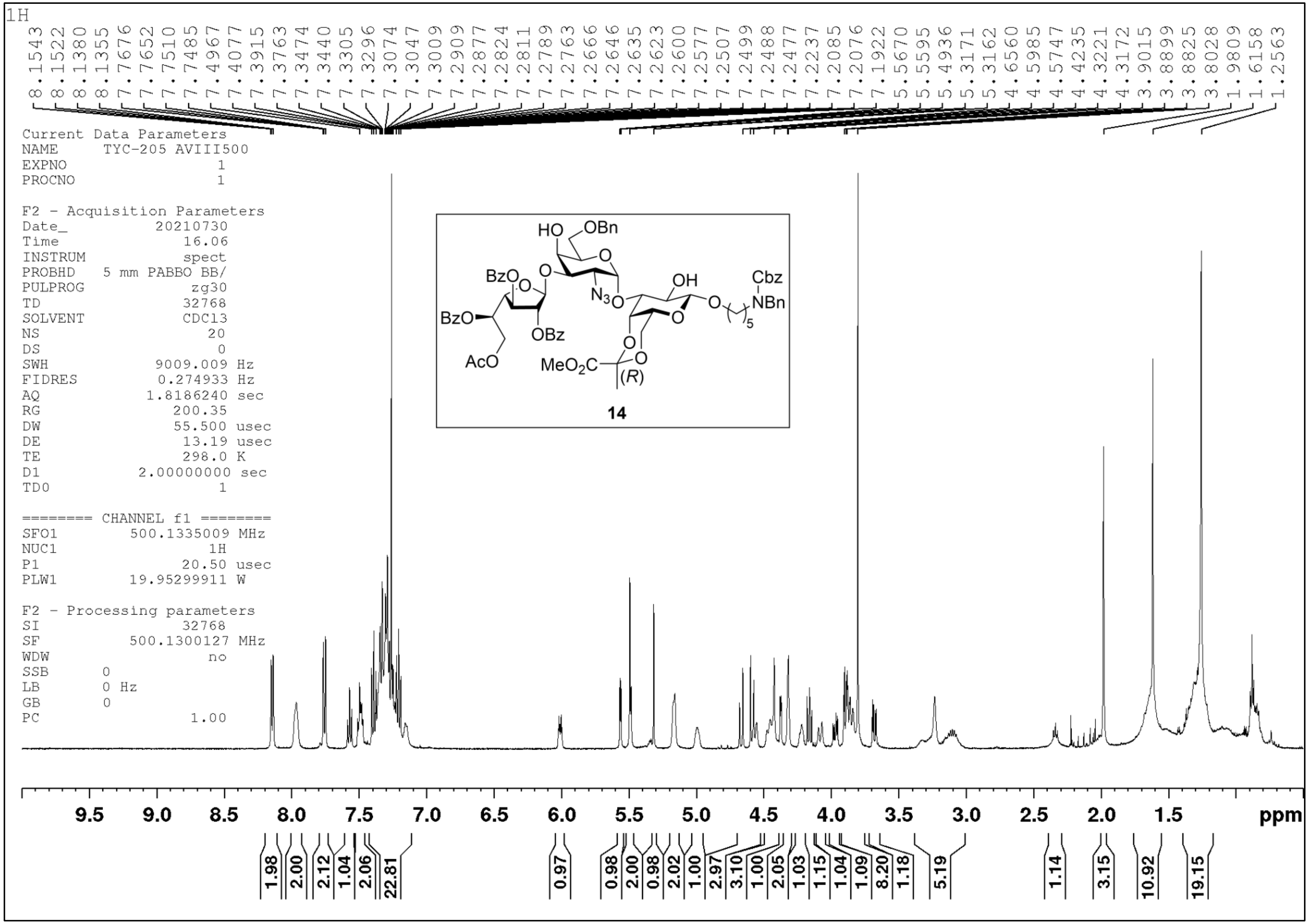

The ^1H spectrum in $CDCl_3$ of compound 14.

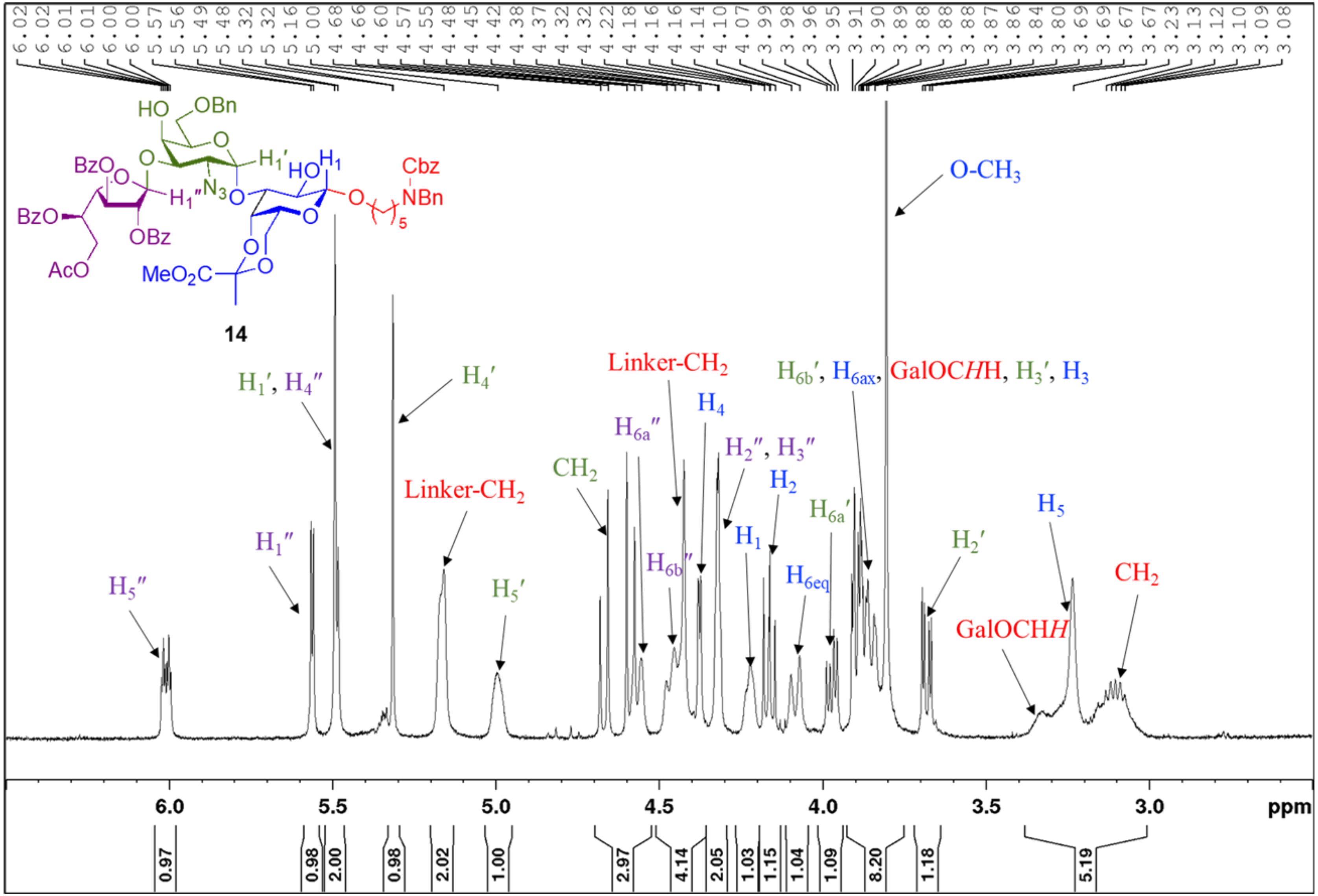

The ^{1}H spectrum in $CDCl_3$ of compound 14.

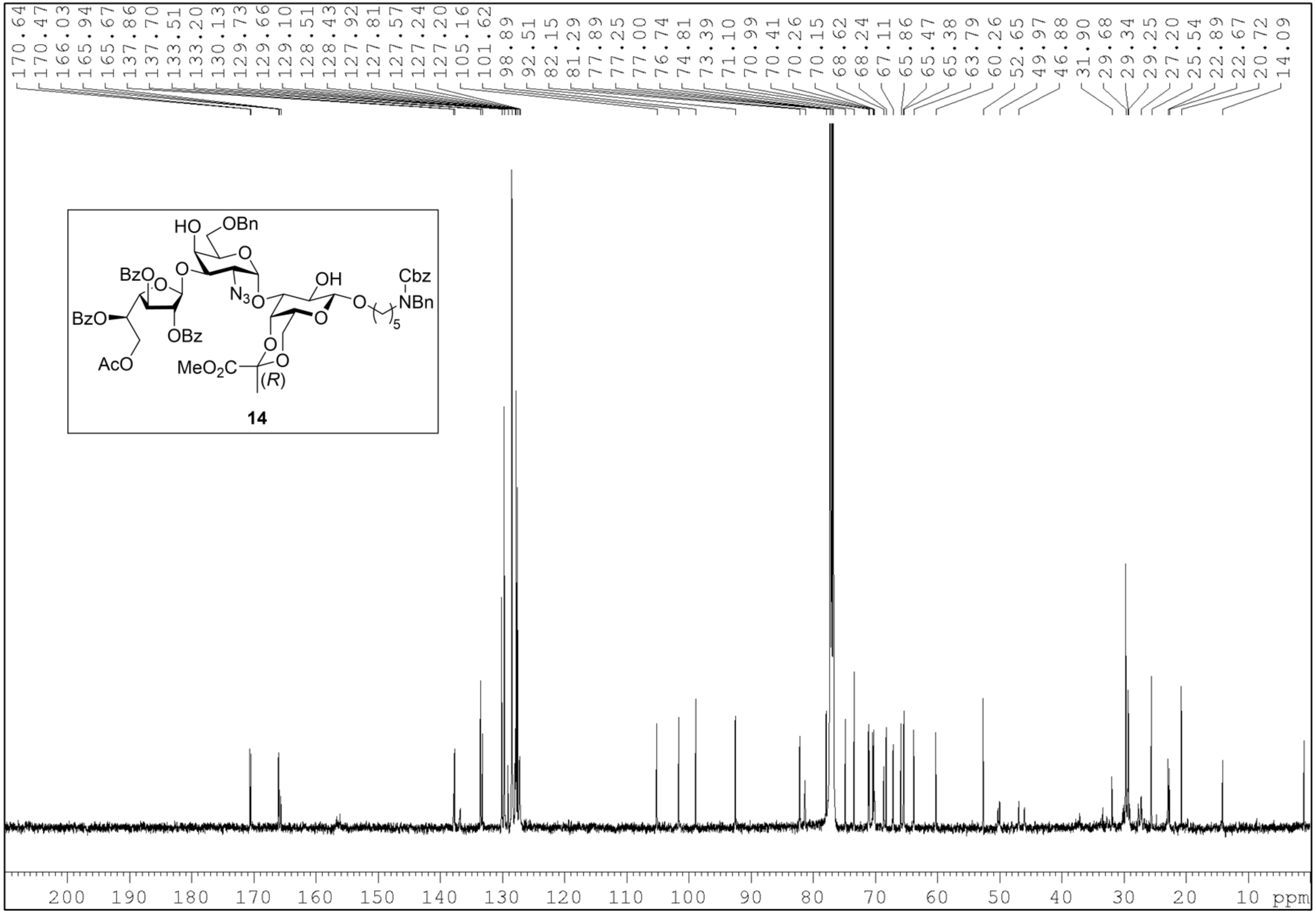

The ^{13}C spectrum in $CDCl_3$ of compound 14.

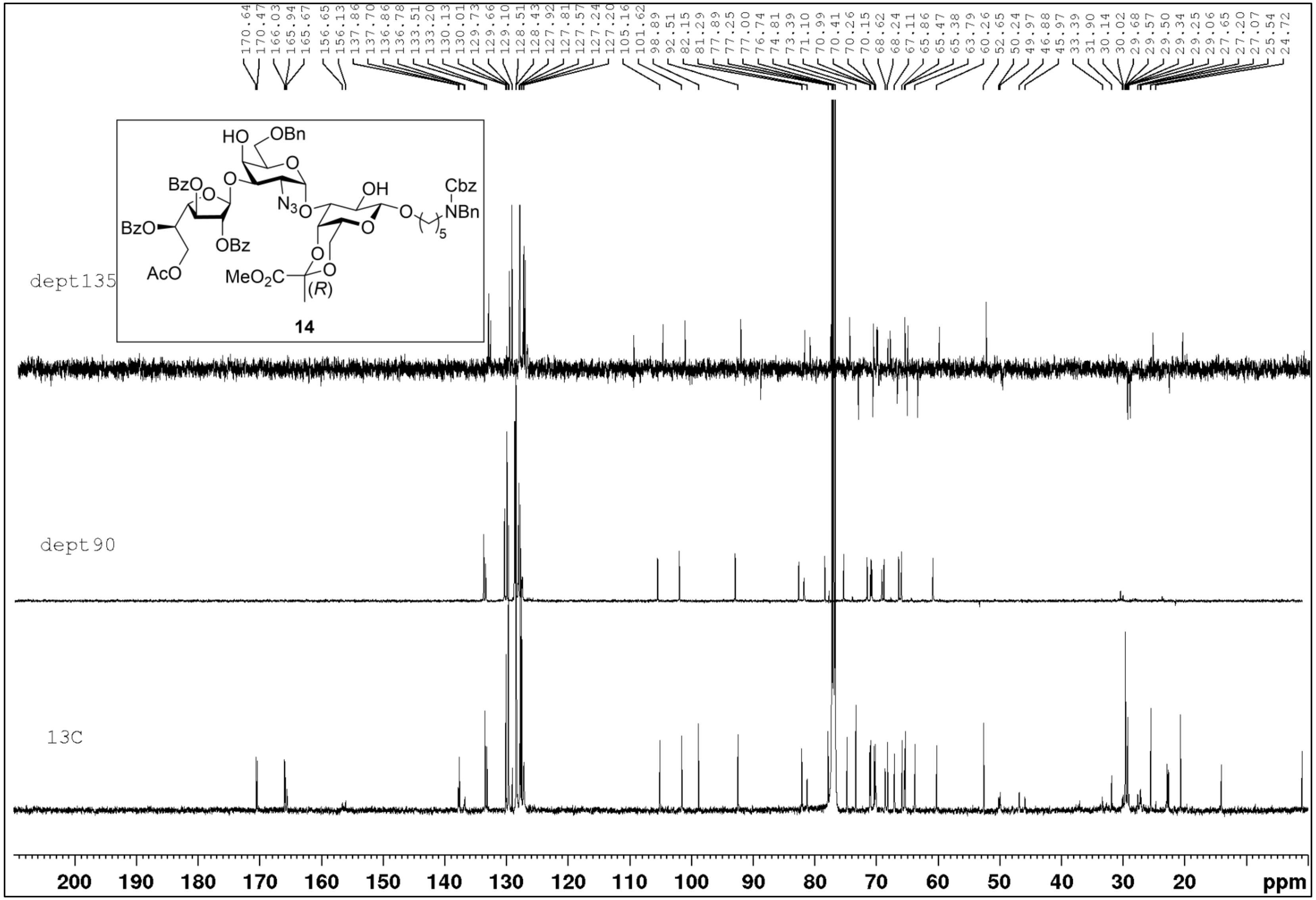

The ^{13}C spectrum in $CDCl_3$ of compound 14.

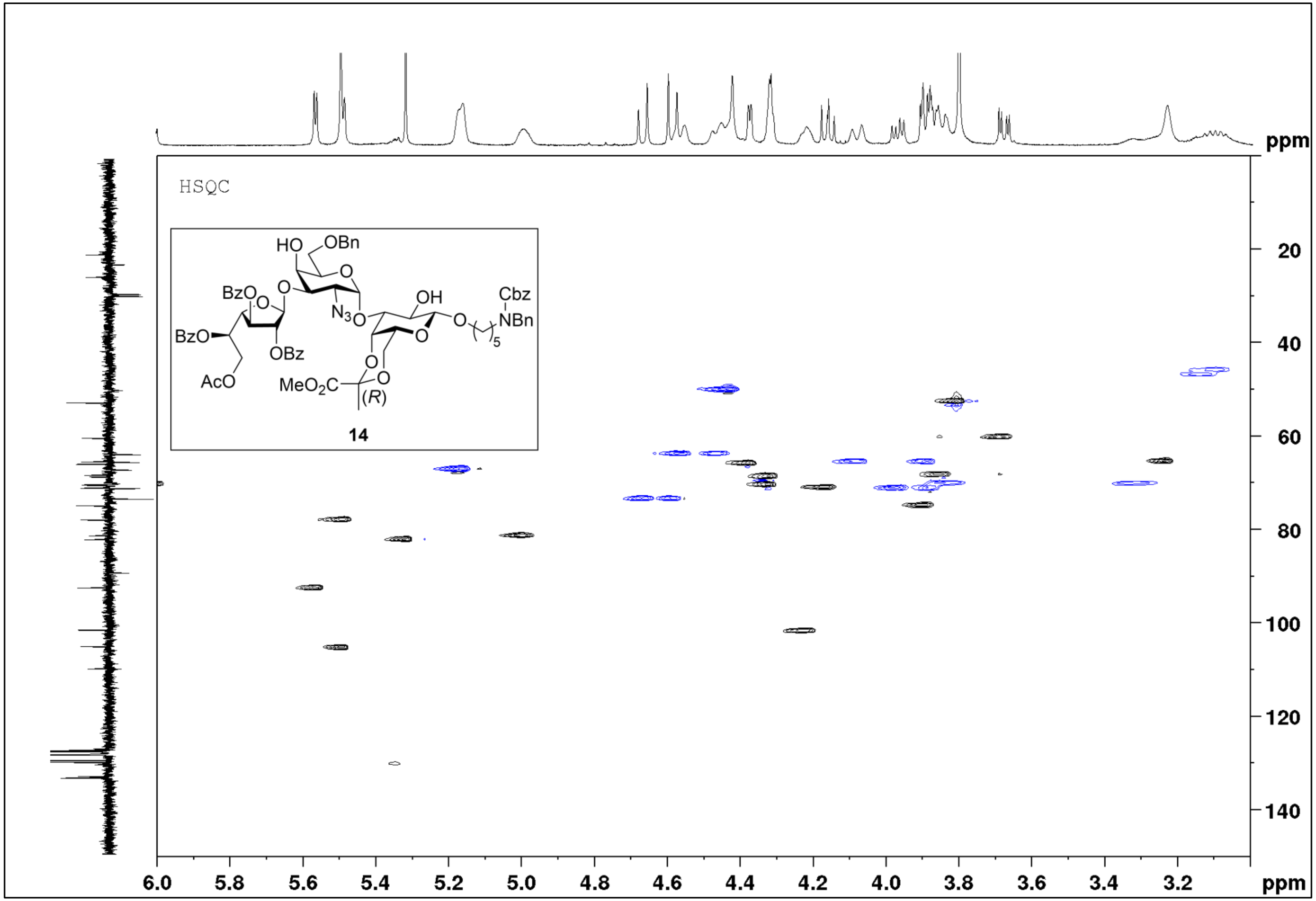

The ^{1}H-^{13}C HSQC spectrum in $CDCl_3$ of compound 14.
